# Skeletal muscle transcriptome is affected by age in severely burned mice

**DOI:** 10.1038/s41598-022-26040-1

**Published:** 2022-12-14

**Authors:** Juquan Song, Steven G. Widen, Steven E. Wolf, Amina EI Ayadi

**Affiliations:** 1 Department of Surgery, University of Texas Medical Branch, 301 University Blvd., Galveston, TX 77555-0644 USA; 2 Department of Biochemistry and Molecular Biology, University of Texas Medical Branch, Galveston, TX USA

**Keywords:** Trauma, Transcriptomics, Skeletal muscle, Experimental models of disease, Paediatric research

## Abstract

Severe burn results in muscle wasting affecting quality of life in both children and adults. Biologic metabolic profiles are noticeably distinctive in childhood. We posit that muscle gene expression profiles are differentially regulated in response to severe burns in young animals. Twelve C57BL6 male mice, including young (5 weeks-old) and adults (11 weeks-old), received either scald burn, or sham procedure. Mouse muscle tissue was harvested 24 h later for Next Generation Sequence analysis. Our results showed 662 downregulated and 450 upregulated genes in gastrocnemius of young mice compared to adults without injury. After injury, we found 74/75 downregulated genes and 107/128 upregulated genes in both burned groups compared to respective uninjured age groups. VEGFA-VEGFR2, focal adhesion, and nuclear receptor meta-pathways were the top 3 gene pathways undergoing a differential change in response to age. Of note, the proteasome degradation pathway showed the most similar changes in both adult and young burned animals. This study demonstrates the characteristic profile of gene expression in skeletal muscle in young and adult burned mice. Prominent age effects were revealed in transcriptional levels with increased alterations of genes, miRNAs, pathways, and interactions.

## Introduction

Adolescents from 10 to 19 years of age are in a transition from childhood to adulthood, and are at higher risk of non-intentional injury. According to the CDC childhood injury report, more than 9.2 million injured are treated in hospital emergency rooms, and more than 12,000 injured people under 19-year-old die each year. Burn is one of the leading causes of unintentional injury leading to higher morbidity and mortality among children in the US and worldwide. The proportion of pediatric admissions among total patients with burn injury increased by 63.9% from 2003 to 2012 at United States^1^. Meanwhile, the WHO investigated burn incidence in children and reported 96,000 lethal burn injuries globally in 2004^2^. These death rates are the second-highest between 15 and 19 years. old children after infants. Worldwide, an increasing proportion of burn injuries was recorded in adolescent females^2^. Along with the improvement of care management, current mortality has been reduced to less than 3% in pediatric burn patients^3^. However, severe burn has significant impact and consequences in surviving children, including both physical disfiguration and mental in long-term effects. Burn impairs growth in children^4^, and has other effects after injury^5^.

The mechanisms of burn-induced muscle wasting are mostly described within the hypermetabolic response to severe burn, significantly contributing to sequelae after injury. Various treatments were developed to moderate the severity of this pathophysiological and metabolic response^6^. At the transcriptional level, Padfield et al. showed that groups of genes, including those regulating myogenesis and mitochondrial function, contributed to muscle wasting in a mouse model with a single limb burn^7^. Only a few studies further revealed gene expression changes in pediatric burns during a long-term period of recovery^8^. 

As key gene regulators, microRNAs (miRNAs) inhibit gene expression at the translational level^9^. In burns, Liang et al. demonstrated miRNA profiles in cells within the burned dermis and found that 66 miRNAs were significantly altered^10^. We found miRNAs to mediate gene pathway changes in skeletal muscle after burn^11^. However, the data collected from most studies (including ours) were based on adult animal models, which may not reflect responses in adolescents and young subjects, and can lead to a bias in data interpretation when applied to pediatric patient populations.

The biological profile in adolescents (< 19 years old) is unique and different from adults^12^. Related to burns, children have more skin surface area in the head and less in the legs generally^13^. Also, energy metabolism differs among children and adults. Total energy utilization is 60–80 kcal/kg/day for a 10-year-old child and 30–40 kcal/kg/day for a 20-year-old adult^14^. At the cellular level, muscle progenitor satellite cells, a major resource for cell hemostasis, are depleted with age^15^. Genetic regulations also differ in children and adults. A study of 299 adults demonstrated dopamine receptor genes *DRD2* are associated with weight gain in adults, with α-ketoglutarate dependent dioxygenase (*FTO*) gene expression closer to the young adolescence period^16^. We then wonder whether gene expression profiles respond differentially in severe burns in the young compared to adults in the muscle specifically. We then propose that miRNA-regulated gene expression contributes to muscle pathophysiological changes in response to severe burns that are distinct in the young.

To date, the sparse information on differential effects of age in response to injury may be related to the lack of young burn animal models. The life span of rodents differs from humans, reaching maturity in 3–6 months in mice. 5–8 week-old mice are then equivalent to human adolescents aged 12–18 years^17^. Comparing 5 week-old (adolescent) to 11 week-old mice (adult) in this study, we examined miRNA and mRNA age-related expression profiles in mouse skeletal muscle.

## Results

### General description of transcriptome alteration in young and adult burn mice

Among a total of 55,401 grand counts, 21,859 protein-coding genes and 2,202 miRNA counts were deferentially expressed in mouse muscle tissue, reflecting a proportional alteration of 39.45% genes and 3.97% miRNA in the total cohorts. These data reinforce previous findings suggesting a small number of miRNAs are present to regulate expression/function of a large number of target genes/mRNA.

**Figure 1:**
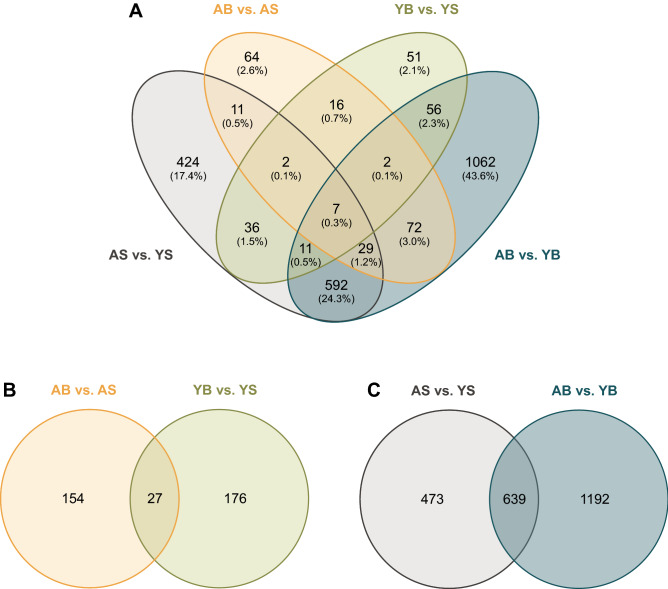
Venn diagram demonstrates the number of overlapped DEGs across burn injury and ages. (**A**) Comparisons between four groups, labeled as a young sham (YS), young burn (YB), adult sham (AS), and adult burn (AB). Four comparisons were colored in yellow (AB vs. AS), green (YB vs. YS), gray (AS vs. YS), and blue (AB vs. YB); (**B**) comparison between young burn and adult burn; (**C**) comparison between burn and sham.

As illustrated in Table 1, 5.09% of muscle mRNA transcripts were differentially expressed between young sham and adult sham animals. Less than 1% of mRNAs were differentially expressed after burn in both age groups; 0.93% when comparing adult burn to sham and 0.83% when comparing young burn to sham. In all comparisons, most changes in mRNA transcripts had fold changes less than 1. Though 22 more mRNAs were expressed in adult burns than young burns, after comparing to each respective sham group, the number of differentially expressed mRNA with absolute fold changes > 1 was greater in the young burn animals. The overlap between differentially expressed genes (DEGs) across burn injury and ages was demonstrated in the Venn diagram (Fig. 1).

**Table 1:** The number of differently expressed mRNA and miRNAs in mouse gastrocnemius between young sham (YS), young burn (YB), adult sham (AS), and adult burn (AB) groups.

Groups	Altered mRNAs				Altered miRNAs			
	YS versus AS	AB versus AS	YB versus YS	YB versus AB	YS versus AS	AB versus AS	YB versus YS	YB versus AB
**Fold changes**								
> − 5 to − 4	0	0	0	0	0	0	0	0
> − 4 to − 3	3	0	0	4	1	0	0	0
> − 3 to − 2	8	0	2	16	1	0	0	2
> − 2 to − 1	98	13	10	125	16	0	0	16
> − 1 to 0	553	62	62	844	52	1	0	50
> 0 to 1	328	112	72	537	16	0	1	21
> 1 to 2	103	14	31	259	33	0	0	24
> 2 to 3	12	1	2	41	26	0	0	28
> 3 to 4	5	1	2	3	6	0	0	3
> 4 to 5	2	0	0	2	3	0	0	3
Down-regulated	662	75	74	989	70	1	0	68
Up-regulated	450	128	107	842	84	0	1	79
Total	1112	203	181	1831	154	1	1	147
%	5.09	0.93	0.83	8.38	6.99	0.05	0.05	6.68

21,859 of protein-coding (39.45%) and 2202 miRNA counts (3.97%) were detected from a total of 55,401 grand counts.

We found 154 miRNAs differentially expressed in young sham animals compared to adult sham mice while 147 miRNAs were differentially expressed when comparing young burns to adult burn animals (Table 1). Similar to mRNA, most miRNAs showed fold changes of less than 1. Mir-1930, mir-29a, mir-29b, and mir-29c were the most down-regulated (fold changes between − 1.7 and − 3.5), while mir-546, mir-3099, and mir-758 were the most up-regulated (fold changes between 3 and 5) in young sham when comparing to adult sham. Interestingly, only two miRNAs were differentially expressed following burn in both the young and adult mice cohorts at 24 h after injury; Mir-10a showed 0.33 fold downregulation in adult burned compared to adult sham mice, and mir-126a showed 0.30 fold upregulation in the young burn compared to young sham mice. All differentially expressed genes and miRNAs are listed in Supplement Table 1A,B.

### Hierarchical clustering of the burn effect on gene expression in adult and young mice after exclusion of basal differences

**Figure 2:**
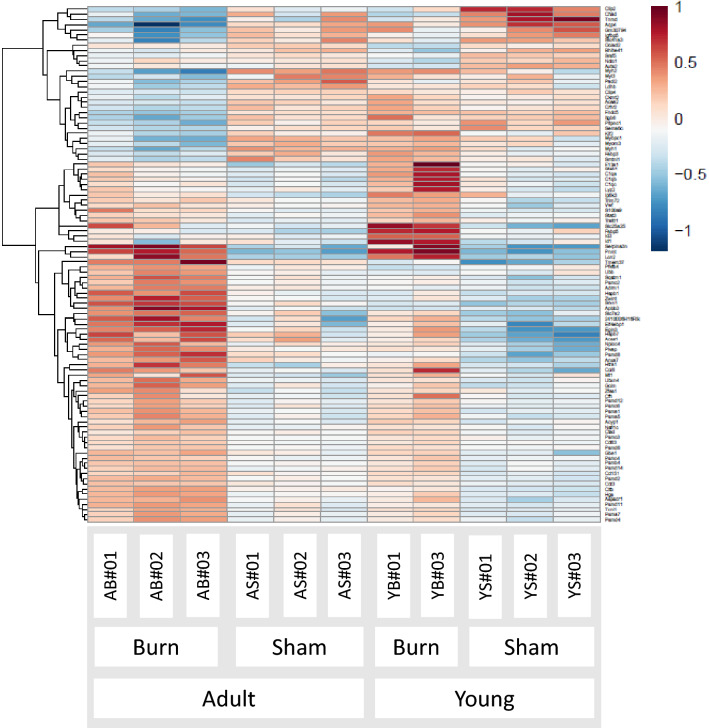
Using analysis of variance, the burn effect difference between young and adult was presented in the clustered heatmap. The heatmap was created with the ‘pheatmap’ R package (Pretty Heatmaps, version: 1.0.12, CRAN) showing the top 100 genes with the largest burn effect differences between the young and adult mice. Four groups were labeled as a young sham (YS), young burn (YB), adult sham (AS), and adult burn (AB). One animal presented as an outlier and thus been excluded in the young burn group. A positive log2 fold change means the change due to the adult burn is higher than the young burn with pseudo red color. The intensity of pseudo colors indicates the degree of an altered gene either upregulated with red color or downregulated with blue color. Two-way ANOVA with a post-hoc test was applied, and significance was accepted at *p* values < 0.05.

Since the basal gene expression profile was different between young and adult sham groups, we evaluated the differential effects of burn on the young versus the adult groups using the likelihood ratio test implemented in DESeq2. Therefore, we compared a reduced model with age as a factor to the full model with the age and burn status. We found 415 genes significantly changed as presented in the heat map illustrating the hierarchical clustering (Fig. 2). A positive log2 fold change with the red pseudo color means the change of the individual gene due to the adult burn is higher than the young burn, and the blue color indicates the opposite. Generally, genes expressing muscle-specific protein (*Myh2, Myh1, Myom3, Mybpc1*) were downregulated in adult burn but not in the young burn group. Genes involved in proteolysis (*Rnf115, Ubb*) and metabolic processes (*Gpt2, Pfkfb4*) were relatively upregulated in adult burn animals.

### GSEA analysis with Hallmark gene sets

Hallmark gene sets are coherently expressed signatures derived by aggregating many MSigDB gene sets to represent well-defined biological states or processes. We analyzed 50 Hallmark gene sets that included 40,071 genes, and found 1,106 differentially expressed genes with 38 overlapped gene sets in young sham compared to adult sham mice. This demonstrates an age effect on the expression of 2.76% of Hallmark gene sets. 21 and 20 gene sets (with 204 and 179 genes each) were affected by the burn in adult and young mice, respectively (Supplemental Table 2A).

Myogenesis, epithelial-mesenchymal transition (EMT), and adipogenesis were the top 3 modified gene sets in young sham mice compared to their adult counterparts. mTORC1 signaling, MYC targets v1, and myogenesis were the top 3 gene-sets affected by burn in the adult mice. Complement, KRAS signaling, and epithelial to mesenchymal transition (EMT) were the top 3 gene sets affected in young mice after burn. The top 25 hallmark gene sets altered in each comparison group with the number of changed genes are listed in Table 2.

**Table 2:** Hallmark gene sets analysis in mouse gastrocnemius between young sham (YS), young burn (YB), adult sham (AS), and adult burn (AB) groups.

Order	Age effect (YS vs. AS)		Burn in adults (AB vs. AS)		Burn in youngs (YB vs. YS)		Burn with age (YB vs. AB)	
	Pathway	N	Pathway	N	Pathway	N	Pathway	N
#01	MYOGENESIS	64	MTORC1_SIGNALING	17	COMPLEMENT	10	MYOGENESIS	74
#02	EPITHELIAL_MESENCHYMAL_TRANSITION	46	MYC_TARGETS_V1	15	KRAS_SIGNALING_UP	10	EPITHELIAL_MESENCHYMAL_TRANSITION	63
#03	ADIPOGENESIS	37	MYOGENESIS	15	EPITHELIAL_MESENCHYMAL_TRANSITION	9	UV_RESPONSE_DN	39
#04	MTORC1_SIGNALING	36	ADIPOGENESIS	12	MTORC1_SIGNALING	9	MYC_TARGETS_V1	45
#05	HYPOXIA	30	REACTIVE_OXYGEN_SPECIES_PATHWAY	6	MYOGENESIS	9	MTORC1_SIGNALING	44
#06	UV_RESPONSE_DN	24	KRAS_SIGNALING_UP	8	COAGULATION	7	ADIPOGENESIS	40
#07	OXIDATIVE_PHOSPHORYLATION	28	P53_PATHWAY	7	APOPTOSIS	7	APICAL_JUNCTION	37
#08	FATTY_ACID_METABOLISM	23	ESTROGEN_RESPONSE_EARLY	6	ADIPOGENESIS	7	HYPOXIA	37
#09	GLYCOLYSIS	25	ESTROGEN_RESPONSE_LATE	6	GLYCOLYSIS	7	UNFOLDED_PROTEIN_RESPONSE	27
#10	APICAL_JUNCTION	23	UV_RESPONSE_UP	5	INTERFERON_GAMMA_RESPONSE	7	COMPLEMENT	36
#11	P53_PATHWAY	23	APOPTOSIS	5	MYC_TARGETS_V1	7	ESTROGEN_RESPONSE_LATE	35
#12	MITOTIC_SPINDLE	22	ANDROGEN_RESPONSE	4	UV_RESPONSE_UP	6	INTERFERON_ALPHA_RESPONSE	24
#13	ESTROGEN_RESPONSE_EARLY	21	BILE_ACID_METABOLISM	4	HYPOXIA	6	MITOTIC_SPINDLE	34
#14	HEME_METABOLISM	21	GLYCOLYSIS [200]	5	ALLOGRAFT_REJECTION	5	KRAS_SIGNALING_UP	34
#15	UNFOLDED_PROTEIN_RESPONSE	15	OXIDATIVE_PHOSPHORYLATION	5	APICAL_JUNCTION	5	P53_PATHWAY	34
#16	TNFA_SIGNALING_VIA_NFKB	20	PEROXISOME	3	TNFA_SIGNALING_VIA_NFKB	5	ESTROGEN_RESPONSE_EARLY	33
#17	UV_RESPONSE_UP	17	HYPOXIA	4	FATTY_ACID_METABOLISM	4	TNFA_SIGNALING_VIA_NFKB	33
#18	G2M_CHECKPOINT	19	INTERFERON_GAMMA_RESPONSE	4	INTERFERON_ALPHA_RESPONSE	3	GLYCOLYSIS	32
#19	XENOBIOTIC_METABOLISM	19	TNFA_SIGNALING_VIA_NFKB	4	INFLAMMATORY_RESPONSE	4	HEME_METABOLISM	32
#20	APOPTOSIS	15	XENOBIOTIC_METABOLISM	4	P53_PATHWAY	4	INTERFERON_GAMMA_RESPONSE	32
#21	MYC_TARGETS_V1	17	UNFOLDED_PROTEIN_RESPONSE	3			ANDROGEN_RESPONSE	22
#22	REACTIVE_OXYGEN_SPECIES_PATHWWAY	8					UV_RESPONSE_UP	28
#23	ANDROGEN_RESPONSE	11					APOPTOSIS	28
#24	IL2_STAT5_SIGNALING	16					MYC_TARGETS_V2	16
#25	PEROXISOME [104]	11					IL2_STAT5_SIGNALING	30
#26	ESTROGEN_RESPONSE_LATE	16					G2M_CHECKPOINT	30
#27	PI3K_AKT_MTOR_SIGNALING	11					XENOBIOTIC_METABOLISM	30
#28	MYC_TARGETS_V2	8					COAGULATION	24
#29	BILE_ACID_METABOLISM	11					OXIDATIVE_PHOSPHORYLATION	29
#30	KRAS_SIGNALING_UP	15					FATTY_ACID_METABOLISM	25
#31	TGF_BETA_SIGNALING	7					E2F_TARGETS	27
#32	PROTEIN_SECRETION	9					INFLAMMATORY_RESPONSE	27
#33	COMPLEMENT	13					TGF_BETA_SIGNALING	13
#34	KRAS_SIGNALING_DN	13					CHOLESTEROL_HOMEOSTASIS	15
#35	COAGULATION	10					PROTEIN_SECRETION	17
#36	NOTCH_SIGNALING	4					BILE_ACID_METABOLISM	18
#37	CHOLESTEROL_HOMEOSTASIS	6					PI3K_AKT_MTOR_SIGNALING	17
#38	ANGIOGENESIS	4					PEROXISOME	16
#39							REACTIVE_OXYGEN_SPECIES_PATHWAY	10
#40							ANGIOGENESIS	8
#41							ALLOGRAFT_REJECTION	21
#42							DNA_REPAIR	17
#43							IL6_JAK_STAT3_SIGNALING	11
#44							APICAL_SURFACE	7
#45							HEDGEHOG_SIGNALING	6

Hallmark gene sets summarize 50 specific well-defined biological processes, which were generated by a computational methodology based on identifying overlaps between gene sets in other MSigDB collections and retaining genes that display coordinate expression.

N, The number of altered genes.

### Canonical Wiki-Pathways

Canonical Pathways gene sets analysis is derived from the WikiPathways pathway database. Using the WikiPathways database, we identified over 100 out of 587 overlapping gene sets in young sham compared to adult sham mice. Only 24 and 15 overlapping gene sets were identified when comparing adult burn and young burn mice to their sham counterparts, respectively (Supplemental Table 2B). VEGFA-VEGFR2, focal adhesion, and nuclear receptors meta-pathways were the top 3 altered gene sets when analyzing age effect by comparing young to adult sham animals. Proteasome degradation, parkin-ubiquitin-proteasomal system, and nuclear receptors meta-pathways are the top 3 gene sets altered by the burn in the adult burn while the proteasome degradation, focal adhesion, and PI3K-AKT pathways are the top 3 affected by burn in young animals. These data confirm the role of the ubiquitin–proteasome pathway in regulating the rapid muscle proteolysis starting at the 24–48 h ebb phase after burn. The top 25 canonical pathways gene sets with the number of genes altered in each pathway are listed in Table 3.

**Table 3:** Wiki gene sets analysis in mouse gastrocnemius between young sham (YS), young burn (YB), adult sham (AS), and adult burn (AB) groups. N: the number of altered genes.

Order	Age effect (YS vs. AS)		Burn in adults (AB vs. AS)		Burn in youngs (YB vs. YS)		Burn with age (YB vs. AB)	
	Pathway	N	Pathway	N	Pathway	N	Pathway	N
#01	VEGFAVEGFR2_SIGNALING	64	PROTEASOME_DEGRADATION	26	PROTEASOME_DEGRADATION	10	VEGFAVEGFR2_SIGNALING	89
#02	FOCAL_ADHESIONPI3KAKTMTOR	38	PARKINUBIQUITIN_PROTEASOMAL_SYSTEM	18	PARKINUBIQUITIN_PROTEASOMAL_SYSTEM	6	FOCAL_ADHESION_PI3K_AKT_MTOR	58
#03	FOCAL_ADHESION	28	NUCLEAR_RECEPTORS_METAPATHWAY	12	MICROGLIA_PATHOGEN_PHAGOCYTOSIS	5	MYOMETRIAL_RELAXATION_AND_CONTRACTION	40
#04	ADIPOGENESIS	22	SELENIUM_MICRONUTRIENT_NETWORK	6	COMPLEMENT_AND_COAGULATION_CASCADE	5	FOCAL_ADHESION [202]	43
#05	INSULIN_SIGNALING	23	ONE_CARBON_METABOLISM	5	STRIATED_MUSCLE_CONTRACTION_PATHWAY	4	IL18_SIGNALING_PATHWAY	50
#06	NUCLEAR_RECEPTORS_METAPATHWAY	32	EXTRACELLULAR_VESICLES_IN_THE_CROSSTALK_OF_CARDIAC_CELLS	4	PATHWAYS_IN_CLEAR_CELL_RENAL_CELL_CARCINOMA	5	INSULIN_SIGNALING	35
#07	NONALCOHOLIC_FATTY_LIVER_DISEASE	22	VEGFAVEGFR2_SIGNALING	11	FOCAL_ADHESION_PI3K_AKT_MTOR	8	STRIATED_MUSCLE_CONTRACTION	17
#08	ANGIOPOIETIN_LIKE_PROTEIN_8_REGULATLATORY_PATHWAY	20	MYOMETRIAL_RELAXATION_AND_CONTRACTION	7	COMPLEMENT_ACTIVATION	3	PI3K_AKT_SIGNALING_PATHWAY	50
#09	IL18_SIGNALING	29	FACTORS_AND_PATHWAYS_AFFECTING_INSULINLIKE_GROWTH_FACTOR_IGF1AKT	4	PI3KAKT_SIGNALING	8	SENESCENCE_AND_AUTOPHAGY_IN_CANCER	26
#10	MAPK_SIGNALING_PATHWAY	27	NRF2_PATHWAY	6	TYROBP_CAUSAL_NETWORK	4	ANGIOPOIETIN_LIKE_PROTEIN_8_REGULATORY	28
#11	PI3KAKT_SIGNALING_PATHWAY	31	AMINO_ACID_METABOLISM	5	FOCAL_ADHESION	6	EGFEGFR_SIGNALING_PATHWAY [163]	31
#12	TYPE_I_COLLAGEN_SYNTHESIS_IN_THE_CONTEXT_OF_OSTEOGENESIS_IMPERFECTA	10	CILIARY_LANDSCAPE	7	CILIARY_LANDSCAPE	6	MAPK_SIGNALING_PATHWAY	39
#13	MYOMETRIAL_RELAXATION_AND_CONTRACTION	20	EGFEGFR_SIGNALING_PATHWAY	6	MACROPHAGE_MARKERS	2	MIRNA_TARGETS_IN_ECM_AND_MEMBRANE_RECEPTORS	16
#14	EGFEGFR_SIGNALING_PATHWAY [163]	19	INTRACELLULAR_TRAFFICKING_PROTEINS_INVOLVED_IN_CMT_NEUROPATHY	3	GLYCOGEN_SYNTHESIS_AND_DEGRADATION	3	ADIPOGENESIS	27
#15	MIRNA_TARGETS_IN_ECM_AND_MEMBRANE_RECEPTORS	10	ADIPOGENESIS	5	OXIDATIVE_DAMAGE	3	CIRCADIAN_RHYTHM_RELATED_GENES	33
#16	SENESCENCE_AND_AUTOPHAGY_IN_CANCER	15	FOCAL_ADHESION [	6			EBOLA_VIRUS_PATHWAY_ON_HOST	25
#17	AMPACTIVATED_PROTEIN_KINASE_AMPK	12	TYPE_I_COLLAGEN_SYNTHESIS_IN_THE_CONTEXT_OF_OSTEOGENESIS_IMPERFECTA	3			TGFBETA_SIGNALING_PATHWAY	25
#18	SMALL_CELL_LUNG_CANCER	14	FATTY_ACID_BETA_OXIDATION	3			EPITHELIAL_TO_MESENCHYMAL_TRANSITION_IN_COLORECTAL_CANCER	27
#19	CALCIUM_REGULATION_IN_THE_CARDIAC_CELL	17	OXIDATIVE_STRESS	3			HEPATITIS_B_INFECTION	26
#20	AMINO_ACID_METABOLISM	13	STRIATED_MUSCLE_CONTRACTION	3			CHEMOKINE_SIGNALING_PATHWAY	27
#21	GLYCOLYSIS_AND_GLUCONEOGENESIS	9	FOCAL_ADHESION_PI3K_AKT_MTOR	7			APOPTOSISRELATED_NETWORK_DUE_TO_ALTERED_NOTCH3_IN_OVARIAN_CANCER	15
#22	PROLACTIN_SIGNALING	11	RENIN_ANGIOTENSIN_ALDOSTERONE_SYSTEM_RAAS	3			NONGENOMIC_ACTIONS_OF_125_DIHYDROXYVITAMIN_D3	17
#23	STRIATED_MUSCLE_CONTRACTION	8	ENDOPLASMIC_RETICULUM_STRESS_RESPONSE_IN_CORONAVIRUS_INFECTION	3			EXERCISEINDUCED_CIRCADIAN_REGULATION	14
#24	SEROTONIN_RECEPTOR_2_AND_ELKSRFGATA4	6	TRANSCRIPTIONAL_CASCADE_REGULATING_ADIPOGENESIS	2			RAS_SIGNALING [185]	28
#25	NRF2_PATHWAY	15					CALCIUM_REGULATION_IN_THE_CARDIAC_CELL	25
#26	PATHWAYS_AFFECTED_IN_ADENOID_CYSTIC_CARCINOMA	10					NUCLEAR_RECEPTORS_METAPATHWAY	39
#27	PPAR_SIGNALING	10					PATHWAYS_IN_CLEAR_CELL_RENAL_CELL_CARCINOMA	18
#28	TRANSCRIPTION_FACTOR_REGULATION_IN_ADIPOGENESIS	6					GLYCOGEN_SYNTHESIS_AND_DEGRADATION [40]	12
#29	MET_IN_TYPE_1_PAPILLARY_RENAL_CELL_CARCINOMA	9					BRAINDERIVED_NEUROTROPHIC_FACTOR_BDNF_SIGNALING_PATHWAY	23
#30	FATTY_ACID_BETA_OXIDATION	7					GASTRIN_SIGNALING_PATHWAY	20
#31	FRAGILE_X_SYNDROME	13					CILIARY_LANDSCAPE	29
#32	INSULIN_SIGNALLING_IN_HUMAN_ADIPOaCYTES_DIABETIC_CONDITION	4					AMPACTIVATED_PROTEIN_KINASE_AMPK	15
#33	INSULIN_SIGNALLING_IN_HUMAN_ADIPOCYTES_NORMAL_CONDITION	4					REGULATION_OF_ACTIN_CYTOSKELETON	23
#34	ENDOPLASMIC_RETICULUM_STRESS_RESPONSE_IN_CORONAVIRUS_INFECTION	8					SMALL_CELL_LUNG_CANCER	18
#35	ARRHYTHMOGENIC_RIGHT_VENTRICULAR_CARDIOMYOPATHY	10					FRAGILE_X_SYNDROME	20
#36	LEPTIN_SIGNALING_PATHWAY	10					NONSMALL_CELL_LUNG_CANCER	15
#37	BRAINDERIVED_NEUROTROPHIC_FACT	14					PATHOGENIC_ESCHERICHIA_COLI_INFECTION	13
#38	CIRCADIAN_RHYTHM_RELATED_GENES	17					SIGNALING_PATHWAYS_IN_GLIOBLASTOMA	16
#39	MITOCHONDRIAL_LCFATTY_ACID_BETAOXIDATION [17]	5					ENDOCHONDRAL_OSSIFICATION	14
#40	REGULATION_OF_ACTIN_CYTOSKELETON	14					ENDOCHONDRAL_OSSIFICATION_WITH_SKELETAL_DYSPLASIAS	14
#41	GASTRIN_SIGNALING_PATHWAY	12					CARDIAC_HYPERTROPHIC_RESPONSE	13
#42	NETRINUNC5B_SIGNALING	8					TYPE_I_COLLAGEN_SYNTHESIS_IN_THE_CONTEXT_OF_OSTEOGENESIS_IMPERFECTA	10
#43	G13_SIGNALING	7					G_PROTEIN_SIGNALING_PATHWAYS	17
#44	INTERFERON_TYPE_I_SIGNALING	8					THERMOGENESIS	18
#45	GENES_TARGETED_BY_MIRNAS_IN_ADIPOCYTES	5					TNF_RELATED_WEAK_INDUCER_OF_APOPTOSIS_TWEAK_SIGNALING_PATHWAY	11
#46	TNF_RELATED_WEAK_INDUCER_OF_APOPTOSIS_TWEAK_SIGNALING	7					RAC1PAK1P38MMP2_PATHWAY	14
#47	PATHWAYS_IN_CLEAR_CELL_RENAL_CELL_CARCINOMA	10					CELL_CYCLE	19
#48	INTEGRINMEDIATED_CELL_ADHESION	11					TRANSLATION_FACTORS	12
#49	ELECTRON_TRANSPORT_CHAIN_OXPHOS_SYSTEM_IN_MITOCHONDRIA	11					WNT_SIGNALING_PATHWAY_NETPATH	12
#50	STEROL_REGULATORY_ELEMENTBINDING_PROTEINS_SREBP	9					PDGFRBETA_PATHWAY	9

### miRNA target prediction gene sets

To determine the interaction of miRNA and gene expression, we chose all miRNA target prediction gene sets from the combined superset of both the online database for miRNA target prediction and functional annotations (miRDB) and legacy sets in Gene Set Enrichment Analysis (GESA). Over 100 of 2,598 miRNAs were altered in the young sham compared to their adult counterparts (Supplemental Table 2C). Similar to mRNA data, a smaller number of miRNA were affected by burn; 30 and 14 miRNAs were identified in adult and young burned animals, respectively (Table 4). MIR-12316, MIR153-5P, and MIR8485 are the top 3 miRNAs related to the number of mRNAs differing in adult sham animals compared to the young ones. MIR124-3P, MIR506-3P, and MIR520D-5P are the top 3 miRNAs related to genes affected by burn in adult animals. MIR6867-5P, MIR3658, and MIR124-3P are the top 3 miRNAs regulating mRNA altered by the burn in young animals. These data confirm the specificity of miRNAs in regulating a large number of genes in response to both age and injury. All miRNA target prediction gene sets are listed in Table 4.

**Table 4:** miRNA interactions analysis in mouse gastrocnemius between young sham (YS), young burn (YB), adult sham (AS), and adult burn (AB) groups.

Order	Age effect (YS vs. AS)		Burn in adults (AB vs. AS)		Burn in youngs (YB vs. YS)		Burn with age (YB vs. AB)	
	miRNAs	N	miRNAs	N	miRNAs	N	miRNAs	N
#01	MIR12136	90	MIR124_3P	15	MIR6867_5P	15	MIR12136	137
#02	MIR153_5P	83	MIR506_3P	15	MIR3658	13	MIR524_5P	131
#03	MIR8485	79	MIR520D_5P	15	MIR124_3P	12	MIR520D_5P	130
#04	MIR548AJ_3P_MIR548X_3P	76	MIR524_5P	15	MIR506_3P	12	MIR153_5P	130
#05	MIR651_3P	75	MIR607	14	AATGTGA_MIR23A_MIR23B	10	MIR8485	129
#06	MIR3658	74	TGCTGCT_MIR15A_MIR16_MIR15B_MIR195_MIR424_MIR497	13	MIR589_3P	8	MIR548AJ_3P_MIR548X_3P	118
#07	MIR524_5P	74	GTGCCTT_MIR506	13	MIR16_2_3P	7	MIR3662	118
#08	MIR335_3P	72	MIR26A_5P	12	MIR195_3P	7	MIR548E_5P	113
#09	MIR1277_5P	72	MIR26B_5P	12	MIR3934_3P	6	MIR506_3P	113
#10	MIR548AH_3P_MIR548AM_3P	69	MIR1297	12	MIR6509_5P	5	MIR124_3P	112
#11	MIR19A_3P	68	MIR95_5P	12	MIR7155_5P	5	MIR335_3P	109
#12	MIR19B_3P	68	MIR4465	12	MIR323A_5P	4	MIR19B_3P	108
#13	MIR548J_3P	68	MIR4698	11	MIR6876_3P	4	GTGCCTT_MIR506	108
#14	MIR548AE_3P_MIR548AQ_3P	68	MIR7_2_3P	11	MIR626	4	MIR19A_3P	108
#15	MIR570_3P	67	MIR7_1_3P	11			MIR3658	107
#16	TGCTGCT_MIR15A_MIR16_MIR15B_MIR195_MIR424_MIR497	66	MIR7106_5P	10			MIR570_3P	106
#17	MIR1297	66	TACTTGA_MIR26A_MIR26B	9			MIR651_3P	106
#18	MIR4698	65	TCATCTC_MIR143	7			MIR95_5P	104
#19	MIR548E_5P	65	MIR1249_5P	7			LET_7A_3P	103
#20	MIR95_5P	64	MIR6797_5P	7			LET_7B_3P	102
#21	GTGCCTT_MIR506	64	AAGCAAT_MIR137	7			LET_7F_1_3P	102
#22	TTTGCAC_MIR19A_MIR19B	61	ATGTTAA_MIR302C	7			MIR98_3P	102
#23	TGGTGCT_MIR29A_MIR29B_MIR29C	61	MIR646	7			MIR548AH_3P_MIR548AM_3P	102
#24	MIR27A_3P_MIR27B_3P	61	MIR5787	7			MIR548J_3P	102
#25	MIR9985	61	MIR4505	7			MIR548AE_3P_MIR548AQ_3P	102
#26	MIR1468_3P	61	GCAAAAA_MIR129	6			MIR4659A_3P_MIR4659B_3P	100
#27	TGCCTTA_MIR124A	60	MIR623	5			MIR1297	97
#28	MIR144_3P	59	MIR449B_3P	5			MIR10527_5P	95
#29	LET_7C_3P	59	MIR143_5P	5			MIR1468_3P	95
#30	MIR23C [594]	59	MIR1224_3P	4			TGGTGCT_MIR29A_MIR29B_MIR29C	93
#31	MIR182_5P	58					MIR548AC	89
#32	MIR23A_3P_MIR23B_3P	58					MIR548H_3P_MIR548Z	89
#33	MIR15B_5P	56					MIR9985	88
#34	MIR16_5P	56					TTTGCAC_MIR19A_MIR19B	87
#35	MIR195_5P	56					MIR182_5P	87
#36	MIR15A_5P	56					MIR23A_3P_MIR23B_3P	87
#37	MIR10527_5P	56					MIR23C	87
#38	AATGTGA_MIR23A_MIR23B	55					MIR548BB_3P	87
#39	MIR29A_3P	55					MIR548D_3P	87
#40	MIR29B_3P_MIR29C_3P[434]	55					MIR27A_3P_MIR27B_3P	87
#41	MIR137_3P	55					MIR4666A_3P	86
#42	ACCAAAG_MIR9	54					TGCCTTA_MIR124A	84
#43	MIR3133	53					MIR4728_5P	84
#44	CAGTATT_MIR200B_MIR200C_MIR429	52					MIR29A_3P	81
#45	ACTGTGA_MIR27A_MIR27B	52					MIR29B_3P_MIR29C_3P	81
#46	TGAATGT_MIR181A_MIR181B_MIR181C_MIR181D	52					MIR9_5P	79
#47	MIR497_5P	52					MIR6785_5P	79
#48	MIR9_5P	51					TGAATGT_MIR181A_MIR181B_MIR181C_MIR181D	77
#49	CCTGCTG_MIR214	39					MIR144_3P	77
#50	MIR5682	34					MIR101_3P	74

Gene sets containing high-confidence gene-level predictions of mouse miRNA targets as catalogued by miRDB v6.0 algorithm^18^.

N, The number of altered genes.

## Discussion

This study investigates the acute response of muscle-specific transcriptome to severe scald burn in a mouse model, and further deciphers potential interactions between miRNA and mRNA expression profiles. To assess transcriptomic differences between pediatric/adolescent and adult subjects, we chose to compare young (5-week-old) and adult (11-week-old) mice. We described the characteristic profiles of mRNA and miRNA expression in both young and adult mice and their changes in response to injury using an NGS analysis. These changes affected less than 1% genes and 0.05% miRNA covered by the 21,859 mouse GRCm38 genome library and 2202 miRNA database respectively, suggesting that miRNA possesses wide substrate specificity when regulating genes pathways involved in muscle homeostasis regardless of age.

GESA analysis confirmed gene pathways that are differentially expressed in response to both age and burn. We used two different sets of GSEA to identify the pattern of gene pathways in response to age, and burn and found compatible data. We also sorted DEGs by either up-regulated or down-regulated and further clarified the activities of biological processing and signal pathway. BPs including skeletal system development and regulation of cell differentiation were activated in young mice compared to adult. Apoptotic process and regulation of cell death were activated in adult burn (comparing to adult sham), while interestingly, inflammatory and immune responses were activated in young burn (comparing to young sham) (Supplemental Table 3A). The results of WikiPathway analysis were also specified (Supplemental Table 3B) Focal adhesion was activated and oxidative stress pathway was deactivated in young sham when comparing adult sham. Proteasome degradation was activated in both young and adult following injury. Oxidative stress response was more presented in adult burn, while inflammatory response like complement activation and macrophage markers was more activated in young burn comparing to young sham. Although major catabolic gene responses to burn were observed in both young and adult animals, additional specific pathway alterations were distinguishable between young and adult mice. These findings may reflect differential effects observed in young adolescents compared to adult burn patients.

mRNA transcript profiles in the muscle, in response to severe injury, were age-specific. Transcriptome analysis of coagulation factors in a human closely related species such as the Chinese rhesus macaque showed that basal gene expression was affected by age^19^. Changes in the expression of muscle growth genes were also reported in bulls between 15 and 19 months of age^20^. In our study, a comparison of muscle-specific transcriptome profiles revealed an age effect on gene expression between 5-week-old and 11 week-old mice with paralleled burn effects on gene expression in both young and adult groups. The protein degradation pathway was the most affected gene pathway in both adult and young burned mice. Ubiquitin expression were shown to up-regulate in both human and rat skeletal muscles during ageing^21^. However, detailed information regarding specific genes, transcriptome fold changes, and the number of altered transcripts were different, which indicates a distinguished response to injury in young mice compared to adults. One example is gene expression regulated by polyubiquitination. *Ubb* gene encodes ubiquitin and was upregulated 0.64-fold in the adult burn group when comparing adult sham, but it was not differentially expressed in the young burn group. However, the *Ubc* gene encoding Polyubiquitin-C to maintain cellular ubiquitin level, was down-regulated (-1.72-fold) in the young burn group. A direct comparison between the young burned and the adult burned mice shows that *Ubb* gene level increases 0.81-fold and the *Ubc* gene level increases 1.13, further indicating a delicate tuning on the regulatory mechanism with different ages at the transcriptional level.

Myogenesis was another typical altered GO-BP pathway in the study. In this study, we observed the most number of DEGs enriched in myogenesis when comparing young sham to adult sham. The previous study showed that the progenitor cell activity in aged muscle can be restored when treated with young serum^22^. It is not surprise that the myogenic activity is greater in younger mice. Further stratification of up- and down-regulated genes, we confirmed that activated biological processing of skeletal muscle development and cell differentiation in young sham group. In this study we also noticed that myogenesis has also been affected by external stimulations. In a previous study of burn mouse model, we found that severe burn caused insufficient myogenic activation^23^. Corrick et al. set up an in vitro model with human serum stimulation from burn patients, and they explored that myogenesis impairment could be related to STAT pathway activation^24^. Further dissection of specific DEGs will help us understand better the role of myogenesis in the maintenance of muscle homeostasis in young burn victims. miRNAs serve as negative regulators of gene expression. Similar to mRNA, age is also prominent in miRNA expression changes after injury, and the miRNA profile presented with different characters of neurobiological development with age^25^. Using microarray and TaqMan-based expression analysis of miRBase 10.0, Moreau et al. observed that 312 miRNAs were differentially expressed in 48 post-mortem human tissue of fetal, young, and adult brains^25^. In our study, we observed increased expression of the miRNA 29s family with age. The miRNA 29s family includes three mature members a, b, c, that regulate different genes involved in cell growth, differentiation, apoptosis, and regulation of the immune response^26^. miR-29s family members target at least 16 extracellular matrix genes for focal adhesion, and are upregulated in aged mice aortas^27^. For muscle tissue, miR-29 impairs myoblast proliferation, to assist with cell arrest protein expression (p53, p16, and pRB) during aging^28^. We found that the endogenous levels of miR-29 are elevated with age, specifically in the C57BL/6 male mouse gastrocnemius 5-week-old compared to the 11-week-old. Though further work is indicated to investigate this link, age-related miRNAs could interfere with the genomic response to burn. Therefore, our data show that miR-29 could be an important therapeutic target of aging-related diseases, particularly after injury.

To our surprise, only one miRNA significantly changed in response to burn in each age group, with miR-10a downregulated and miR-126a upregulated in adult and young burned mice respectively. miR-10 is overexpressed following ischemic injury^29^. miR-126 has protective effects in an animal model of endotoxemia-induced vascular injury^30,31^. Using microarray analysis, we previously reported miRNA differential expression 14 days after burn in rats^11^. Hu et al., showed changes in the miRNA profiles over 1 and 7 days following traumatic brain injury, demonstrating one/multiple miRNA expression changes over time^32^. Changes in the genomic kinetics over time have been observed in critical trauma and burn patients^33^, which correspond with a systemic inflammatory^34^, and muscle pathophysiological changes^35^. In a previous study, we examined the muscle transcriptome profile from male adult rats at 14 days while the current study examined early changes in mouse muscle transcriptome profile at 1-day after-burn. Our results suggest that changes in mRNA and miRNA expression reflect a temporal protective feedback response starting 24 h after burn, and that further time course studies are needed to characterize this response.

Using miRNA and mRNA interactive pathway analysis, we found that the number of miRNAs interacting with genes is similar to the number of gene pathways affected in response to age and burn. The number of interactive mRNAs range from 90 to 34 in young mice comparing to adult mice without injury. The number ranges decreased from 15 to 4 in young burned mice or adult burned mice. The highly expressed gene-related miRNA in response to severe burn in both age groups were MIR-506_3p, MIR-124_3p, MIR-6867_5p, and MIR-3658 with the greatest number of related- altered genes in young burned mice. Interestingly, MIR-3658 has 74 altered genes connected in adult sham when compared to young sham. These novel findings provide a new avenue to study the roles in pediatric burns.

In summary, this study demonstrates the characteristic profile of gene expression from skeletal muscle in young and adult burns. Prominent age effects were presented in transcriptional levels with abundant alterations of genes, miRNAs, and interactions, leading to regulation pathways mainly on myogenesis, cell growth, and development. Our results further confirmed the effect of severe burn in mice muscle, with fewer gene sets and miRNAs affected in both age groups. Protein degradation, inflammation, and related pathways are affected by injury in both young and adult groups, however, more genes and related pathways were affected in the adults. The study descripted the different responses at the transcriptional level, including the altered DEGs and miRNA among the experimental groups, followed by an analysis of gene sets related pathways and interactions of miRNAs to mRNAs by GESA tool. Further work in correlation of muscle pathophysiological changes could be conducted from both animal model and clinical research.

This study also provides information on the miRNA and mRNA expression profiles in young burn mice and could lead to further studies on the mechanisms of gene regulation in pediatric burn patients. As for pediatric burn research, most metabolic parameters were studied broadly, however, genomic information reflecting those changes is missing. The current study opens a direction that emphasizes that age influences metabolic changes in with development and aging after burn. The pathophysiologic response evaluation combined with genomic profile examination will allow for a better picture of events, and explain variation in results greater than one single layer observation at the transcriptional level.

## Methods

Twelve C57BL6 male mice (six 4-weeks-old, young; and six 10-weeks-old, adult) were obtained from Charles River Laboratory (Wilmington, MA). All procedures followed the NIH and ARRIVE guidelines and were approved by the local IACUC at the University of Texas Medical Branch at Galveston (UTMB). Animals were housed in a temperature-controlled room with a 12-h light/ dark cycle with free access to food and water.

After one-week acclimation, half of the young and adult animals received a 25% body surface area (TBSA) scald burn^23^. Briefly, mice were anesthetized with 2% isoflurane inhalation and shaved on the back and belly, 30 min after receiving a subcutaneous injection of 0.05 mg/kg buprenorphine. Mice were placed in a mold with an opening to expose about 12.5% body surface after receiving 1 mL of 0.9% saline injection subcutaneously along the spinal column. Mice were then immersed in 98 °C water for 10 s on the dorsal and 2 s on the ventral side to receive a total 25% TBSA full-thickness scald burn. The animals received 1.5 mL of intraperitoneal lactated Ringer’s solution for resuscitation during the burn procedure. Sham animals received the same procedure with the anesthetics and analgesics but did not receive a scald burn or resuscitation. Animals all survived after burn procedure, and which be consistently observed in our previous studies^23,36^. Animals were euthanized 24 h after burn with a CO_2_ inhalation overdose. Muscle tissues were harvested and stored at − 80 °C for further analysis.

**Figure 3:**
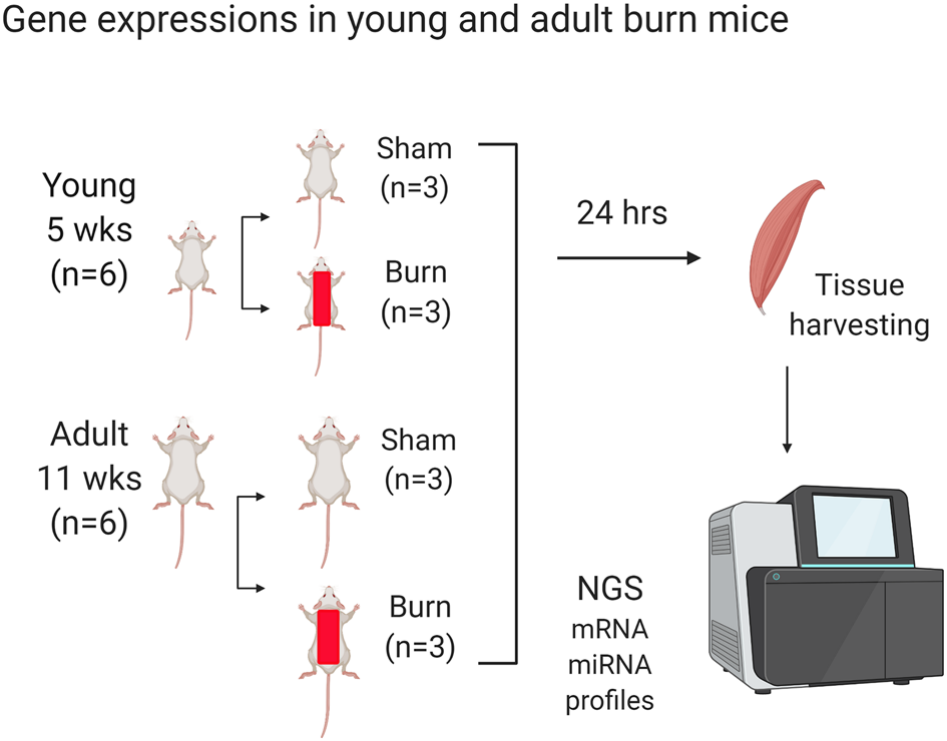
Experiment flow chart outlines the study design and sample processing. The Image was created in BioRender.

Severe burn caused metabolic changes include the early ebb stress phase and later flow phase. The transition time point is usually 24-48 h after injury^37^. Therefore, we chose the initial time point 24 h to explore the regulatory mechanism of post-burn muscle wasting in transcriptional level. Gastrocnemius tissue contains mixed slow-and fast- myofiber types, and its anatomic location is far away from burn wound site in mouse trunk. We and others analyzed the gastrocnemius tissue to represent the muscle response to systemic influence in mouse and rat models^38,39^. Gastrocnemius tissues (~ 25 mg) were processed for total RNA extraction using Qiagen RNeasy Mini Kit (Germantown, MD). After measurement of concentration with Nanodrop 2000/2000c Spectrophotometers (ThermoFisher, Waltham, MA), 2 µg of total RNA were analyzed at the Next Generation Sequence (NGS) core laboratory at UTMB. (Supplemental file 4) RNA sample quality was confirmed using an Agilent Bioanalyzer 2100 (Agilent Technologies, Santa Clara, CA). Poly-adenylated RNA (mRNA) and microRNA (miRNA) sequence libraries were prepared with NEBNext Ultra II RNA and Small RNA kits, respectively (New England BioLabs, Ipswich, MA). Libraries were sequenced on an Illumina NextSeq 550, using the mid-output kit and paired-end 75 base parameters (Fig. 3).

Reads from the mRNA samples were mapped to the mouse GRCm38 genome reference using the STAR read aligner, with the quantMode GeneCounts option to quantify read counts based on the Gencode version M25 mouse annotation. Reads from the miRNA samples were trimmed of adapters using the mirDeep2.0 software package, then mapped to the mouse genome reference with STAR. The featureCounts program in the subread software package was used to count reads mapping to miRNAs. Differential gene expression for mRNAs and miRNAs was estimated with the Differential gene expression analysis based on the negative binomial distribution (DESeq2) package following the authors’ vignette. The significance limit was set to an adjusted *p* value of < 0.05. Descriptive statistics was applied for transcript profile characterization. Data replicability was demonstrated in the PCA plot (Supplemental Fig. 1) Gene expression pathway and miRNA interaction were further analyzed using an online program, Gene Set Enrichment Analysis (GSEA) (www.gsea-msigdb.org). Two-way ANOVA with a post-hoc test was applied, and significance was accepted at *p* values < 0.05.

### Conference presentation

Data was presented at the 44th Shock annual meeting, virtual on October 10–14, 2021.

## Supplementary Information

Supplementary Information 1.

Supplementary Information 2.

Supplementary Information 3.

Supplementary Information 4.

Supplementary Information 5.

## Data Availability

All raw transcripts data has been submitted to the NCBI GEO online repository at July 19, 2022—[geo] GEO Submission (GSE208548). The accessible link is as following: https://www.ncbi.nlm.nih.gov/geo/query/acc.cgi?acc=GSE208548. The data has been released at November 4, 2022.
