# Supplementary material for: Skeletal muscle transcriptome is affected by age in severely burned mice
Source: Sci Rep. 2022 Dec 14;12:21584. doi: 10.1038/s41598-022-26040-1 (PMC9748408; doi:10.1038/s41598-022-26040-1)
Supplement: Supplementary file 1 — Supplementary Information 1. [file 41598_2022_26040_MOESM1_ESM.docx]

| **A**  **Supplement Table 1** All differentially expressed **(A) mRNAs (DEGs)** and **(B) miRNAs** (Padj ≤0.05) with fold changes in mouse gastrocnemius between young sham (YS), young burn (YB), adult sham (AS), and adult burn (AB) groups. |  |  |  |  |  |  |  |
| --- | --- | --- | --- | --- | --- | --- | --- |
| **Altered protein coding genes** | | |  | **P adj ≤ 0.05** | |  |  |
| **Age effect (YS vs. AS)** | | **Burn in Adults (AB vs. AS)** | | **Burn in Youngs (YB vs. YS)** | | **Burn with age (YB vs. AB)** | |
| **Gene** | **log2FoldChange** | **Gene** | **log2FoldChange** | **Gene** | **log2FoldChange** | **Gene** | **log2FoldChange** |
| Rasd2 | -3.49 | *Aqp4* | -1.92 | *Chad* | -2.10 | Morn4 | -3.56 |
| Jchain | -3.27 | *Myh2* | -1.53 | *Kera* | -2.04 | Cd27 | -3.21 |
| Odf3l2 | -3.15 | *Esrrg* | -1.38 | *Arntl* | -1.83 | Slc15a5 | -3.17 |
| Slc15a5 | -2.84 | *Mfap4* | -1.20 | *Ubc* | -1.72 | 1700001O22Rik | -3.00 |
| 1700001O22Rik | -2.49 | *Padi2* | -1.16 | *Tnmd* | -1.66 | Odf3l2 | -2.99 |
| Musk | -2.44 | *Itgb6* | -1.16 | *Comp* | -1.62 | Rasd2 | -2.91 |
| Irx3 | -2.39 | *Myh1* | -1.13 | *Mettl21c* | -1.46 | Odc1 | -2.78 |
| Actr3b | -2.33 | *Myom3* | -1.09 | *Amd2* | -1.45 | Kcnf1 | -2.66 |
| Samd10 | -2.33 | *Relt* | -1.08 | *Tnc* | -1.40 | Ostn | -2.56 |
| Odc1 | -2.13 | *Mybpc1* | -1.07 | *Cilp2* | -1.30 | Fbxo32 | -2.56 |
| Sbk2 | -2.02 | *Myl3* | -1.04 | *Gdap1* | -1.14 | Cxcl13 | -2.55 |
| Pdpr | -1.98 | *Slc41a3* | -1.02 | *Auts2* | -1.07 | Gadd45g | -2.51 |
| Ostn | -1.94 | *Pde4a* | -0.96 | *Ift27* | -0.95 | Slc35g1 | -2.45 |
| Fbxo32 | -1.92 | *Myl2* | -0.94 | *Gadd45b* | -0.94 | Ccn5 | -2.38 |
| Rarb | -1.80 | *Sh3kbp1* | -0.93 | *Kcnma1* | -0.93 | Actr3b | -2.37 |
| Neto2 | -1.72 | *Ldhb* | -0.93 | *Lsmem1* | -0.93 | Tmem267 | -2.17 |
| Bckdha | -1.72 | *Igfbp5* | -0.92 | *Cpxm2* | -0.91 | Tnfrsf12a | -2.10 |
| Spns2 | -1.71 | *Pitpnc1* | -0.92 | *Bhlhe41* | -0.90 | Tfcp2l1 | -2.08 |
| Thtpa | -1.71 | *Crhr2* | -0.92 | *Creb3l1* | -0.86 | Irx3 | -2.02 |
| Vcl | -1.70 | *Smtnl1* | -0.90 | *Ank1* | -0.86 | Samd10 | -2.01 |
| Ankrd2 | -1.70 | *Fxyd6* | -0.88 | *Enox2* | -0.83 | Eda2r | -1.99 |
| Atg9a | -1.68 | *Fndc5* | -0.88 | *Cmbl* | -0.82 | Sbk2 | -1.98 |
| Tesk1 | -1.67 | *Ky* | -0.88 | *Micu3* | -0.80 | Ppp1r15a | -1.98 |
| H2-Q6 | -1.67 | *Itpr1* | -0.85 | *Leo1* | -0.79 | Trim63 | -1.89 |
| Slc30a2 | -1.67 | *Homer2* | -0.84 | *Wnt4* | -0.78 | Npc1 | -1.86 |
| C3 | -1.62 | *Khdrbs3* | -0.83 | *Ociad2* | -0.73 | Rgcc | -1.85 |
| Npc1 | -1.61 | *Smad3* | -0.82 | *Ttll4* | -0.73 | Pla2g7 | -1.82 |
| Ciart | -1.61 | *Pcp4l1* | -0.81 | *Trdn* | -0.72 | Slc2a3 | -1.81 |
| Tsr1 | -1.56 | *Ephx2* | -0.77 | *Nexn* | -0.70 | Amd1 | -1.80 |
| Morn4 | -1.54 | *Perm1* | -0.77 | *Car3* | -0.69 | Thtpa | -1.80 |
| Adcy9 | -1.53 | *Nceh1* | -0.76 | *Pvalb* | -0.68 | Lrfn3 | -1.78 |
| Apex1 | -1.51 | *Psd3* | -0.73 | *Txnip* | -0.68 | Amd2 | -1.78 |
| Stab2 | -1.50 | *Ptpn3* | -0.72 | *Lyar* | -0.62 | Zc2hc1c | -1.75 |
| Ppl | -1.47 | *Kdr* | -0.69 | *Cobl* | -0.61 | Ciart | -1.72 |
| Gadd45g | -1.47 | *Fabp3* | -0.69 | *Jade1* | -0.60 | Stab2 | -1.70 |
| Tmem37 | -1.47 | *Cd200* | -0.67 | *Hhatl* | -0.59 | Ankrd1 | -1.70 |
| Cers1 | -1.45 | *Srebf1* | -0.67 | *Satb1* | -0.56 | Atf3 | -1.70 |
| Bmpr1b | -1.45 | *Slc38a3* | -0.67 | *Klhl31* | -0.56 | Neto2 | -1.69 |
| Amd1 | -1.44 | *Sema6c* | -0.67 | *Ndufaf5* | -0.55 | C3 | -1.69 |
| Kif3c | -1.44 | *Idh2* | -0.67 | *Pnisr* | -0.55 | Arntl | -1.67 |
| Sacm1l | -1.43 | *Akap6* | -0.66 | *Me1* | -0.54 | Apbb3 | -1.66 |
| Zdhhc23 | -1.40 | *Smtn* | -0.65 | *Myl1* | -0.54 | Hoxd10 | -1.65 |
| Acadvl | -1.39 | *Ston2* | -0.64 | *Naa15* | -0.54 | Ctxn3 | -1.62 |
| Cd24a | -1.39 | *Ramp1* | -0.63 | *Dennd4b* | -0.54 | Ttll7 | -1.62 |
| Nos1 | -1.38 | *Clip4* | -0.63 | *Tpm1* | -0.53 | Slc30a2 | -1.60 |
| Aldoa | -1.34 | *Acaa2* | -0.62 | *Tln2* | -0.53 | Trmt61a | -1.60 |
| Tfcp2l1 | -1.33 | *Oaf* | -0.62 | *Tmem109* | -0.52 | Dhcr24 | -1.58 |
| Anxa11 | -1.33 | *Smco1* | -0.62 | *Aldoa* | -0.52 | Hook1 | -1.58 |
| Pla2g7 | -1.32 | *Mat2a* | -0.61 | *Deptor* | -0.51 | Ppl | -1.58 |
| BC048679 | -1.31 | *Sypl2* | -0.60 | *Ampd1* | -0.51 | Gzmm | -1.57 |
| Irs1 | -1.29 | *Col15a1* | -0.59 | *Srsf5* | -0.51 | Scin | -1.54 |
| Kcnf1 | -1.29 | *Angptl2* | -0.58 | *Amotl1* | -0.50 | Tekt1 | -1.50 |
| Pptc7 | -1.28 | *Tnk2* | -0.55 | *Xirp2* | -0.50 | Rorc | -1.48 |
| Vldlr | -1.26 | *Filip1l* | -0.54 | *Selenow* | -0.50 | Spns2 | -1.47 |
| Taco1 | -1.25 | *Scn4b* | -0.54 | *Ube2d1* | -0.50 | Fzd7 | -1.44 |
| Cpeb2 | -1.25 | *Eepd1* | -0.53 | *Uqcc2* | -0.48 | Nxpe3 | -1.42 |
| Mrps18b | -1.24 | *Casq2* | -0.51 | *Eif5* | -0.48 | Nqo1 | -1.39 |
| Wfikkn2 | -1.23 | *Esr1* | -0.51 | *Ghr* | -0.47 | Igfn1 | -1.39 |
| Hook1 | -1.22 | *Ank2* | -0.50 | *Ptp4a2* | -0.47 | Srxn1 | -1.39 |
| Nkain1 | -1.21 | *Ckmt2* | -0.50 | *Ndufb1-ps* | -0.47 | Rbm48 | -1.38 |
| Sec61a2 | -1.20 | *Tead1* | -0.49 | *Stac3* | -0.46 | D230025D16Rik | -1.38 |
| Lrrc61 | -1.19 | *Rasgrp3* | -0.47 | *Ppp3cb* | -0.46 | Tmem37 | -1.37 |
| Timp3 | -1.19 | *Sparcl1* | -0.46 | *Park7* | -0.45 | Mrps18b | -1.36 |
| Cacna1s | -1.19 | *Lpl* | -0.45 | *Tma7* | -0.43 | Vcl | -1.36 |
| Acss1 | -1.19 | *Tmem233* | -0.44 | *Aimp1* | -0.43 | Pdpr | -1.35 |
| Slc2a3 | -1.19 | *Camk2a* | -0.42 | *Rtn2* | -0.42 | Musk | -1.34 |
| H2-Q7 | -1.19 | *Acadm* | -0.42 | *Tmod4* | -0.41 | Bckdha | -1.33 |
| Lyz1 | -1.18 | *Oxct1* | -0.41 | *Tcea3* | -0.41 | Ifrd1 | -1.29 |
| Taf6 | -1.17 | *Chpt1* | -0.40 | *Prkar2a* | -0.40 | Rbl2 | -1.29 |
| Cry2 | -1.17 | *Tmem65* | -0.39 | *Acyp2* | -0.40 | Retreg1 | -1.28 |
| Nudt6 | -1.17 | *Stim1* | -0.36 | *Phka1* | -0.40 | Cd24a | -1.27 |
| Sncg | -1.16 | *Hadh* | -0.36 | *Phkb* | -0.40 | Ung | -1.27 |
| Ttll7 | -1.16 | *Srsf5* | -0.35 | *Tnnt3* | -0.39 | Sdr39u1 | -1.26 |
| Ces1d | -1.16 | *Rgs5* | -0.30 | *Ndufa3* | -0.39 | C7 | -1.25 |
| Arfgap2 | -1.16 | *Rps9* | 0.26 | *Tigar* | 0.40 | Htra4 | -1.25 |
| Fam20b | -1.14 | *Psmc1* | 0.35 | *Psmb4* | 0.41 | Slx4ip | -1.24 |
| Mapkapk3 | -1.13 | *Map1lc3a* | 0.36 | *Cct3* | 0.42 | Kif3c | -1.23 |
| Ubxn6 | -1.12 | *Psmc5* | 0.37 | *Cltb* | 0.43 | Cebpd | -1.23 |
| Slfn2 | -1.11 | *Psma3* | 0.37 | *Psmd2* | 0.44 | Lyz1 | -1.22 |
| Hspb7 | -1.11 | *Cct3* | 0.38 | *Psmc4* | 0.44 | Tpx2 | -1.22 |
| Slc25a34 | -1.11 | *Psmd13* | 0.39 | *Ctsd* | 0.46 | Hdac8 | -1.22 |
| Fbxw4 | -1.10 | *Serpinb6a* | 0.39 | *9530068E07Rik* | 0.47 | Stk26 | -1.22 |
| Hsd17b7 | -1.09 | *Psmb3* | 0.40 | *Hspb6* | 0.48 | Slc15a4 | -1.20 |
| Hlf | -1.09 | *Psmd6* | 0.40 | *Igfbp4* | 0.48 | Cnksr1 | -1.20 |
| Mb | -1.08 | *Psmc3* | 0.40 | *Clec3b* | 0.48 | Tmem100 | -1.19 |
| Aen | -1.08 | *Nmt1* | 0.40 | *Psmd14* | 0.50 | Vldlr | -1.18 |
| Nqo1 | -1.07 | *Psma6* | 0.41 | *Hexa* | 0.50 | Net1 | -1.18 |
| Myod1 | -1.07 | *Cd63* | 0.41 | *Arpc1b* | 0.51 | Slc12a2 | -1.18 |
| Gtf3a | -1.07 | *Herpud2* | 0.41 | *Ifitm3* | 0.51 | Nudt18 | -1.17 |
| Ppp1r15a | -1.06 | *Sod1* | 0.42 | *Psmd3* | 0.51 | 0610040J01Rik | -1.17 |
| Arel1 | -1.06 | *Psmb1* | 0.42 | *Cndp2* | 0.52 | L3mbtl2 | -1.17 |
| Irs2 | -1.06 | *Psmd3* | 0.42 | *Psmc6* | 0.52 | Aen | -1.17 |
| Dnaja4 | -1.06 | *Psmd1* | 0.43 | *Plod1* | 0.53 | Zbtb16 | -1.16 |
| 4931406P16Rik | -1.05 | *Lpin1* | 0.43 | *Rnf114* | 0.55 | Rabgef1 | -1.15 |
| Pde4b | -1.05 | *Msrb1* | 0.45 | *Tgfbr2* | 0.57 | Otud3 | -1.15 |
| Chpf2 | -1.05 | *Psmd7* | 0.45 | *Eef1a1* | 0.57 | Hsf4 | -1.15 |
| Otud3 | -1.04 | *Atf4* | 0.45 | *Cd151* | 0.57 | Aacs | -1.15 |
| Mid1ip1 | -1.04 | *Tmem38b* | 0.45 | *Myh1* | 0.58 | Cdk19 | -1.14 |
| Adrb2 | -1.04 | *Hspb8* | 0.45 | *Psmd8* | 0.58 | Heatr1 | -1.14 |
| Rnf146 | -1.04 | *Mybbp1a* | 0.45 | *Vwa5a* | 0.58 | Smox | -1.13 |
| Phlda3 | -1.03 | *Psmb4* | 0.46 | *Psma1* | 0.60 | Angptl7 | -1.13 |
| Fbxo44 | -1.03 | *Ufd1* | 0.46 | *Ly6e* | 0.60 | Mmab | -1.13 |
| Smox | -1.03 | *Ugp2* | 0.46 | *Ly6a* | 0.61 | Ubc | -1.13 |
| Eepd1 | -1.03 | *Ptges3* | 0.46 | *Col4a2* | 0.62 | Tigd4 | -1.12 |
| 0610040J01Rik | -1.02 | *Yars* | 0.47 | *Mxra8* | 0.63 | Mical2 | -1.11 |
| Als2 | -1.01 | *Rad23b* | 0.48 | *Fabp3* | 0.64 | Rnf126 | -1.11 |
| Sync | -1.01 | *Psmb5* | 0.48 | *Bcam* | 0.64 | Syne1 | -1.11 |
| Slc12a2 | -1.01 | *Rnf115* | 0.49 | *Unc93b1* | 0.68 | Mrln | -1.11 |
| St3gal5 | -1.00 | *Psmd2* | 0.49 | *Anxa7* | 0.68 | Phlda3 | -1.11 |
| Cdk19 | -1.00 | *Gnl3* | 0.49 | *Bgn* | 0.69 | Gtf3a | -1.10 |
| Asb10 | -1.00 | *Txn1* | 0.49 | *Actn2* | 0.69 | Ntmt1 | -1.10 |
| Gm38394 | -1.00 | *Mapre3* | 0.50 | *Gbe1* | 0.71 | Cfap298 | -1.10 |
| Gtf2e1 | -0.99 | *Gbe1* | 0.50 | *Psma5* | 0.71 | Cep170b | -1.08 |
| Cyyr1 | -0.99 | *Psmc4* | 0.50 | *Pi16* | 0.72 | Cdkn2c | -1.08 |
| Ttc7b | -0.99 | *Psmc6* | 0.50 | *Pde4b* | 0.73 | Dnaja4 | -1.07 |
| Kyat3 | -0.99 | *Psmd14* | 0.51 | *Htra3* | 0.74 | Ces1d | -1.07 |
| Slc40a1 | -0.99 | *Cltb* | 0.51 | *Grn* | 0.74 | Txnrd1 | -1.07 |
| Jade2 | -0.99 | *Rab7* | 0.52 | *Podn* | 0.75 | Nos1 | -1.07 |
| Cacng7 | -0.98 | *Pik3r1* | 0.53 | *Cdc42se1* | 0.76 | Pfkfb4 | -1.06 |
| Vgll4 | -0.98 | *Txnl1* | 0.53 | *Mllt11* | 0.76 | Fbxl4 | -1.06 |
| Usp9x | -0.97 | *Rabgef1* | 0.53 | *Lgmn* | 0.76 | Hmgxb3 | -1.06 |
| Tada2b | -0.96 | *Anxa7* | 0.56 | *Stat3* | 0.76 | Tsr1 | -1.06 |
| Ung | -0.96 | *Psmd12* | 0.57 | *Cbr2* | 0.78 | Gnl3 | -1.06 |
| Hmgxb3 | -0.96 | *Trafd1* | 0.57 | *Mrc1* | 0.80 | Dnph1 | -1.06 |
| Mib1 | -0.96 | *Trim54* | 0.58 | *Trim72* | 0.81 | Pwp2 | -1.05 |
| Mgst1 | -0.96 | *Lrrc58* | 0.58 | *Klf2* | 0.81 | Ccnk | -1.05 |
| Gpx3 | -0.96 | *Nsfl1c* | 0.58 | *Ptpn3* | 0.82 | Cry2 | -1.05 |
| Tuba8 | -0.96 | *Nop9* | 0.59 | *Cfh* | 0.83 | Slc43a1 | -1.05 |
| Aebp1 | -0.95 | *Gpt2* | 0.59 | *Htra1* | 0.83 | Asb5 | -1.05 |
| Pde4d | -0.95 | *Dpp3* | 0.59 | *Nploc4* | 0.84 | Mmp9 | -1.05 |
| Rab5if | -0.95 | *Pfkfb4* | 0.59 | *Acadvl* | 0.85 | Trim54 | -1.04 |
| Tdrp | -0.95 | *Pdlim3* | 0.59 | *Plvap* | 0.85 | Cox11 | -1.03 |
| Bnip3 | -0.95 | *Tubb4b* | 0.59 | *Aspscr1* | 0.86 | Asb11 | -1.03 |
| Tatdn2 | -0.94 | *Txnrd1* | 0.60 | *Laptm5* | 0.86 | Dusp10 | -1.03 |
| Plekhb2 | -0.94 | *Sesn1* | 0.60 | *Gpx3* | 0.86 | Sdad1 | -1.02 |
| Hs6st1 | -0.94 | *Psma7* | 0.60 | *Fcer1g* | 0.87 | Sacm1l | -1.02 |
| Klhl21 | -0.94 | *Slc38a2* | 0.61 | *Tango2* | 0.87 | Lrrc2 | -1.02 |
| Mfsd4a | -0.94 | *Gsr* | 0.61 | *Csf1r* | 0.89 | Atg9a | -1.02 |
| Atg4d | -0.94 | *Sorbs3* | 0.62 | *Sdc3* | 0.89 | Hr | -1.02 |
| Scn4a | -0.92 | *Flnc* | 0.62 | *Vwf* | 0.90 | Noc2l | -1.02 |
| Ptp4a3 | -0.92 | *Timm10* | 0.63 | *Lyve1* | 0.91 | Orc2 | -1.01 |
| Bdh1 | -0.92 | *Retreg1* | 0.63 | *Cyth4* | 0.92 | Timm10 | -1.01 |
| Myh14 | -0.92 | *Psmc2* | 0.63 | *Pf4* | 0.94 | Tuba8 | -1.01 |
| Slc4a4 | -0.92 | *Psmd11* | 0.64 | *Prkg1* | 0.95 | Xirp1 | -1.00 |
| Egln3 | -0.92 | *Ubb* | 0.64 | *Cotl1* | 0.97 | Arel1 | -1.00 |
| Lmod1 | -0.92 | *Nploc4* | 0.64 | *Stab1* | 1.00 | Slc8a1 | -1.00 |
| Snx11 | -0.91 | *Hectd1* | 0.64 | *Lbp* | 1.03 | Plcd3 | -1.00 |
| Piezo1 | -0.91 | *Acyp1* | 0.64 | *Dok4* | 1.04 | Tfdp2 | -1.00 |
| Slc8a1 | -0.91 | *Utp11* | 0.65 | *Tspan4* | 1.04 | Taf6 | -0.99 |
| Abcb4 | -0.91 | *Psmd4* | 0.65 | *Lyz2* | 1.05 | Nt5dc3 | -0.99 |
| Mylk4 | -0.90 | *Adrm1* | 0.65 | *Ctss* | 1.06 | Eef1aknmt | -0.99 |
| Kifc3 | -0.90 | *Fam214b* | 0.65 | *Npr3* | 1.06 | Gclc | -0.99 |
| Mmab | -0.90 | *Tfdp2* | 0.66 | *C1qc* | 1.07 | Plekhb1 | -0.99 |
| Acacb | -0.90 | *Ctsl* | 0.67 | *Plin5* | 1.08 | Arfgap2 | -0.98 |
| Mllt11 | -0.90 | *Myf6* | 0.67 | *Nrros* | 1.09 | Filip1 | -0.98 |
| Cnksr1 | -0.90 | *Xirp1* | 0.67 | *C1qb* | 1.10 | Zwint | -0.98 |
| Xk | -0.89 | *Nup210* | 0.67 | *Ier3* | 1.10 | En1 | -0.98 |
| Sdr39u1 | -0.89 | *Cfh* | 0.69 | *Hlf* | 1.10 | Tma16 | -0.98 |
| Bhlhe40 | -0.89 | *Fgl2* | 0.69 | *Lcp1* | 1.11 | Optn | -0.97 |
| Zdhhc5 | -0.88 | *Asb11* | 0.70 | *Id3* | 1.12 | Wdr46 | -0.97 |
| Yif1b | -0.88 | *Psma1* | 0.71 | *Avil* | 1.13 | Foxk1 | -0.97 |
| Ddx54 | -0.88 | *Hgs* | 0.71 | *Hcls1* | 1.13 | Tuba4a | -0.97 |
| Ier3 | -0.88 | *Nabp1* | 0.72 | *Acss1* | 1.14 | Snap29 | -0.97 |
| Rorc | -0.87 | *6430548M08Rik* | 0.72 | *Cd68* | 1.15 | Adcy9 | -0.96 |
| Pcyt1a | -0.87 | *Plvap* | 0.72 | *Pnpla2* | 1.15 | Dhx32 | -0.96 |
| Nrap | -0.87 | *Aspscr1* | 0.72 | *Bcl6b* | 1.22 | Smtnl2 | -0.96 |
| Rnf150 | -0.87 | *Ppp1r15a* | 0.72 | *F13a1* | 1.23 | Zfand5 | -0.96 |
| Plin5 | -0.86 | *Sqstm1* | 0.73 | *C1qa* | 1.23 | Slf1 | -0.95 |
| Golm1 | -0.86 | *Osgin1* | 0.73 | *Fcgr3* | 1.23 | Psmd8 | -0.95 |
| Pcnx | -0.85 | *Psmd8* | 0.74 | *Tmem106a* | 1.24 | Leo1 | -0.95 |
| Hbp1 | -0.85 | *Psma5* | 0.74 | *Fabp5* | 1.24 | Znhit3 | -0.95 |
| Srebf1 | -0.85 | *Fibin* | 0.75 | *Hspb7* | 1.26 | Six2 | -0.95 |
| Ppp1r3c | -0.85 | *Tango2* | 0.75 | *Egln3* | 1.37 | Ccdc85c | -0.94 |
| Nomo1 | -0.85 | *Ip6k3* | 0.76 | *Slfn2* | 1.43 | Cacna1s | -0.94 |
| Susd6 | -0.85 | *F13a1* | 0.76 | *Adgre1* | 1.46 | Ubxn6 | -0.94 |
| Esrrg | -0.84 | *Zcchc24* | 0.77 | *Ccl6* | 1.52 | Cdkn2aipnl | -0.94 |
| Spen | -0.84 | *Cdkal1* | 0.80 | *Id1* | 1.74 | Nr1d1 | -0.94 |
| Kif1c | -0.84 | *Tnfrsf12a* | 0.81 | *Slc25a25* | 2.07 | Anxa11 | -0.94 |
| Herc3 | -0.84 | *Ubxn4* | 0.81 | *Serpina3n* | 2.82 | Mtr | -0.94 |
| Fam219a | -0.83 | *Gclm* | 0.87 | *Lcn2* | 3.73 | Rnf144b | -0.94 |
| Pstpip2 | -0.83 | *Tmem37* | 0.87 | *Pnmt* | 3.81 | Zswim4 | -0.93 |
| Paip2b | -0.83 | *Slc7a2* | 0.87 |  |  | Hipk2 | -0.93 |
| Mr1 | -0.83 | *Ccl6* | 0.91 |  |  | Lpin1 | -0.93 |
| Retnla | -0.83 | *Hspb1* | 0.98 |  |  | Wfikkn2 | -0.93 |
| Ap2a1 | -0.82 | *Srxn1* | 0.99 |  |  | Atf4 | -0.93 |
| Filip1 | -0.82 | *Slc15a4* | 0.99 |  |  | Cry1 | -0.93 |
| Atp1b1 | -0.82 | *Egln3* | 1.00 |  |  | Stat5b | -0.93 |
| Syne1 | -0.82 | *Tmem100* | 1.01 |  |  | Hspb1 | -0.93 |
| Icmt | -0.82 | *Zbtb16* | 1.01 |  |  | Prkca | -0.93 |
| Cpeb1 | -0.82 | *Apod* | 1.08 |  |  | Slc40a1 | -0.93 |
| Pwp2 | -0.82 | *Zwint* | 1.10 |  |  | Smcr8 | -0.92 |
| Tm9sf1 | -0.81 | *1700001O22Rik* | 1.11 |  |  | Rilpl1 | -0.92 |
| Kbtbd13 | -0.81 | *Htra1* | 1.13 |  |  | Abcb4 | -0.92 |
| Ankrd52 | -0.81 | *Mt1* | 1.14 |  |  | Gpatch1 | -0.92 |
| Acad9 | -0.81 | *Trim63* | 1.16 |  |  | Klhl21 | -0.92 |
| Nceh1 | -0.81 | *Lox* | 1.17 |  |  | Cpeb1 | -0.92 |
| Timm23 | -0.80 | *Rgcc* | 1.23 |  |  | Flnc | -0.91 |
| Dpp9 | -0.80 | *Eif4ebp1* | 1.32 |  |  | E2f4 | -0.91 |
| Raf1 | -0.80 | *Cebpd* | 1.33 |  |  | Lin52 | -0.91 |
| Rprd1a | -0.80 | *Ctxn3* | 1.37 |  |  | Tmcc2 | -0.91 |
| Acot2 | -0.79 | *Apbb3* | 1.47 |  |  | Wnt4 | -0.91 |
| Them6 | -0.79 | *Serpina3n* | 2.48 |  |  | Hsph1 | -0.91 |
| Wsb2 | -0.79 | *Depp1* | 3.07 |  |  | Tmco4 | -0.90 |
| Psmd8 | -0.79 |  |  |  |  | Pim3 | -0.90 |
| Prkg1 | -0.78 |  |  |  |  | Slc19a2 | -0.90 |
| Abhd5 | -0.78 |  |  |  |  | Ugp2 | -0.90 |
| Lrrc2 | -0.78 |  |  |  |  | Cdkal1 | -0.90 |
| Hic1 | -0.78 |  |  |  |  | Aars | -0.90 |
| Noc2l | -0.78 |  |  |  |  | Fam122a | -0.90 |
| Fzd7 | -0.78 |  |  |  |  | Enox2 | -0.90 |
| Heatr1 | -0.78 |  |  |  |  | Oser1 | -0.90 |
| Trim54 | -0.78 |  |  |  |  | Fut10 | -0.90 |
| Srxn1 | -0.78 |  |  |  |  | Zrsr1 | -0.90 |
| Zfp651 | -0.77 |  |  |  |  | Tubb4b | -0.89 |
| Slc35e1 | -0.77 |  |  |  |  | Patz1 | -0.89 |
| Tbc1d16 | -0.77 |  |  |  |  | Ulk1 | -0.89 |
| Ugp2 | -0.77 |  |  |  |  | Tesk1 | -0.89 |
| Ece1 | -0.77 |  |  |  |  | Kank1 | -0.89 |
| Smyd2 | -0.77 |  |  |  |  | Acsl3 | -0.89 |
| Ccdc85c | -0.77 |  |  |  |  | Supt3 | -0.89 |
| Itpr1 | -0.77 |  |  |  |  | Pcx | -0.88 |
| Cog1 | -0.76 |  |  |  |  | Ybx3 | -0.88 |
| Rnf144b | -0.76 |  |  |  |  | Ddx56 | -0.88 |
| Hsph1 | -0.76 |  |  |  |  | Utp20 | -0.88 |
| Dnajc27 | -0.76 |  |  |  |  | Mr1 | -0.88 |
| Rxra | -0.76 |  |  |  |  | Cpeb2 | -0.88 |
| Fam53b | -0.76 |  |  |  |  | Yars | -0.87 |
| Bcl2l13 | -0.76 |  |  |  |  | Phc1 | -0.87 |
| Perm1 | -0.76 |  |  |  |  | Epm2a | -0.87 |
| Podxl2 | -0.75 |  |  |  |  | Zfyve1 | -0.87 |
| Atpaf1 | -0.75 |  |  |  |  | Cacng7 | -0.87 |
| Eif4ebp2 | -0.75 |  |  |  |  | Ctdp1 | -0.87 |
| Hmga1 | -0.75 |  |  |  |  | Hsd17b7 | -0.86 |
| Hr | -0.74 |  |  |  |  | Gpt2 | -0.86 |
| Scn4b | -0.74 |  |  |  |  | Dym | -0.86 |
| Rnf126 | -0.74 |  |  |  |  | Lrrc47 | -0.86 |
| 2310002L09Rik | -0.74 |  |  |  |  | Ddx20 | -0.86 |
| Nr1d1 | -0.74 |  |  |  |  | H1f2 | -0.86 |
| Plvap | -0.74 |  |  |  |  | Amy1 | -0.86 |
| Zfp346 | -0.74 |  |  |  |  | Alpk2 | -0.86 |
| Flnc | -0.74 |  |  |  |  | Akt2 | -0.85 |
| Hip1r | -0.73 |  |  |  |  | Sra1 | -0.85 |
| Amy1 | -0.73 |  |  |  |  | Stk40 | -0.85 |
| E2f6 | -0.73 |  |  |  |  | Sf3a2 | -0.85 |
| Pkdcc | -0.73 |  |  |  |  | Unc5a | -0.85 |
| Nf2 | -0.73 |  |  |  |  | Maob | -0.85 |
| Lynx1 | -0.73 |  |  |  |  | Diaph1 | -0.85 |
| Ptpn3 | -0.73 |  |  |  |  | Utp4 | -0.85 |
| Ank | -0.73 |  |  |  |  | Hspb8 | -0.85 |
| Pex11a | -0.73 |  |  |  |  | Pptc7 | -0.85 |
| Fdft1 | -0.73 |  |  |  |  | Tkt | -0.84 |
| Fbxl4 | -0.73 |  |  |  |  | Polr3h | -0.84 |
| Cbll1 | -0.72 |  |  |  |  | Tob2 | -0.84 |
| Uaca | -0.72 |  |  |  |  | Lrrc40 | -0.84 |
| Tmcc2 | -0.72 |  |  |  |  | Shmt1 | -0.84 |
| Mllt1 | -0.72 |  |  |  |  | Ccdc181 | -0.84 |
| Usp12 | -0.71 |  |  |  |  | Pde7a | -0.84 |
| Rab12 | -0.71 |  |  |  |  | Rpp38 | -0.83 |
| Maob | -0.71 |  |  |  |  | Wdr75 | -0.83 |
| Tmem120a | -0.71 |  |  |  |  | Cst6 | -0.83 |
| Ifrd2 | -0.71 |  |  |  |  | Nomo1 | -0.83 |
| Sptb | -0.71 |  |  |  |  | Fam160b2 | -0.83 |
| Mtrex | -0.71 |  |  |  |  | Timm23 | -0.83 |
| Hspa9 | -0.71 |  |  |  |  | Ar | -0.83 |
| Srf | -0.70 |  |  |  |  | Gemin6 | -0.83 |
| Fhl3 | -0.70 |  |  |  |  | Mtrr | -0.83 |
| Retreg1 | -0.70 |  |  |  |  | Lrrc58 | -0.83 |
| Hs3st5 | -0.70 |  |  |  |  | Rfx1 | -0.83 |
| Tigar | -0.70 |  |  |  |  | Zfp651 | -0.83 |
| Ssu72 | -0.69 |  |  |  |  | Hbp1 | -0.83 |
| Pde7a | -0.69 |  |  |  |  | Tmem192 | -0.83 |
| H2-Eb1 | -0.69 |  |  |  |  | Fam20b | -0.83 |
| Diaph1 | -0.69 |  |  |  |  | Camsap1 | -0.83 |
| Pcx | -0.69 |  |  |  |  | Pygo1 | -0.82 |
| Pnpla8 | -0.68 |  |  |  |  | Twf2 | -0.82 |
| Gpc1 | -0.68 |  |  |  |  | Cyp27a1 | -0.82 |
| Zfp106 | -0.68 |  |  |  |  | Ccdc71l | -0.82 |
| Sgsm3 | -0.68 |  |  |  |  | Gpatch4 | -0.82 |
| Anxa7 | -0.68 |  |  |  |  | Bnip3 | -0.82 |
| Cdnf | -0.68 |  |  |  |  | Adrm1 | -0.82 |
| Pitrm1 | -0.67 |  |  |  |  | Pstpip2 | -0.82 |
| Cbx7 | -0.67 |  |  |  |  | Ltv1 | -0.82 |
| Vwa1 | -0.67 |  |  |  |  | Dhdh | -0.82 |
| Nploc4 | -0.67 |  |  |  |  | 2310002L09Rik | -0.82 |
| Rps6ka2 | -0.67 |  |  |  |  | Ubb | -0.81 |
| Lrig1 | -0.67 |  |  |  |  | Bhlhe40 | -0.81 |
| Supt5 | -0.67 |  |  |  |  | Tiparp | -0.81 |
| Sema3g | -0.67 |  |  |  |  | Cxcl14 | -0.81 |
| Dhx32 | -0.67 |  |  |  |  | Atrip | -0.80 |
| Rilpl1 | -0.66 |  |  |  |  | Mapkapk3 | -0.80 |
| Tmod1 | -0.66 |  |  |  |  | Ddx3x | -0.80 |
| Rhou | -0.66 |  |  |  |  | Myf6 | -0.80 |
| Dtnbp1 | -0.66 |  |  |  |  | Atg13 | -0.80 |
| Hif1an | -0.66 |  |  |  |  | Ints5 | -0.79 |
| Ptpn1 | -0.66 |  |  |  |  | Pde4b | -0.79 |
| Aldh1a2 | -0.66 |  |  |  |  | Raf1 | -0.79 |
| Ccnk | -0.66 |  |  |  |  | Eya4 | -0.79 |
| Rnf114 | -0.66 |  |  |  |  | 2310057M21Rik | -0.79 |
| Got1 | -0.66 |  |  |  |  | Lsg1 | -0.79 |
| 5031439G07Rik | -0.66 |  |  |  |  | Snrpn | -0.79 |
| Synpo | -0.66 |  |  |  |  | Trmt10c | -0.79 |
| Ifrd1 | -0.66 |  |  |  |  | Gde1 | -0.79 |
| Abcf2 | -0.66 |  |  |  |  | Tmub1 | -0.78 |
| Inpp5a | -0.66 |  |  |  |  | Fhl3 | -0.78 |
| Nt5dc3 | -0.66 |  |  |  |  | Eepd1 | -0.78 |
| Ppip5k1 | -0.66 |  |  |  |  | Asb10 | -0.78 |
| Usp24 | -0.66 |  |  |  |  | Abhd16a | -0.78 |
| Myh2 | -0.65 |  |  |  |  | Tmod1 | -0.78 |
| Asb15 | -0.65 |  |  |  |  | Rpp30 | -0.78 |
| Blcap | -0.64 |  |  |  |  | Uaca | -0.78 |
| Map3k10 | -0.64 |  |  |  |  | Rnf113a2 | -0.78 |
| Plaat3 | -0.64 |  |  |  |  | Mtrex | -0.78 |
| Cyb5d2 | -0.64 |  |  |  |  | Nop14 | -0.78 |
| Cops3 | -0.64 |  |  |  |  | Dnajb9 | -0.78 |
| Myh11 | -0.64 |  |  |  |  | Ppp1r3c | -0.78 |
| Aco2 | -0.64 |  |  |  |  | Sephs2 | -0.78 |
| Lrrc30 | -0.64 |  |  |  |  | Dcun1d4 | -0.78 |
| Dhdh | -0.63 |  |  |  |  | Rab12 | -0.78 |
| Stk40 | -0.63 |  |  |  |  | Lyar | -0.77 |
| Epm2a | -0.63 |  |  |  |  | Ubac2 | -0.77 |
| Twf2 | -0.63 |  |  |  |  | Mtmr1 | -0.77 |
| Tbc1d4 | -0.63 |  |  |  |  | Hs3st5 | -0.77 |
| Pdzrn3 | -0.63 |  |  |  |  | Nampt | -0.77 |
| Ptges2 | -0.63 |  |  |  |  | Mia2 | -0.77 |
| Tysnd1 | -0.63 |  |  |  |  | Srebf1 | -0.76 |
| Rnf139 | -0.63 |  |  |  |  | Atg4d | -0.76 |
| Rnf123 | -0.63 |  |  |  |  | Mybbp1a | -0.76 |
| Tnfrsf12a | -0.63 |  |  |  |  | Adi1 | -0.76 |
| Zfp397 | -0.62 |  |  |  |  | Tmem186 | -0.76 |
| Aldh1a1 | -0.62 |  |  |  |  | Ece1 | -0.76 |
| E2f4 | -0.62 |  |  |  |  | Exosc1 | -0.76 |
| Asb11 | -0.62 |  |  |  |  | Sbds | -0.76 |
| Camk2b | -0.62 |  |  |  |  | Vps72 | -0.75 |
| Urgcp | -0.62 |  |  |  |  | Ftsj3 | -0.75 |
| Ubxn2a | -0.62 |  |  |  |  | Kif1c | -0.75 |
| Twnk | -0.62 |  |  |  |  | Dnaaf5 | -0.75 |
| Lpin1 | -0.62 |  |  |  |  | Dyrk1a | -0.75 |
| Kcnj12 | -0.62 |  |  |  |  | Ficd | -0.75 |
| Mkrn2 | -0.62 |  |  |  |  | Pde4d | -0.75 |
| Stau2 | -0.62 |  |  |  |  | Xirp2 | -0.75 |
| Dhx29 | -0.62 |  |  |  |  | Ttc7b | -0.75 |
| Slc27a1 | -0.61 |  |  |  |  | Gabarapl1 | -0.75 |
| Wls | -0.61 |  |  |  |  | Srf | -0.75 |
| Optn | -0.61 |  |  |  |  | Rxrg | -0.75 |
| Txnrd1 | -0.61 |  |  |  |  | Uck2 | -0.75 |
| Cpeb4 | -0.61 |  |  |  |  | Polr3d | -0.75 |
| Cbr2 | -0.61 |  |  |  |  | Tinf2 | -0.75 |
| Synm | -0.61 |  |  |  |  | Aff1 | -0.75 |
| Tlk1 | -0.61 |  |  |  |  | Kmt5a | -0.75 |
| Nop2 | -0.61 |  |  |  |  | Slc38a2 | -0.74 |
| Gde1 | -0.61 |  |  |  |  | C1qtnf4 | -0.74 |
| Sil1 | -0.61 |  |  |  |  | 4931406P16Rik | -0.74 |
| Uck2 | -0.61 |  |  |  |  | Lmtk2 | -0.74 |
| Atg13 | -0.61 |  |  |  |  | Klhl30 | -0.74 |
| Cd74 | -0.60 |  |  |  |  | Slc39a14 | -0.74 |
| Gys1 | -0.60 |  |  |  |  | Acacb | -0.74 |
| Ivns1abp | -0.60 |  |  |  |  | Samd4 | -0.73 |
| Lmtk2 | -0.60 |  |  |  |  | Klf9 | -0.73 |
| Prxl2a | -0.60 |  |  |  |  | Cog6 | -0.73 |
| Zfp768 | -0.60 |  |  |  |  | Irs2 | -0.73 |
| Narf | -0.60 |  |  |  |  | Mrps34 | -0.73 |
| Golga4 | -0.60 |  |  |  |  | Gsk3b | -0.72 |
| Plpp1 | -0.60 |  |  |  |  | Dnttip2 | -0.72 |
| Ccl11 | -0.60 |  |  |  |  | Abcf2 | -0.72 |
| Rabgef1 | -0.60 |  |  |  |  | Syngr1 | -0.72 |
| Nr4a1 | -0.60 |  |  |  |  | Rab2a | -0.72 |
| Emd | -0.60 |  |  |  |  | Atp2a1 | -0.72 |
| Dnajc28 | -0.60 |  |  |  |  | Lrrc30 | -0.72 |
| 1810013L24Rik | -0.60 |  |  |  |  | Prdm2 | -0.72 |
| Fam160b2 | -0.59 |  |  |  |  | Ppme1 | -0.72 |
| Gnl3l | -0.59 |  |  |  |  | Anapc5 | -0.72 |
| Sf3a2 | -0.59 |  |  |  |  | Fkrp | -0.72 |
| Faf1 | -0.59 |  |  |  |  | Fem1a | -0.72 |
| Bysl | -0.59 |  |  |  |  | Prxl2a | -0.72 |
| Gtpbp1 | -0.59 |  |  |  |  | Dnajc27 | -0.72 |
| Gpcpd1 | -0.59 |  |  |  |  | Eif2ak1 | -0.72 |
| Crim1 | -0.59 |  |  |  |  | Wnk2 | -0.71 |
| Aars | -0.59 |  |  |  |  | Atic | -0.71 |
| Anapc5 | -0.59 |  |  |  |  | Thap12 | -0.71 |
| Phc1 | -0.59 |  |  |  |  | Nle1 | -0.71 |
| Mapk14 | -0.59 |  |  |  |  | E2f6 | -0.71 |
| Tpd52l1 | -0.59 |  |  |  |  | Sqstm1 | -0.71 |
| Tubb4b | -0.59 |  |  |  |  | Asb4 | -0.71 |
| Zyg11b | -0.58 |  |  |  |  | Mafg | -0.71 |
| Kank1 | -0.58 |  |  |  |  | Slc35e4 | -0.71 |
| Gart | -0.58 |  |  |  |  | Dnajb12 | -0.71 |
| Supt6 | -0.58 |  |  |  |  | Synm | -0.71 |
| Mef2d | -0.58 |  |  |  |  | Supt5 | -0.71 |
| Ttc33 | -0.58 |  |  |  |  | Pip5k1a | -0.71 |
| Fxr2 | -0.58 |  |  |  |  | Nol6 | -0.70 |
| Rcan2 | -0.58 |  |  |  |  | Iars | -0.70 |
| Iars | -0.58 |  |  |  |  | Nolc1 | -0.70 |
| Slc35f5 | -0.57 |  |  |  |  | Rpf1 | -0.70 |
| Trappc10 | -0.57 |  |  |  |  | Sf1 | -0.70 |
| Itgb5 | -0.57 |  |  |  |  | Grwd1 | -0.70 |
| Etfdh | -0.57 |  |  |  |  | Pnpla8 | -0.70 |
| Syngr1 | -0.57 |  |  |  |  | Ddx24 | -0.69 |
| Morf4l1 | -0.57 |  |  |  |  | Nup153 | -0.69 |
| Wnk2 | -0.57 |  |  |  |  | 6030458C11Rik | -0.69 |
| Golga5 | -0.57 |  |  |  |  | Fdft1 | -0.69 |
| Parvb | -0.57 |  |  |  |  | Zdhhc5 | -0.69 |
| Coq8a | -0.57 |  |  |  |  | Mroh1 | -0.69 |
| Acsl1 | -0.57 |  |  |  |  | Mgst1 | -0.69 |
| Tuba4a | -0.57 |  |  |  |  | Scn4a | -0.69 |
| Stom | -0.57 |  |  |  |  | Ythdf1 | -0.69 |
| Rad23a | -0.57 |  |  |  |  | Thrb | -0.69 |
| Ykt6 | -0.57 |  |  |  |  | Mospd1 | -0.69 |
| Flad1 | -0.56 |  |  |  |  | Asb15 | -0.69 |
| Nfe2l1 | -0.56 |  |  |  |  | Me1 | -0.69 |
| Clec16a | -0.56 |  |  |  |  | Osgin1 | -0.69 |
| Plekhb1 | -0.56 |  |  |  |  | Pdcd7 | -0.69 |
| Arpc4 | -0.56 |  |  |  |  | Nol11 | -0.69 |
| Cab39 | -0.56 |  |  |  |  | Umad1 | -0.69 |
| Pdha1 | -0.56 |  |  |  |  | Fbxo40 | -0.69 |
| Wdtc1 | -0.56 |  |  |  |  | Rad23a | -0.69 |
| Npepl1 | -0.56 |  |  |  |  | Zfp777 | -0.69 |
| Rrm2b | -0.56 |  |  |  |  | Podxl2 | -0.69 |
| Aff1 | -0.56 |  |  |  |  | Dcaf5 | -0.69 |
| Myh1 | -0.55 |  |  |  |  | Eif4ebp2 | -0.69 |
| Slc8a3 | -0.55 |  |  |  |  | 4930453N24Rik | -0.69 |
| Tomm20 | -0.55 |  |  |  |  | Abcf3 | -0.68 |
| Lrpprc | -0.55 |  |  |  |  | Rmnd5b | -0.68 |
| Cep104 | -0.55 |  |  |  |  | Brd1 | -0.68 |
| Arhgef17 | -0.55 |  |  |  |  | Lemd3 | -0.68 |
| Gnl3 | -0.55 |  |  |  |  | Kcmf1 | -0.68 |
| Txlnb | -0.55 |  |  |  |  | Rab5a | -0.68 |
| Acat1 | -0.55 |  |  |  |  | Aebp1 | -0.68 |
| Akt2 | -0.55 |  |  |  |  | Kcnma1 | -0.68 |
| Zbtb7b | -0.55 |  |  |  |  | Tsc1 | -0.68 |
| Acox1 | -0.55 |  |  |  |  | Ddx21 | -0.68 |
| Nrd1 | -0.55 |  |  |  |  | Rrp12 | -0.68 |
| Snrnp200 | -0.55 |  |  |  |  | Ubqln4 | -0.68 |
| Tmem170b | -0.55 |  |  |  |  | Pnpla7 | -0.68 |
| Mgat4b | -0.54 |  |  |  |  | Ifrd2 | -0.68 |
| Popdc3 | -0.54 |  |  |  |  | Mknk2 | -0.68 |
| Pcm1 | -0.54 |  |  |  |  | Rab5if | -0.67 |
| Thap12 | -0.54 |  |  |  |  | Ubald1 | -0.67 |
| Bcor | -0.54 |  |  |  |  | Plekhm1 | -0.67 |
| Heatr3 | -0.54 |  |  |  |  | Etl4 | -0.67 |
| Asb5 | -0.54 |  |  |  |  | Ppp1r3a | -0.67 |
| Pnpla7 | -0.54 |  |  |  |  | Prmt7 | -0.67 |
| Tmem65 | -0.54 |  |  |  |  | Sptb | -0.67 |
| Traf2 | -0.54 |  |  |  |  | Cdc23 | -0.67 |
| Crbn | -0.54 |  |  |  |  | AW209491 | -0.67 |
| Mknk2 | -0.54 |  |  |  |  | Ddx59 | -0.67 |
| Sort1 | -0.54 |  |  |  |  | Gart | -0.67 |
| Rrp12 | -0.54 |  |  |  |  | Rdm1 | -0.67 |
| Pfkfb1 | -0.53 |  |  |  |  | Ermp1 | -0.67 |
| 2610507B11Rik | -0.53 |  |  |  |  | Ube2g1 | -0.67 |
| Usp47 | -0.53 |  |  |  |  | Ank | -0.66 |
| Cdc73 | -0.53 |  |  |  |  | Nup210 | -0.66 |
| Fem1a | -0.53 |  |  |  |  | Gid4 | -0.66 |
| Svip | -0.53 |  |  |  |  | Urgcp | -0.66 |
| Rab35 | -0.53 |  |  |  |  | Usp24 | -0.66 |
| Iqsec1 | -0.53 |  |  |  |  | Gpcpd1 | -0.66 |
| Mospd1 | -0.53 |  |  |  |  | Pnrc1 | -0.66 |
| Ptpn21 | -0.53 |  |  |  |  | Ociad2 | -0.66 |
| Tmem201 | -0.53 |  |  |  |  | Mars1 | -0.66 |
| Gnl1 | -0.53 |  |  |  |  | Sbno2 | -0.66 |
| Ppp1r3a | -0.52 |  |  |  |  | Gnl2 | -0.66 |
| Adi1 | -0.52 |  |  |  |  | Fsd2 | -0.66 |
| Samd8 | -0.52 |  |  |  |  | Prob1 | -0.65 |
| Mphosph10 | -0.52 |  |  |  |  | Pcyt1a | -0.65 |
| Kctd9 | -0.52 |  |  |  |  | Wsb2 | -0.65 |
| Wdr44 | -0.52 |  |  |  |  | Nop56 | -0.65 |
| Zmiz2 | -0.52 |  |  |  |  | Wdr12 | -0.65 |
| Spcs3 | -0.52 |  |  |  |  | Atf1 | -0.65 |
| Dnajb2 | -0.52 |  |  |  |  | Kat8 | -0.65 |
| Cdc42se2 | -0.52 |  |  |  |  | Cdnf | -0.65 |
| Fsd2 | -0.52 |  |  |  |  | Rxra | -0.65 |
| Herc1 | -0.52 |  |  |  |  | Pttg1 | -0.65 |
| Nek9 | -0.52 |  |  |  |  | Bcl2l13 | -0.65 |
| Slc6a6 | -0.52 |  |  |  |  | Patl1 | -0.64 |
| Srsf2 | -0.51 |  |  |  |  | Zfp180 | -0.64 |
| Zbtb17 | -0.51 |  |  |  |  | Tnpo3 | -0.64 |
| Snw1 | -0.51 |  |  |  |  | Trap1 | -0.64 |
| Ap1ar | -0.51 |  |  |  |  | Gadd45b | -0.64 |
| Mvb12b | -0.51 |  |  |  |  | Card19 | -0.64 |
| Kctd15 | -0.51 |  |  |  |  | Mib1 | -0.64 |
| Arih2 | -0.51 |  |  |  |  | Ap2a1 | -0.64 |
| Psmb7 | -0.51 |  |  |  |  | Herc3 | -0.64 |
| Sympk | -0.51 |  |  |  |  | Slc11a2 | -0.64 |
| Ilrun | -0.51 |  |  |  |  | 5031439G07Rik | -0.64 |
| Tbrg1 | -0.51 |  |  |  |  | Thumpd1 | -0.63 |
| Nadk2 | -0.51 |  |  |  |  | Vps18 | -0.63 |
| Tom1l2 | -0.51 |  |  |  |  | Clint1 | -0.63 |
| Trap1 | -0.51 |  |  |  |  | Serpinb6a | -0.63 |
| Galnt1 | -0.50 |  |  |  |  | Tars | -0.63 |
| Tspan8 | -0.50 |  |  |  |  | Tspan8 | -0.63 |
| Dym | -0.50 |  |  |  |  | Prpf6 | -0.63 |
| Serpinb6a | -0.50 |  |  |  |  | Cab39 | -0.63 |
| Vegfb | -0.50 |  |  |  |  | Pwp1 | -0.63 |
| Kcnj2 | -0.50 |  |  |  |  | Rpa2 | -0.63 |
| Abhd16a | -0.50 |  |  |  |  | Rbm24 | -0.63 |
| Stk11 | -0.50 |  |  |  |  | Msrb3 | -0.63 |
| Scrn3 | -0.49 |  |  |  |  | Pik3c3 | -0.62 |
| Gsk3a | -0.49 |  |  |  |  | Lsm12 | -0.62 |
| Lonp2 | -0.49 |  |  |  |  | Ubxn2a | -0.62 |
| Gaa | -0.49 |  |  |  |  | Bysl | -0.62 |
| Ptpn11 | -0.49 |  |  |  |  | Cdc73 | -0.62 |
| Cipc | -0.49 |  |  |  |  | Hoxc11 | -0.62 |
| Agpat3 | -0.49 |  |  |  |  | Rab7 | -0.62 |
| Ubac2 | -0.49 |  |  |  |  | Gpn3 | -0.62 |
| Yars | -0.49 |  |  |  |  | Mllt11 | -0.62 |
| Csnk1d | -0.49 |  |  |  |  | Scyl1 | -0.62 |
| Creld1 | -0.49 |  |  |  |  | Nup62 | -0.62 |
| L2hgdh | -0.49 |  |  |  |  | Eif5 | -0.62 |
| Ddx3x | -0.49 |  |  |  |  | Vps33a | -0.62 |
| Fitm2 | -0.49 |  |  |  |  | Pi4k2a | -0.62 |
| Pdxdc1 | -0.49 |  |  |  |  | Mettl1 | -0.62 |
| Rac1 | -0.49 |  |  |  |  | Faf1 | -0.62 |
| Fbxw5 | -0.49 |  |  |  |  | Nek9 | -0.62 |
| Nfkbib | -0.49 |  |  |  |  | Susd6 | -0.62 |
| Pcgf5 | -0.48 |  |  |  |  | Paip2b | -0.62 |
| Slc2a4 | -0.48 |  |  |  |  | 1810013L24Rik | -0.62 |
| Tmem248 | -0.48 |  |  |  |  | Slc25a26 | -0.62 |
| Card19 | -0.48 |  |  |  |  | Phkb | -0.62 |
| Fnip1 | -0.48 |  |  |  |  | Mphosph10 | -0.61 |
| Etf1 | -0.48 |  |  |  |  | Ppp1r37 | -0.61 |
| Sord | -0.48 |  |  |  |  | Crbn | -0.61 |
| Dctn1 | -0.47 |  |  |  |  | Slc25a16 | -0.61 |
| Cog6 | -0.47 |  |  |  |  | Gpc1 | -0.61 |
| Uqcrc2 | -0.47 |  |  |  |  | Dhps | -0.61 |
| Rapgef1 | -0.47 |  |  |  |  | Mgat4b | -0.61 |
| Phospho2 | -0.47 |  |  |  |  | Xbp1 | -0.61 |
| Slc25a20 | -0.47 |  |  |  |  | Nfkbib | -0.61 |
| Arfgef1 | -0.47 |  |  |  |  | Hsd11b1 | -0.61 |
| Dnajb4 | -0.47 |  |  |  |  | Acvr2a | -0.61 |
| Tox4 | -0.47 |  |  |  |  | Clip1 | -0.61 |
| Hadha | -0.47 |  |  |  |  | Bach1 | -0.61 |
| Smarcd3 | -0.46 |  |  |  |  | Cirbp | -0.61 |
| Zfp91 | -0.46 |  |  |  |  | Drg2 | -0.61 |
| Nampt | -0.46 |  |  |  |  | Plvap | -0.61 |
| Smtnl2 | -0.46 |  |  |  |  | Hs6st1 | -0.61 |
| Ate1 | -0.46 |  |  |  |  | Polrmt | -0.61 |
| Il6st | -0.46 |  |  |  |  | Timp3 | -0.61 |
| Vti1b | -0.46 |  |  |  |  | Prkab2 | -0.61 |
| Ube2z | -0.45 |  |  |  |  | Jmy | -0.61 |
| Adipor2 | -0.45 |  |  |  |  | Zfp652 | -0.60 |
| Trabd | -0.45 |  |  |  |  | Pfdn6 | -0.60 |
| Slc49a4 | -0.45 |  |  |  |  | Scrn3 | -0.60 |
| Ubr4 | -0.45 |  |  |  |  | Fam120b | -0.60 |
| Chmp7 | -0.45 |  |  |  |  | Dst | -0.60 |
| Ppp6r1 | -0.45 |  |  |  |  | Drap1 | -0.60 |
| Vps52 | -0.45 |  |  |  |  | Pik3r1 | -0.60 |
| Fkbp3 | -0.45 |  |  |  |  | Ptpn21 | -0.60 |
| Sbf1 | -0.45 |  |  |  |  | Fbxo6 | -0.60 |
| Ube2g2 | -0.45 |  |  |  |  | Ivns1abp | -0.60 |
| Slc6a8 | -0.45 |  |  |  |  | Ube2q2 | -0.60 |
| Actn2 | -0.44 |  |  |  |  | Pkdcc | -0.59 |
| Dusp3 | -0.44 |  |  |  |  | Rpf2 | -0.59 |
| Slc35e4 | -0.44 |  |  |  |  | Atpaf1 | -0.59 |
| Ppp2r2d | -0.44 |  |  |  |  | Rbsn | -0.59 |
| Dcaf5 | -0.44 |  |  |  |  | Brcc3 | -0.59 |
| Rpl22l1 | -0.44 |  |  |  |  | Ergic2 | -0.59 |
| Atp2a1 | -0.44 |  |  |  |  | Lactb2 | -0.59 |
| Specc1l | -0.44 |  |  |  |  | Cnot11 | -0.59 |
| Ric8b | -0.44 |  |  |  |  | Utp11 | -0.59 |
| Ppm1l | -0.43 |  |  |  |  | Popdc3 | -0.59 |
| Crispld2 | -0.43 |  |  |  |  | Dda1 | -0.59 |
| Adipor1 | -0.43 |  |  |  |  | Tmpo | -0.59 |
| Sqstm1 | -0.43 |  |  |  |  | Pold2 | -0.59 |
| Rnf10 | -0.43 |  |  |  |  | Mthfd1 | -0.58 |
| Stt3b | -0.43 |  |  |  |  | Gtf2b | -0.58 |
| Dffa | -0.43 |  |  |  |  | Golga5 | -0.58 |
| Zfyve9 | -0.43 |  |  |  |  | Phospho2 | -0.58 |
| Prpf8 | -0.43 |  |  |  |  | Usp47 | -0.58 |
| Cryab | -0.43 |  |  |  |  | Psmc2 | -0.58 |
| Esrra | -0.43 |  |  |  |  | Eef1akmt1 | -0.58 |
| Mgst3 | -0.43 |  |  |  |  | Ptp4a3 | -0.58 |
| Kpna4 | -0.43 |  |  |  |  | Blcap | -0.58 |
| Eloa | -0.43 |  |  |  |  | Gaa | -0.58 |
| Fyttd1 | -0.43 |  |  |  |  | Rbm33 | -0.58 |
| Mapkapk2 | -0.43 |  |  |  |  | Selenow | -0.58 |
| Prune1 | -0.43 |  |  |  |  | Csde1 | -0.58 |
| Plau | -0.42 |  |  |  |  | Mark4 | -0.58 |
| Erbin | -0.42 |  |  |  |  | Snrnp200 | -0.58 |
| Ubqln4 | -0.42 |  |  |  |  | Rad23b | -0.57 |
| Fabp3 | -0.42 |  |  |  |  | Stt3b | -0.57 |
| Hspb6 | -0.42 |  |  |  |  | Ppp2r5b | -0.57 |
| Xirp2 | -0.42 |  |  |  |  | B3galnt2 | -0.57 |
| Tcp1 | -0.42 |  |  |  |  | Asrgl1 | -0.57 |
| Epn1 | -0.42 |  |  |  |  | Cipc | -0.57 |
| Tgm2 | -0.41 |  |  |  |  | Rcc1l | -0.57 |
| Abcf3 | -0.41 |  |  |  |  | Prkcq | -0.57 |
| Ppm1b | -0.41 |  |  |  |  | Ptpn11 | -0.57 |
| Clcn1 | -0.41 |  |  |  |  | Sorbs3 | -0.57 |
| Hspa5 | -0.41 |  |  |  |  | Furin | -0.57 |
| Dlg1 | -0.41 |  |  |  |  | Krr1 | -0.57 |
| Suds3 | -0.41 |  |  |  |  | Gars | -0.57 |
| Mfn1 | -0.41 |  |  |  |  | Stau2 | -0.57 |
| Ndrg2 | -0.41 |  |  |  |  | Rrp9 | -0.57 |
| Gid4 | -0.41 |  |  |  |  | Ccdc91 | -0.57 |
| Gnpat | -0.41 |  |  |  |  | Herpud1 | -0.57 |
| Tns1 | -0.41 |  |  |  |  | Camk2b | -0.57 |
| Cltb | -0.41 |  |  |  |  | Zscan21 | -0.56 |
| Dnaja3 | -0.41 |  |  |  |  | Eif1ad | -0.56 |
| Atp1a1 | -0.41 |  |  |  |  | Dusp13 | -0.56 |
| Rrn3 | -0.40 |  |  |  |  | Sar1b | -0.56 |
| Mbd3 | -0.40 |  |  |  |  | Actr8 | -0.56 |
| Cdv3 | -0.40 |  |  |  |  | Vezf1 | -0.56 |
| Crk | -0.40 |  |  |  |  | Akirin1 | -0.56 |
| Actr1a | -0.40 |  |  |  |  | Fiz1 | -0.56 |
| Cd99l2 | -0.40 |  |  |  |  | Phka1 | -0.56 |
| Etfa | -0.40 |  |  |  |  | Map2k7 | -0.56 |
| Psme4 | -0.40 |  |  |  |  | Eed | -0.56 |
| Ddx24 | -0.40 |  |  |  |  | Fam214b | -0.56 |
| Coq9 | -0.39 |  |  |  |  | Lrrc59 | -0.56 |
| Trak1 | -0.39 |  |  |  |  | Lonp2 | -0.56 |
| Msrb3 | -0.39 |  |  |  |  | Anxa7 | -0.56 |
| Hdlbp | -0.39 |  |  |  |  | Mapk14 | -0.56 |
| Ccdc91 | -0.39 |  |  |  |  | Erbin | -0.56 |
| Prmt1 | -0.39 |  |  |  |  | Inpp5a | -0.56 |
| Mfn2 | -0.39 |  |  |  |  | Nup85 | -0.56 |
| Dyrk1a | -0.39 |  |  |  |  | Foxk2 | -0.56 |
| Akap1 | -0.38 |  |  |  |  | 0610009B22Rik | -0.55 |
| Bag6 | -0.38 |  |  |  |  | Ppip5k1 | -0.55 |
| Ipo7 | -0.38 |  |  |  |  | Supt6 | -0.55 |
| Metap1 | -0.38 |  |  |  |  | Cobl | -0.55 |
| Tufm | -0.38 |  |  |  |  | Fbxo31 | -0.55 |
| Csde1 | -0.38 |  |  |  |  | Rxylt1 | -0.55 |
| Nap1l4 | -0.38 |  |  |  |  | Cul1 | -0.55 |
| Coro6 | -0.38 |  |  |  |  | Naa50 | -0.55 |
| Flii | -0.37 |  |  |  |  | Nup98 | -0.55 |
| Cul5 | -0.37 |  |  |  |  | Nop16 | -0.55 |
| Rad23b | -0.37 |  |  |  |  | Klc2 | -0.55 |
| Mrps18a | -0.37 |  |  |  |  | Eps15 | -0.55 |
| Naa50 | -0.37 |  |  |  |  | Tbrg1 | -0.55 |
| Zfand3 | -0.37 |  |  |  |  | Synpo | -0.55 |
| Kcmf1 | -0.37 |  |  |  |  | Hnrnpab | -0.55 |
| Ppm1a | -0.36 |  |  |  |  | Pdlim3 | -0.55 |
| 4930453N24Rik | -0.36 |  |  |  |  | Hspbp1 | -0.55 |
| Trip12 | -0.36 |  |  |  |  | Nek7 | -0.55 |
| Ddb1 | -0.35 |  |  |  |  | Smarcd3 | -0.55 |
| Eif4g1 | -0.35 |  |  |  |  | Dnajb2 | -0.55 |
| Acadm | -0.35 |  |  |  |  | Pygm | -0.55 |
| Cd151 | -0.34 |  |  |  |  | Trappc10 | -0.55 |
| Setd3 | -0.34 |  |  |  |  | Golga4 | -0.55 |
| Sars | -0.34 |  |  |  |  | Snta1 | -0.55 |
| Arpc5l | -0.34 |  |  |  |  | Gsk3a | -0.55 |
| Alkbh5 | -0.34 |  |  |  |  | Ppm1g | -0.55 |
| Ipo5 | -0.34 |  |  |  |  | Jagn1 | -0.54 |
| Ahsa1 | -0.33 |  |  |  |  | Cbfb | -0.54 |
| Pafah1b1 | -0.33 |  |  |  |  | Zbtb7b | -0.54 |
| Marchf6 | -0.33 |  |  |  |  | Larp1 | -0.54 |
| Fastk | -0.33 |  |  |  |  | Gpr107 | -0.54 |
| Hspd1 | -0.33 |  |  |  |  | Sars | -0.54 |
| Psmd1 | -0.33 |  |  |  |  | Srsf2 | -0.54 |
| Atf4 | -0.32 |  |  |  |  | Mrpl55 | -0.54 |
| Prdx1 | -0.32 |  |  |  |  | Fxr2 | -0.54 |
| Wwp1 | -0.32 |  |  |  |  | Spcs3 | -0.54 |
| Amfr | -0.32 |  |  |  |  | H6pd | -0.54 |
| Arcn1 | -0.30 |  |  |  |  | Ctps | -0.54 |
| Ywhag | -0.30 |  |  |  |  | Hectd1 | -0.54 |
| Bag3 | -0.30 |  |  |  |  | Tfb2m | -0.54 |
| Egln1 | -0.30 |  |  |  |  | Atp6v1h | -0.54 |
| Epdr1 | -0.29 |  |  |  |  | Slc35a4 | -0.54 |
| Phkb | -0.29 |  |  |  |  | Zpr1 | -0.54 |
| Psap | -0.29 |  |  |  |  | Dnajc21 | -0.54 |
| Coq10a | -0.28 |  |  |  |  | Malsu1 | -0.54 |
| Hdgf | -0.28 |  |  |  |  | Wdtc1 | -0.54 |
| Cul3 | -0.26 |  |  |  |  | Zfp91 | -0.54 |
| Rpl17 | 0.26 |  |  |  |  | Cops3 | -0.54 |
| Itm2b | 0.28 |  |  |  |  | Sap18 | -0.53 |
| Cox6c | 0.29 |  |  |  |  | Ell2 | -0.53 |
| Ndufa2 | 0.30 |  |  |  |  | Gtpbp4 | -0.53 |
| Uqcrq | 0.30 |  |  |  |  | Slc2a4 | -0.53 |
| Sfr1 | 0.30 |  |  |  |  | Agl | -0.53 |
| Rps8 | 0.30 |  |  |  |  | Hoxc10 | -0.53 |
| Prdx2 | 0.31 |  |  |  |  | Pdzrn3 | -0.53 |
| Atp5g3 | 0.31 |  |  |  |  | Coro6 | -0.53 |
| Uqcr11 | 0.31 |  |  |  |  | Arih2 | -0.53 |
| Cox7a1 | 0.32 |  |  |  |  | A430005L14Rik | -0.53 |
| Selenok | 0.32 |  |  |  |  | Prpf4 | -0.53 |
| Ndufb1-ps | 0.32 |  |  |  |  | Golph3 | -0.53 |
| Rtn2 | 0.33 |  |  |  |  | Agap3 | -0.53 |
| Rpl26 | 0.33 |  |  |  |  | Vac14 | -0.53 |
| Rhoq | 0.33 |  |  |  |  | Ccdc127 | -0.53 |
| Cops9 | 0.34 |  |  |  |  | Ndrg2 | -0.53 |
| Sem1 | 0.34 |  |  |  |  | Eif2b2 | -0.53 |
| Ak1 | 0.34 |  |  |  |  | Psmd1 | -0.53 |
| Atp5mpl | 0.35 |  |  |  |  | Alas1 | -0.53 |
| Sgcb | 0.35 |  |  |  |  | Cacul1 | -0.52 |
| Cacna2d1 | 0.36 |  |  |  |  | Npepl1 | -0.52 |
| Eno1 | 0.36 |  |  |  |  | Cmya5 | -0.52 |
| Ndufa13 | 0.36 |  |  |  |  | Rfk | -0.52 |
| Ptp4a2 | 0.36 |  |  |  |  | G6pc3 | -0.52 |
| Dhrs7c | 0.37 |  |  |  |  | Ddx1 | -0.52 |
| Rbm39 | 0.38 |  |  |  |  | Nrd1 | -0.52 |
| Mxd4 | 0.38 |  |  |  |  | Bcor | -0.52 |
| Pik3ca | 0.38 |  |  |  |  | Qser1 | -0.52 |
| Wwtr1 | 0.39 |  |  |  |  | Agpat1 | -0.52 |
| Atxn7l3b | 0.39 |  |  |  |  | Dvl3 | -0.52 |
| Map1lc3a | 0.39 |  |  |  |  | Farsa | -0.52 |
| Pdia3 | 0.40 |  |  |  |  | Insr | -0.52 |
| Nsa2 | 0.40 |  |  |  |  | Usp38 | -0.51 |
| Gpd2 | 0.40 |  |  |  |  | Usp10 | -0.51 |
| Lsp1 | 0.40 |  |  |  |  | Hax1 | -0.51 |
| Rbms1 | 0.40 |  |  |  |  | Ubl7 | -0.51 |
| Pdgfrb | 0.41 |  |  |  |  | Dennd4b | -0.51 |
| Atp5e | 0.41 |  |  |  |  | Bccip | -0.51 |
| Ap2s1 | 0.41 |  |  |  |  | Trappc8 | -0.51 |
| Foxn3 | 0.41 |  |  |  |  | Gemin7 | -0.51 |
| Apobec2 | 0.41 |  |  |  |  | Dnajb5 | -0.51 |
| Casq2 | 0.41 |  |  |  |  | Prpf8 | -0.51 |
| Ndufa1 | 0.41 |  |  |  |  | Kpna6 | -0.51 |
| Acta1 | 0.41 |  |  |  |  | Stip1 | -0.51 |
| Naa15 | 0.42 |  |  |  |  | Arl8a | -0.51 |
| Arf4 | 0.42 |  |  |  |  | Pgam5 | -0.51 |
| Atp1b3 | 0.42 |  |  |  |  | Foxj3 | -0.50 |
| Cmpk1 | 0.42 |  |  |  |  | Ckm | -0.50 |
| Tmcc3 | 0.42 |  |  |  |  | Adipor1 | -0.50 |
| Ank3 | 0.42 |  |  |  |  | Gmps | -0.50 |
| Tnrc6a | 0.42 |  |  |  |  | Nxn | -0.50 |
| Arf5 | 0.42 |  |  |  |  | Pef1 | -0.50 |
| Fxyd1 | 0.42 |  |  |  |  | Suox | -0.50 |
| Pcolce | 0.43 |  |  |  |  | Eif4a1 | -0.50 |
| Ndufc1 | 0.43 |  |  |  |  | Tollip | -0.50 |
| Cald1 | 0.43 |  |  |  |  | Mgst3 | -0.50 |
| Dpt | 0.43 |  |  |  |  | Eri3 | -0.50 |
| Cnot6 | 0.43 |  |  |  |  | Crnkl1 | -0.50 |
| Gapdh | 0.43 |  |  |  |  | Kctd15 | -0.50 |
| Snrpe | 0.44 |  |  |  |  | Gatc | -0.50 |
| Cnn3 | 0.44 |  |  |  |  | Tex2 | -0.50 |
| Aldoa | 0.44 |  |  |  |  | Aco2 | -0.50 |
| 1810058I24Rik | 0.44 |  |  |  |  | Samd8 | -0.49 |
| Abhd17b | 0.44 |  |  |  |  | Ppm1a | -0.49 |
| Purb | 0.44 |  |  |  |  | Trappc6b | -0.49 |
| Ttc28 | 0.44 |  |  |  |  | Nob1 | -0.49 |
| Ppp1r12a | 0.44 |  |  |  |  | Heatr3 | -0.49 |
| Ptma | 0.45 |  |  |  |  | Rrn3 | -0.49 |
| Tpm1 | 0.45 |  |  |  |  | Rab35 | -0.49 |
| Asph | 0.45 |  |  |  |  | Bap1 | -0.49 |
| Cfl1 | 0.45 |  |  |  |  | Tcp1 | -0.49 |
| Park7 | 0.45 |  |  |  |  | Nudt9 | -0.49 |
| Arid4b | 0.46 |  |  |  |  | Fam104a | -0.49 |
| Ift20 | 0.46 |  |  |  |  | Phip | -0.49 |
| H3f3a | 0.46 |  |  |  |  | Cnbp | -0.49 |
| Dusp11 | 0.46 |  |  |  |  | Mlycd | -0.49 |
| Cbx1 | 0.46 |  |  |  |  | Morf4l1 | -0.49 |
| Smc6 | 0.46 |  |  |  |  | Ccz1 | -0.49 |
| Cd9 | 0.46 |  |  |  |  | Mindy1 | -0.49 |
| Maged1 | 0.46 |  |  |  |  | Syncrip | -0.49 |
| Irak1 | 0.46 |  |  |  |  | Stk11 | -0.49 |
| Ccnl2 | 0.46 |  |  |  |  | Polr2a | -0.49 |
| Cisd3 | 0.47 |  |  |  |  | Mtln | -0.49 |
| Arid2 | 0.47 |  |  |  |  | Mrpl50 | -0.48 |
| Atp6v1g1 | 0.47 |  |  |  |  | Mef2d | -0.48 |
| Cdh5 | 0.47 |  |  |  |  | Mrps26 | -0.48 |
| Wfdc1 | 0.48 |  |  |  |  | Impdh2 | -0.48 |
| Rbpms | 0.48 |  |  |  |  | Prps1 | -0.48 |
| Tpm2 | 0.48 |  |  |  |  | Sms | -0.48 |
| Cd93 | 0.48 |  |  |  |  | Ranbp3 | -0.48 |
| Luc7l3 | 0.48 |  |  |  |  | Wbp2 | -0.48 |
| Ccar1 | 0.48 |  |  |  |  | Cltb | -0.48 |
| Zfhx3 | 0.48 |  |  |  |  | Cebpg | -0.48 |
| C1qtnf9 | 0.49 |  |  |  |  | Cct6a | -0.48 |
| Ramp1 | 0.49 |  |  |  |  | Psmd11 | -0.48 |
| Cnot4 | 0.49 |  |  |  |  | Rnf220 | -0.48 |
| Idh1 | 0.49 |  |  |  |  | Atad3a | -0.48 |
| Sh3bgrl | 0.49 |  |  |  |  | Gtf2a1 | -0.48 |
| Cetn2 | 0.49 |  |  |  |  | Rnf115 | -0.48 |
| Nav1 | 0.49 |  |  |  |  | Arfgef1 | -0.48 |
| Rbm25 | 0.49 |  |  |  |  | Ate1 | -0.48 |
| Klhl31 | 0.50 |  |  |  |  | Gphn | -0.48 |
| Lamb1 | 0.50 |  |  |  |  | Sympk | -0.47 |
| Nmt2 | 0.50 |  |  |  |  | Impad1 | -0.47 |
| Exd2 | 0.50 |  |  |  |  | Hdgf | -0.47 |
| Ap1s2 | 0.50 |  |  |  |  | Prkacb | -0.47 |
| Cib2 | 0.50 |  |  |  |  | Slc38a4 | -0.47 |
| Egfl7 | 0.50 |  |  |  |  | Larp4 | -0.47 |
| Mphosph8 | 0.50 |  |  |  |  | Bin1 | -0.47 |
| Cav1 | 0.51 |  |  |  |  | Eif4a3 | -0.47 |
| Ndufaf5 | 0.51 |  |  |  |  | Svil | -0.47 |
| Bicc1 | 0.51 |  |  |  |  | Ipo7 | -0.47 |
| Hhatl | 0.51 |  |  |  |  | Mrto4 | -0.47 |
| Ganc | 0.52 |  |  |  |  | Asna1 | -0.46 |
| Ttn | 0.52 |  |  |  |  | Tom1l2 | -0.46 |
| Srek1 | 0.52 |  |  |  |  | Ttc33 | -0.46 |
| Pdlim7 | 0.52 |  |  |  |  | Elob | -0.46 |
| Naa10 | 0.52 |  |  |  |  | Des | -0.46 |
| Pnisr | 0.53 |  |  |  |  | Usp13 | -0.46 |
| Auts2 | 0.53 |  |  |  |  | Gnpat | -0.46 |
| Cenpx | 0.53 |  |  |  |  | Tfam | -0.46 |
| Larp4b | 0.53 |  |  |  |  | Rraga | -0.46 |
| Arpc5 | 0.53 |  |  |  |  | Osgep | -0.46 |
| Serpinf1 | 0.53 |  |  |  |  | Ubap2l | -0.46 |
| Col6a1 | 0.53 |  |  |  |  | Uspl1 | -0.46 |
| Bmp1 | 0.54 |  |  |  |  | Nup88 | -0.46 |
| S100a10 | 0.54 |  |  |  |  | Farsb | -0.46 |
| Ppp3ca | 0.54 |  |  |  |  | Dcun1d2 | -0.46 |
| Kdm5a | 0.54 |  |  |  |  | Tbx15 | -0.46 |
| Rsrp1 | 0.54 |  |  |  |  | Ppp2r2d | -0.46 |
| Gask1b | 0.54 |  |  |  |  | Unc45b | -0.46 |
| D1Ertd622e | 0.54 |  |  |  |  | Ilrun | -0.46 |
| Nras | 0.54 |  |  |  |  | Tab2 | -0.46 |
| Cavin3 | 0.54 |  |  |  |  | Zfand3 | -0.46 |
| Atp5o | 0.54 |  |  |  |  | Smad4 | -0.46 |
| Gnaq | 0.55 |  |  |  |  | Etf1 | -0.46 |
| Col15a1 | 0.55 |  |  |  |  | Arhgap10 | -0.45 |
| Pura | 0.55 |  |  |  |  | Prkaa2 | -0.45 |
| Kdelr2 | 0.55 |  |  |  |  | Hcfc1 | -0.45 |
| Pabpc1 | 0.55 |  |  |  |  | Rheb | -0.45 |
| Atrx | 0.55 |  |  |  |  | Snx13 | -0.45 |
| Nid1 | 0.56 |  |  |  |  | Cgrrf1 | -0.45 |
| Tceal8 | 0.56 |  |  |  |  | Creb3 | -0.45 |
| Hspb3 | 0.56 |  |  |  |  | Rnf6 | -0.45 |
| Gng11 | 0.56 |  |  |  |  | Tma7 | -0.45 |
| Ndn | 0.56 |  |  |  |  | Gps1 | -0.45 |
| Pgam2 | 0.56 |  |  |  |  | Paics | -0.45 |
| Snrnp27 | 0.56 |  |  |  |  | Rnmt | -0.45 |
| Ssc5d | 0.56 |  |  |  |  | Psmb7 | -0.45 |
| Scand1 | 0.57 |  |  |  |  | Hnrnpd | -0.45 |
| Mcam | 0.57 |  |  |  |  | Zfp106 | -0.45 |
| Cav2 | 0.57 |  |  |  |  | Ddx23 | -0.45 |
| Micu3 | 0.57 |  |  |  |  | Vps37a | -0.44 |
| Hoxc9 | 0.57 |  |  |  |  | Mipep | -0.44 |
| Nabp1 | 0.58 |  |  |  |  | Cdc123 | -0.44 |
| Rhoj | 0.58 |  |  |  |  | Mettl22 | -0.44 |
| Thoc2 | 0.58 |  |  |  |  | Eif4g1 | -0.44 |
| Tjp1 | 0.58 |  |  |  |  | Mrps18a | -0.44 |
| Fam133b | 0.58 |  |  |  |  | Bag6 | -0.44 |
| Angptl2 | 0.58 |  |  |  |  | Ttc4 | -0.44 |
| Mpv17 | 0.58 |  |  |  |  | Ubl3 | -0.44 |
| Wsb1 | 0.59 |  |  |  |  | Specc1l | -0.44 |
| Zc3h7a | 0.59 |  |  |  |  | Polr2c | -0.44 |
| Svbp | 0.59 |  |  |  |  | Pdk2 | -0.44 |
| Dap | 0.59 |  |  |  |  | Vti1b | -0.44 |
| Cavin2 | 0.59 |  |  |  |  | Alkbh5 | -0.44 |
| Antxr1 | 0.59 |  |  |  |  | Tigar | -0.44 |
| Fam114a1 | 0.59 |  |  |  |  | Fyttd1 | -0.44 |
| Fabp4 | 0.59 |  |  |  |  | Setd7 | -0.44 |
| Slfn5 | 0.59 |  |  |  |  | Hipk3 | -0.44 |
| Ints6l | 0.60 |  |  |  |  | Pno1 | -0.44 |
| Lpar1 | 0.60 |  |  |  |  | Ubr2 | -0.44 |
| Col6a6 | 0.60 |  |  |  |  | Mkln1 | -0.43 |
| Larp7 | 0.60 |  |  |  |  | Nars | -0.43 |
| Plcb4 | 0.60 |  |  |  |  | Hspa9 | -0.43 |
| Ptms | 0.60 |  |  |  |  | Cul2 | -0.43 |
| Tceal9 | 0.60 |  |  |  |  | Tfg | -0.43 |
| Chrnb1 | 0.60 |  |  |  |  | Kpna4 | -0.43 |
| Ckap4 | 0.60 |  |  |  |  | Ubxn4 | -0.43 |
| Rock1 | 0.61 |  |  |  |  | Ube2j2 | -0.43 |
| S1pr1 | 0.61 |  |  |  |  | Sumo3 | -0.43 |
| Septin4 | 0.62 |  |  |  |  | 4833439L19Rik | -0.43 |
| Brd9 | 0.62 |  |  |  |  | Smpdl3a | -0.43 |
| Ip6k3 | 0.62 |  |  |  |  | Cavin4 | -0.43 |
| Iffo1 | 0.62 |  |  |  |  | H2az1 | -0.43 |
| Ext1 | 0.62 |  |  |  |  | St3gal3 | -0.43 |
| Klhl33 | 0.63 |  |  |  |  | Kctd9 | -0.43 |
| Cmbl | 0.63 |  |  |  |  | Tmem248 | -0.43 |
| Nfib | 0.63 |  |  |  |  | Ppp6r3 | -0.43 |
| Fam49a | 0.63 |  |  |  |  | Prcc | -0.43 |
| Nexn | 0.63 |  |  |  |  | Lbx1 | -0.43 |
| Col4a2 | 0.63 |  |  |  |  | Emg1 | -0.43 |
| Fabp5 | 0.64 |  |  |  |  | Napa | -0.43 |
| Celf2 | 0.64 |  |  |  |  | Adssl1 | -0.43 |
| Adamts10 | 0.64 |  |  |  |  | Saraf | -0.42 |
| Mdfic | 0.64 |  |  |  |  | Riok3 | -0.42 |
| Fitm1 | 0.64 |  |  |  |  | Myo18a | -0.42 |
| Litaf | 0.64 |  |  |  |  | Pak1ip1 | -0.42 |
| Wipf1 | 0.64 |  |  |  |  | L2hgdh | -0.42 |
| Adgrl4 | 0.65 |  |  |  |  | 2310022A10Rik | -0.42 |
| Igfbp7 | 0.65 |  |  |  |  | Zfyve9 | -0.42 |
| Aspn | 0.65 |  |  |  |  | Spr | -0.42 |
| Tcf4 | 0.65 |  |  |  |  | Hikeshi | -0.42 |
| Fam193b | 0.65 |  |  |  |  | Smim10l1 | -0.42 |
| Mmp14 | 0.65 |  |  |  |  | Lap3 | -0.42 |
| Fscn1 | 0.65 |  |  |  |  | Atl2 | -0.42 |
| Dpysl2 | 0.66 |  |  |  |  | Slc49a4 | -0.42 |
| Map4k4 | 0.66 |  |  |  |  | Cul3 | -0.42 |
| Blvrb | 0.66 |  |  |  |  | Eif4a2 | -0.42 |
| 2510002D24Rik | 0.66 |  |  |  |  | Pink1 | -0.42 |
| Hmgn5 | 0.66 |  |  |  |  | Tfeb | -0.42 |
| Kank3 | 0.67 |  |  |  |  | Dctn1 | -0.42 |
| Tmsb4x | 0.67 |  |  |  |  | Nfe2l1 | -0.42 |
| Dock6 | 0.67 |  |  |  |  | Arpc5l | -0.41 |
| Adamts5 | 0.67 |  |  |  |  | Prr33 | -0.41 |
| Pmp22 | 0.68 |  |  |  |  | Immp1l | -0.41 |
| Efna1 | 0.68 |  |  |  |  | Srp68 | -0.41 |
| Deptor | 0.68 |  |  |  |  | Aip | -0.41 |
| Chpf | 0.68 |  |  |  |  | Slc25a46 | -0.41 |
| Apoe | 0.68 |  |  |  |  | Capn1 | -0.41 |
| Xpo6 | 0.68 |  |  |  |  | Dnaja3 | -0.41 |
| Gbp7 | 0.68 |  |  |  |  | Psme4 | -0.41 |
| Ip6k2 | 0.69 |  |  |  |  | Hspa5 | -0.41 |
| Fam92a | 0.69 |  |  |  |  | Akt1s1 | -0.41 |
| Serpinh1 | 0.69 |  |  |  |  | Grpel1 | -0.41 |
| Cbx6 | 0.69 |  |  |  |  | Rac1 | -0.40 |
| Arfip2 | 0.70 |  |  |  |  | Tnni2 | -0.40 |
| Itgb1bp2 | 0.70 |  |  |  |  | Rmnd5a | -0.40 |
| Sat1 | 0.70 |  |  |  |  | Fam53a | -0.40 |
| Rftn1 | 0.70 |  |  |  |  | Bod1 | -0.40 |
| Sytl2 | 0.71 |  |  |  |  | Arl8b | -0.40 |
| Ccdc80 | 0.71 |  |  |  |  | Lrpprc | -0.40 |
| Lrrfip1 | 0.71 |  |  |  |  | Tmem11 | -0.40 |
| Tmem52 | 0.71 |  |  |  |  | Vps4a | -0.40 |
| Zbtb20 | 0.72 |  |  |  |  | Plaat3 | -0.40 |
| Rabep2 | 0.72 |  |  |  |  | Cse1l | -0.40 |
| Lsmem1 | 0.72 |  |  |  |  | Ppp3cb | -0.40 |
| Chrna1 | 0.72 |  |  |  |  | Eif1 | -0.40 |
| Afdn | 0.72 |  |  |  |  | Rpn1 | -0.39 |
| C2cd5 | 0.72 |  |  |  |  | Elp3 | -0.39 |
| Ptprd | 0.72 |  |  |  |  | Hnrnpul1 | -0.39 |
| Plk2 | 0.73 |  |  |  |  | Kpna1 | -0.39 |
| Dtx4 | 0.73 |  |  |  |  | Banf1 | -0.39 |
| Abi3bp | 0.73 |  |  |  |  | Hspa4 | -0.39 |
| Thoc2l | 0.73 |  |  |  |  | Mzt1 | -0.39 |
| Sntb1 | 0.73 |  |  |  |  | Ahsa1 | -0.39 |
| Mndal | 0.73 |  |  |  |  | Anp32e | -0.39 |
| Ggta1 | 0.74 |  |  |  |  | Cog4 | -0.39 |
| Lsm7 | 0.74 |  |  |  |  | Mrps7 | -0.39 |
| Srsf11 | 0.74 |  |  |  |  | Fnta | -0.39 |
| Trim12c | 0.75 |  |  |  |  | Ociad1 | -0.38 |
| Dgkz | 0.75 |  |  |  |  | Csgalnact2 | -0.38 |
| Trim2 | 0.76 |  |  |  |  | Ankrd40 | -0.38 |
| Ptprs | 0.76 |  |  |  |  | Vdac2 | -0.38 |
| Tceal5 | 0.77 |  |  |  |  | Myom1 | -0.38 |
| Dock4 | 0.77 |  |  |  |  | Morf4l2 | -0.38 |
| Nupr1 | 0.77 |  |  |  |  | Zbtb18 | -0.38 |
| Pid1 | 0.77 |  |  |  |  | Tnnt3 | -0.38 |
| Cd59a | 0.77 |  |  |  |  | Herpud2 | -0.38 |
| Pla2g4e | 0.78 |  |  |  |  | Msrb1 | -0.38 |
| Nfia | 0.78 |  |  |  |  | Chrac1 | -0.38 |
| Ankrd10 | 0.78 |  |  |  |  | Ap4s1 | -0.38 |
| Klf5 | 0.78 |  |  |  |  | Rapgef1 | -0.38 |
| Ebf1 | 0.78 |  |  |  |  | Cct2 | -0.38 |
| Ccdc88a | 0.78 |  |  |  |  | Nudt19 | -0.37 |
| Col6a3 | 0.78 |  |  |  |  | Slc25a51 | -0.37 |
| Maged2 | 0.79 |  |  |  |  | Rsl1d1 | -0.37 |
| Adamts2 | 0.79 |  |  |  |  | Ensa | -0.37 |
| Tcf7l2 | 0.81 |  |  |  |  | Nmt1 | -0.37 |
| Mfap5 | 0.81 |  |  |  |  | Yy1 | -0.37 |
| Gadd45a | 0.81 |  |  |  |  | Ube2b | -0.37 |
| Pld2 | 0.81 |  |  |  |  | Fbxl3 | -0.37 |
| Cul7 | 0.82 |  |  |  |  | Dlg1 | -0.37 |
| Pvalb | 0.82 |  |  |  |  | Setd3 | -0.37 |
| Zdhhc20 | 0.82 |  |  |  |  | Rabac1 | -0.36 |
| Tet2 | 0.82 |  |  |  |  | Pfkm | -0.36 |
| Zfp950 | 0.82 |  |  |  |  | Cct8 | -0.36 |
| Ncam1 | 0.84 |  |  |  |  | Kpnb1 | -0.36 |
| Csad | 0.84 |  |  |  |  | Calm3 | -0.36 |
| Col14a1 | 0.84 |  |  |  |  | Yme1l1 | -0.35 |
| Dapk1 | 0.84 |  |  |  |  | Fto | -0.35 |
| Smc4 | 0.84 |  |  |  |  | Gyg | -0.35 |
| Sesn3 | 0.84 |  |  |  |  | Amotl1 | -0.35 |
| Mafa | 0.84 |  |  |  |  | Cdc34 | -0.35 |
| Car3 | 0.85 |  |  |  |  | Ppp2ca | -0.35 |
| Loxl2 | 0.86 |  |  |  |  | Strn3 | -0.35 |
| Emid1 | 0.86 |  |  |  |  | Six1 | -0.35 |
| 4930402H24Rik | 0.87 |  |  |  |  | Cisd1 | -0.34 |
| Casq1 | 0.87 |  |  |  |  | Amfr | -0.34 |
| Pck2 | 0.88 |  |  |  |  | Kars | -0.34 |
| Trim7 | 0.88 |  |  |  |  | Gstp1 | -0.34 |
| Sash1 | 0.89 |  |  |  |  | Mypn | -0.34 |
| Myl1 | 0.89 |  |  |  |  | Sgta | -0.34 |
| Neu2 | 0.89 |  |  |  |  | Cwc15 | -0.34 |
| Carns1 | 0.90 |  |  |  |  | Cops5 | -0.34 |
| Sult1a1 | 0.90 |  |  |  |  | Hdlbp | -0.34 |
| Car4 | 0.90 |  |  |  |  | Gorasp2 | -0.34 |
| C1qtnf2 | 0.90 |  |  |  |  | Arcn1 | -0.34 |
| Sap30 | 0.91 |  |  |  |  | Vapa | -0.34 |
| Trdn | 0.91 |  |  |  |  | Ran | -0.34 |
| Lrp2bp | 0.91 |  |  |  |  | Prdx1 | -0.33 |
| Chodl | 0.92 |  |  |  |  | Ktn1 | -0.33 |
| Col5a3 | 0.92 |  |  |  |  | Acyp2 | -0.33 |
| Shox2 | 0.92 |  |  |  |  | Ybx1 | -0.33 |
| Obsl1 | 0.92 |  |  |  |  | Mfn2 | -0.33 |
| Krt10 | 0.93 |  |  |  |  | Ppp2r1a | -0.33 |
| Rbms3 | 0.93 |  |  |  |  | Arf1 | -0.32 |
| Dusp6 | 0.93 |  |  |  |  | Ipo5 | -0.32 |
| Basp1 | 0.94 |  |  |  |  | Eif5a | -0.32 |
| Sec14l5 | 0.94 |  |  |  |  | Csnk1a1 | -0.31 |
| Bmf | 0.94 |  |  |  |  | Ddb1 | -0.31 |
| Ncoa7 | 0.95 |  |  |  |  | Pafah1b1 | -0.31 |
| Grrp1 | 0.96 |  |  |  |  | Hspd1 | -0.31 |
| Mthfd2l | 0.96 |  |  |  |  | Hsp90ab1 | -0.31 |
| Lrtm1 | 0.97 |  |  |  |  | Egln1 | -0.31 |
| Hpgd | 0.97 |  |  |  |  | Acat1 | -0.31 |
| Agtr1a | 0.97 |  |  |  |  | Rps20 | -0.31 |
| Als2cl | 0.97 |  |  |  |  | Rps27l | -0.31 |
| Gramd1b | 0.98 |  |  |  |  | Nap1l4 | -0.30 |
| Dkk2 | 0.98 |  |  |  |  | Rab10 | -0.30 |
| Kcna2 | 0.98 |  |  |  |  | Tmod4 | -0.30 |
| Palld | 0.98 |  |  |  |  | Psmd2 | -0.30 |
| Plp1 | 0.99 |  |  |  |  | Fkbp4 | -0.29 |
| Col4a1 | 0.99 |  |  |  |  | Vdac3 | -0.29 |
| Hmgn3 | 0.99 |  |  |  |  | Rps25 | -0.29 |
| Fstl1 | 0.99 |  |  |  |  | Ywhae | -0.28 |
| Snn | 1.00 |  |  |  |  | Cyc1 | 0.27 |
| Smad1 | 1.00 |  |  |  |  | Uqcrfs1 | 0.30 |
| Col5a1 | 1.01 |  |  |  |  | Pdhb | 0.31 |
| Prkag3 | 1.01 |  |  |  |  | Cox6c | 0.33 |
| Mdga1 | 1.03 |  |  |  |  | Hrc | 0.33 |
| Mpz | 1.04 |  |  |  |  | Gnai2 | 0.34 |
| Plod2 | 1.06 |  |  |  |  | Mbnl2 | 0.36 |
| Cnrip1 | 1.06 |  |  |  |  | Sptbn1 | 0.37 |
| Eln | 1.07 |  |  |  |  | Itm2b | 0.38 |
| Creb3l1 | 1.07 |  |  |  |  | Sspn | 0.38 |
| Col5a2 | 1.07 |  |  |  |  | Got2 | 0.39 |
| Ppp1r9a | 1.08 |  |  |  |  | Apobec2 | 0.39 |
| Lratd2 | 1.08 |  |  |  |  | Tgfbr2 | 0.40 |
| Marcks | 1.08 |  |  |  |  | Cd47 | 0.40 |
| Sema6a | 1.08 |  |  |  |  | Mtch1 | 0.40 |
| Trp53i11 | 1.09 |  |  |  |  | Ak3 | 0.40 |
| Dock10 | 1.09 |  |  |  |  | Prdx2 | 0.41 |
| B3glct | 1.10 |  |  |  |  | Cyb5r3 | 0.41 |
| Hs6st2 | 1.10 |  |  |  |  | Srsf11 | 0.41 |
| Fst | 1.10 |  |  |  |  | Cald1 | 0.41 |
| Col16a1 | 1.10 |  |  |  |  | Gnb1 | 0.41 |
| Cldn5 | 1.10 |  |  |  |  | Ctsb | 0.41 |
| Zfp40 | 1.10 |  |  |  |  | Rap1b | 0.41 |
| Tmsb10 | 1.11 |  |  |  |  | Cd81 | 0.42 |
| Grb10 | 1.11 |  |  |  |  | Thoc2 | 0.42 |
| Pxdn | 1.11 |  |  |  |  | Ly6e | 0.42 |
| Mdk | 1.11 |  |  |  |  | Ltbp4 | 0.43 |
| Hacd1 | 1.13 |  |  |  |  | Tek | 0.43 |
| Lpin3 | 1.15 |  |  |  |  | Cd9 | 0.43 |
| Igf1 | 1.15 |  |  |  |  | Mlxipl | 0.43 |
| Col27a1 | 1.15 |  |  |  |  | Atp1a2 | 0.44 |
| Plekhg1 | 1.17 |  |  |  |  | Acadl | 0.44 |
| Hbegf | 1.18 |  |  |  |  | Ehd2 | 0.44 |
| Ppic | 1.19 |  |  |  |  | Atrx | 0.44 |
| Phf11d | 1.19 |  |  |  |  | Snrk | 0.44 |
| Fxyd2 | 1.19 |  |  |  |  | Ano6 | 0.45 |
| Klhl13 | 1.19 |  |  |  |  | Crip2 | 0.45 |
| Ift27 | 1.19 |  |  |  |  | Klf6 | 0.45 |
| Lpar6 | 1.20 |  |  |  |  | Hadh | 0.45 |
| Pdzd7 | 1.20 |  |  |  |  | Ccni | 0.45 |
| Ptn | 1.20 |  |  |  |  | Clstn1 | 0.45 |
| Igfbp3 | 1.21 |  |  |  |  | Rbms1 | 0.46 |
| Plcb1 | 1.21 |  |  |  |  | Tshz2 | 0.46 |
| Cercam | 1.21 |  |  |  |  | Mxd4 | 0.46 |
| Rsad2 | 1.22 |  |  |  |  | Ndufab1 | 0.46 |
| Rnf144a | 1.22 |  |  |  |  | Lpl | 0.46 |
| Mylpf | 1.22 |  |  |  |  | Dusp11 | 0.46 |
| Lrch2 | 1.23 |  |  |  |  | Deptor | 0.46 |
| Matn4 | 1.26 |  |  |  |  | Lamp2 | 0.47 |
| Rflnb | 1.27 |  |  |  |  | Rsrp1 | 0.47 |
| Aplnr | 1.27 |  |  |  |  | Atp1b2 | 0.47 |
| Tnmd | 1.28 |  |  |  |  | Map4k3 | 0.47 |
| Col11a1 | 1.28 |  |  |  |  | Tceal9 | 0.48 |
| Adamts12 | 1.29 |  |  |  |  | H3f3a | 0.48 |
| Lox | 1.29 |  |  |  |  | Iqgap1 | 0.48 |
| Mfap2 | 1.29 |  |  |  |  | Synpo2l | 0.48 |
| Spon2 | 1.31 |  |  |  |  | Septin8 | 0.48 |
| Abcc8 | 1.31 |  |  |  |  | Thap3 | 0.48 |
| Cilp2 | 1.33 |  |  |  |  | Ddx17 | 0.48 |
| Zfp185 | 1.35 |  |  |  |  | Fam189a2 | 0.48 |
| Arpp21 | 1.36 |  |  |  |  | Cnot6l | 0.49 |
| Lpar4 | 1.36 |  |  |  |  | Actb | 0.49 |
| Fbxl22 | 1.37 |  |  |  |  | Larp4b | 0.49 |
| Cacnb3 | 1.37 |  |  |  |  | Nr1d2 | 0.49 |
| Krt222 | 1.38 |  |  |  |  | Btbd3 | 0.49 |
| Zfp979 | 1.38 |  |  |  |  | Adam10 | 0.49 |
| Cd28 | 1.39 |  |  |  |  | Rcn3 | 0.50 |
| Vstm4 | 1.40 |  |  |  |  | Klf3 | 0.50 |
| Itm2a | 1.42 |  |  |  |  | Epb41l2 | 0.50 |
| Col4a5 | 1.44 |  |  |  |  | Trio | 0.50 |
| C1qtnf6 | 1.44 |  |  |  |  | Ddr2 | 0.50 |
| Calcr | 1.46 |  |  |  |  | Pabpc1 | 0.50 |
| Mamstr | 1.51 |  |  |  |  | Ccnt2 | 0.50 |
| Col12a1 | 1.53 |  |  |  |  | Nes | 0.51 |
| Sh2d6 | 1.53 |  |  |  |  | Tppp3 | 0.51 |
| Ccn4 | 1.54 |  |  |  |  | Fcgrt | 0.51 |
| Nrep | 1.54 |  |  |  |  | Hmcn2 | 0.51 |
| Cdh11 | 1.56 |  |  |  |  | Slc29a1 | 0.51 |
| Pi15 | 1.57 |  |  |  |  | Dpt | 0.51 |
| Wif1 | 1.58 |  |  |  |  | Idh1 | 0.51 |
| Pcdh17 | 1.60 |  |  |  |  | Fam129a | 0.51 |
| Postn | 1.61 |  |  |  |  | Ankrd12 | 0.52 |
| Ecrg4 | 1.63 |  |  |  |  | Prelid1 | 0.52 |
| Sparc | 1.65 |  |  |  |  | Clip4 | 0.52 |
| Col11a2 | 1.66 |  |  |  |  | Cdh13 | 0.52 |
| Sox4 | 1.68 |  |  |  |  | Ldhb | 0.52 |
| Myog | 1.69 |  |  |  |  | Afap1l1 | 0.53 |
| A930003A15Rik | 1.71 |  |  |  |  | Lims1 | 0.53 |
| Mfap4 | 1.71 |  |  |  |  | Plcg1 | 0.53 |
| Megf10 | 1.71 |  |  |  |  | C1s1 | 0.53 |
| Col3a1 | 1.73 |  |  |  |  | Prex2 | 0.53 |
| Rgs6 | 1.76 |  |  |  |  | Arhgef2 | 0.53 |
| Plagl1 | 1.80 |  |  |  |  | Map3k11 | 0.53 |
| Zdbf2 | 1.81 |  |  |  |  | Tspan13 | 0.53 |
| Col2a1 | 1.83 |  |  |  |  | Cnot6 | 0.53 |
| Gap43 | 1.85 |  |  |  |  | Mtss1 | 0.54 |
| Col28a1 | 1.87 |  |  |  |  | Serpinf1 | 0.54 |
| Cnbd2 | 1.88 |  |  |  |  | Atp5g3 | 0.54 |
| Kera | 1.88 |  |  |  |  | Atxn7l3b | 0.54 |
| Cdkn1c | 1.89 |  |  |  |  | Sat1 | 0.54 |
| Gamt | 1.89 |  |  |  |  | Rab3il1 | 0.55 |
| Mboat2 | 1.89 |  |  |  |  | Myadm | 0.55 |
| Col1a2 | 1.91 |  |  |  |  | Igdcc4 | 0.55 |
| Mettl21e | 1.92 |  |  |  |  | Hspa12b | 0.55 |
| Dlk1 | 2.00 |  |  |  |  | Myl9 | 0.55 |
| Mybph | 2.01 |  |  |  |  | Ebf1 | 0.55 |
| Zim1 | 2.02 |  |  |  |  | Mgll | 0.55 |
| Peg3 | 2.02 |  |  |  |  | Emp3 | 0.55 |
| Gm4841 | 2.18 |  |  |  |  | Ddah2 | 0.55 |
| Col1a1 | 2.21 |  |  |  |  | Tagln2 | 0.56 |
| Nrk | 2.26 |  |  |  |  | Idh2 | 0.56 |
| Angptl4 | 2.46 |  |  |  |  | Hdac7 | 0.56 |
| Col26a1 | 2.47 |  |  |  |  | Coq7 | 0.56 |
| Ncmap | 2.53 |  |  |  |  | Clec3b | 0.56 |
| Bcat1 | 2.65 |  |  |  |  | Ankrd10 | 0.57 |
| Xlr3b | 2.84 |  |  |  |  | Lamc1 | 0.57 |
| Kcne1l | 3.16 |  |  |  |  | Jund | 0.57 |
| Chrna9 | 3.28 |  |  |  |  | Nras | 0.57 |
| Aldh1a7 | 3.42 |  |  |  |  | Rbfox2 | 0.57 |
| Ddc | 3.75 |  |  |  |  | Septin4 | 0.57 |
| Actc1 | 3.87 |  |  |  |  | Fndc1 | 0.57 |
| Fbn2 | 4.28 |  |  |  |  | Igfbp7 | 0.58 |
| Mymk | 4.57 |  |  |  |  | Sh3d19 | 0.58 |
|  |  |  |  |  |  | Pwwp3a | 0.58 |
|  |  |  |  |  |  | Ccnl2 | 0.58 |
|  |  |  |  |  |  | Elk3 | 0.58 |
|  |  |  |  |  |  | Mapk8ip3 | 0.58 |
|  |  |  |  |  |  | Ramp2 | 0.58 |
|  |  |  |  |  |  | Actn2 | 0.58 |
|  |  |  |  |  |  | Efhd2 | 0.58 |
|  |  |  |  |  |  | Mylpf | 0.58 |
|  |  |  |  |  |  | Parva | 0.58 |
|  |  |  |  |  |  | Entpd2 | 0.58 |
|  |  |  |  |  |  | Rpl3 | 0.58 |
|  |  |  |  |  |  | Gpam | 0.59 |
|  |  |  |  |  |  | Tie1 | 0.59 |
|  |  |  |  |  |  | Leprot | 0.59 |
|  |  |  |  |  |  | Ptpn12 | 0.59 |
|  |  |  |  |  |  | Hspg2 | 0.59 |
|  |  |  |  |  |  | Kif13b | 0.60 |
|  |  |  |  |  |  | Klf13 | 0.60 |
|  |  |  |  |  |  | Casq1 | 0.60 |
|  |  |  |  |  |  | Emilin2 | 0.60 |
|  |  |  |  |  |  | Nfia | 0.60 |
|  |  |  |  |  |  | Rgl1 | 0.60 |
|  |  |  |  |  |  | Srek1 | 0.60 |
|  |  |  |  |  |  | Cnn3 | 0.60 |
|  |  |  |  |  |  | Cfl1 | 0.60 |
|  |  |  |  |  |  | Nrp1 | 0.60 |
|  |  |  |  |  |  | Cavin3 | 0.60 |
|  |  |  |  |  |  | Sash1 | 0.60 |
|  |  |  |  |  |  | Dnmt3a | 0.60 |
|  |  |  |  |  |  | Zfp160 | 0.60 |
|  |  |  |  |  |  | Crtap | 0.60 |
|  |  |  |  |  |  | Cd248 | 0.61 |
|  |  |  |  |  |  | Pcolce | 0.61 |
|  |  |  |  |  |  | Ptms | 0.61 |
|  |  |  |  |  |  | Tia1 | 0.61 |
|  |  |  |  |  |  | Eid1 | 0.61 |
|  |  |  |  |  |  | Fam193b | 0.61 |
|  |  |  |  |  |  | Ssc5d | 0.61 |
|  |  |  |  |  |  | S100a10 | 0.62 |
|  |  |  |  |  |  | Ganc | 0.62 |
|  |  |  |  |  |  | Arpc5 | 0.62 |
|  |  |  |  |  |  | Smad1 | 0.62 |
|  |  |  |  |  |  | Nsmf | 0.62 |
|  |  |  |  |  |  | Spg21 | 0.62 |
|  |  |  |  |  |  | Ckb | 0.62 |
|  |  |  |  |  |  | Dgkz | 0.63 |
|  |  |  |  |  |  | F11r | 0.63 |
|  |  |  |  |  |  | Tpm2 | 0.63 |
|  |  |  |  |  |  | Akap12 | 0.63 |
|  |  |  |  |  |  | Tmcc3 | 0.63 |
|  |  |  |  |  |  | Plxnd1 | 0.63 |
|  |  |  |  |  |  | Nrp2 | 0.63 |
|  |  |  |  |  |  | Rnf213 | 0.63 |
|  |  |  |  |  |  | Arhgef15 | 0.63 |
|  |  |  |  |  |  | Ly6c1 | 0.64 |
|  |  |  |  |  |  | Pan3 | 0.64 |
|  |  |  |  |  |  | Atp1b3 | 0.64 |
|  |  |  |  |  |  | Cilp | 0.64 |
|  |  |  |  |  |  | Cd2ap | 0.64 |
|  |  |  |  |  |  | Evc | 0.64 |
|  |  |  |  |  |  | Adgrf5 | 0.64 |
|  |  |  |  |  |  | Tacc1 | 0.64 |
|  |  |  |  |  |  | Mrtfb | 0.64 |
|  |  |  |  |  |  | Plcd1 | 0.64 |
|  |  |  |  |  |  | Pros1 | 0.64 |
|  |  |  |  |  |  | Esr1 | 0.64 |
|  |  |  |  |  |  | Ache | 0.65 |
|  |  |  |  |  |  | Arhgef25 | 0.65 |
|  |  |  |  |  |  | Map1b | 0.65 |
|  |  |  |  |  |  | Fbln7 | 0.65 |
|  |  |  |  |  |  | Pdgfrb | 0.65 |
|  |  |  |  |  |  | Ramp1 | 0.65 |
|  |  |  |  |  |  | Cers5 | 0.65 |
|  |  |  |  |  |  | Gas7 | 0.65 |
|  |  |  |  |  |  | Arl6ip1 | 0.65 |
|  |  |  |  |  |  | Dock6 | 0.65 |
|  |  |  |  |  |  | Ablim1 | 0.65 |
|  |  |  |  |  |  | Grn | 0.65 |
|  |  |  |  |  |  | Col6a6 | 0.65 |
|  |  |  |  |  |  | Sypl2 | 0.66 |
|  |  |  |  |  |  | Pdgfb | 0.66 |
|  |  |  |  |  |  | Pid1 | 0.66 |
|  |  |  |  |  |  | Myo6 | 0.66 |
|  |  |  |  |  |  | Lims2 | 0.66 |
|  |  |  |  |  |  | Sirpa | 0.66 |
|  |  |  |  |  |  | Lamb1 | 0.66 |
|  |  |  |  |  |  | Ppt1 | 0.66 |
|  |  |  |  |  |  | Tmem47 | 0.66 |
|  |  |  |  |  |  | Mmp2 | 0.66 |
|  |  |  |  |  |  | Nfib | 0.67 |
|  |  |  |  |  |  | Pdcd4 | 0.67 |
|  |  |  |  |  |  | Dtx4 | 0.67 |
|  |  |  |  |  |  | Kank3 | 0.67 |
|  |  |  |  |  |  | Bmp1 | 0.67 |
|  |  |  |  |  |  | Vsir | 0.67 |
|  |  |  |  |  |  | Sdc2 | 0.67 |
|  |  |  |  |  |  | Gucy1b1 | 0.67 |
|  |  |  |  |  |  | Exd2 | 0.67 |
|  |  |  |  |  |  | Vgll2 | 0.67 |
|  |  |  |  |  |  | Maged1 | 0.67 |
|  |  |  |  |  |  | Colgalt2 | 0.67 |
|  |  |  |  |  |  | Mrc1 | 0.67 |
|  |  |  |  |  |  | Litaf | 0.67 |
|  |  |  |  |  |  | Flrt2 | 0.67 |
|  |  |  |  |  |  | Chrnb1 | 0.67 |
|  |  |  |  |  |  | Lmo2 | 0.67 |
|  |  |  |  |  |  | Xpo6 | 0.68 |
|  |  |  |  |  |  | Lsp1 | 0.68 |
|  |  |  |  |  |  | Sp100 | 0.68 |
|  |  |  |  |  |  | Pik3c2b | 0.68 |
|  |  |  |  |  |  | Lama4 | 0.68 |
|  |  |  |  |  |  | Cd34 | 0.68 |
|  |  |  |  |  |  | Pla2g4e | 0.69 |
|  |  |  |  |  |  | Lyz2 | 0.69 |
|  |  |  |  |  |  | Jcad | 0.69 |
|  |  |  |  |  |  | Arhgap29 | 0.69 |
|  |  |  |  |  |  | Itih5 | 0.69 |
|  |  |  |  |  |  | Unc93b1 | 0.69 |
|  |  |  |  |  |  | Tceal8 | 0.69 |
|  |  |  |  |  |  | Eef1a1 | 0.69 |
|  |  |  |  |  |  | C130074G19Rik | 0.69 |
|  |  |  |  |  |  | Ywhaq | 0.69 |
|  |  |  |  |  |  | Hsd17b11 | 0.69 |
|  |  |  |  |  |  | Apoe | 0.69 |
|  |  |  |  |  |  | Oaf | 0.69 |
|  |  |  |  |  |  | Fmnl3 | 0.70 |
|  |  |  |  |  |  | Basp1 | 0.70 |
|  |  |  |  |  |  | Nupr1 | 0.70 |
|  |  |  |  |  |  | Nbl1 | 0.70 |
|  |  |  |  |  |  | Zfp703 | 0.70 |
|  |  |  |  |  |  | Rassf2 | 0.70 |
|  |  |  |  |  |  | Ints6l | 0.70 |
|  |  |  |  |  |  | Adamtsl4 | 0.70 |
|  |  |  |  |  |  | Itga6 | 0.70 |
|  |  |  |  |  |  | Lrrfip1 | 0.71 |
|  |  |  |  |  |  | Actg1 | 0.71 |
|  |  |  |  |  |  | Inpp1 | 0.71 |
|  |  |  |  |  |  | Dab2 | 0.71 |
|  |  |  |  |  |  | Rin2 | 0.71 |
|  |  |  |  |  |  | Chpf | 0.71 |
|  |  |  |  |  |  | Adcy7 | 0.71 |
|  |  |  |  |  |  | Lpar1 | 0.71 |
|  |  |  |  |  |  | Sh3bgrl3 | 0.71 |
|  |  |  |  |  |  | Rnd3 | 0.71 |
|  |  |  |  |  |  | Arpc1b | 0.72 |
|  |  |  |  |  |  | Meox2 | 0.72 |
|  |  |  |  |  |  | Slc38a3 | 0.72 |
|  |  |  |  |  |  | C2cd5 | 0.72 |
|  |  |  |  |  |  | Ufsp1 | 0.72 |
|  |  |  |  |  |  | Steap3 | 0.72 |
|  |  |  |  |  |  | Gng11 | 0.72 |
|  |  |  |  |  |  | Ckmt2 | 0.72 |
|  |  |  |  |  |  | Plcb4 | 0.72 |
|  |  |  |  |  |  | Rbms3 | 0.72 |
|  |  |  |  |  |  | Cdh5 | 0.72 |
|  |  |  |  |  |  | Ptprg | 0.72 |
|  |  |  |  |  |  | Sri | 0.72 |
|  |  |  |  |  |  | Ablim3 | 0.72 |
|  |  |  |  |  |  | Ext1 | 0.72 |
|  |  |  |  |  |  | Trim25 | 0.72 |
|  |  |  |  |  |  | Plekha5 | 0.72 |
|  |  |  |  |  |  | Rock1 | 0.72 |
|  |  |  |  |  |  | Ky | 0.72 |
|  |  |  |  |  |  | Sema6c | 0.72 |
|  |  |  |  |  |  | Foxp1 | 0.73 |
|  |  |  |  |  |  | Filip1l | 0.73 |
|  |  |  |  |  |  | Rabep2 | 0.73 |
|  |  |  |  |  |  | Mmp14 | 0.73 |
|  |  |  |  |  |  | Ptma | 0.73 |
|  |  |  |  |  |  | Itga1 | 0.73 |
|  |  |  |  |  |  | Ahnak2 | 0.73 |
|  |  |  |  |  |  | Dock1 | 0.74 |
|  |  |  |  |  |  | Cnn2 | 0.74 |
|  |  |  |  |  |  | Ctsh | 0.74 |
|  |  |  |  |  |  | Htra3 | 0.74 |
|  |  |  |  |  |  | Lmod2 | 0.74 |
|  |  |  |  |  |  | Ccdc28b | 0.74 |
|  |  |  |  |  |  | Mxra8 | 0.74 |
|  |  |  |  |  |  | F2r | 0.74 |
|  |  |  |  |  |  | Cav2 | 0.74 |
|  |  |  |  |  |  | Dap | 0.75 |
|  |  |  |  |  |  | Slc25a25 | 0.75 |
|  |  |  |  |  |  | Clic1 | 0.75 |
|  |  |  |  |  |  | Rab31 | 0.75 |
|  |  |  |  |  |  | Scarb1 | 0.75 |
|  |  |  |  |  |  | Dhrs4 | 0.75 |
|  |  |  |  |  |  | Ucp2 | 0.75 |
|  |  |  |  |  |  | Fam114a1 | 0.75 |
|  |  |  |  |  |  | Mcam | 0.75 |
|  |  |  |  |  |  | Dpysl2 | 0.75 |
|  |  |  |  |  |  | Rell1 | 0.76 |
|  |  |  |  |  |  | Cav1 | 0.76 |
|  |  |  |  |  |  | Cd59a | 0.76 |
|  |  |  |  |  |  | Dpysl3 | 0.76 |
|  |  |  |  |  |  | Nek1 | 0.76 |
|  |  |  |  |  |  | Gpx8 | 0.76 |
|  |  |  |  |  |  | Naalad2 | 0.76 |
|  |  |  |  |  |  | Irf1 | 0.76 |
|  |  |  |  |  |  | Acaa2 | 0.76 |
|  |  |  |  |  |  | Ndn | 0.76 |
|  |  |  |  |  |  | Baz1a | 0.76 |
|  |  |  |  |  |  | C1qb | 0.76 |
|  |  |  |  |  |  | Gabbr1 | 0.77 |
|  |  |  |  |  |  | Prex1 | 0.77 |
|  |  |  |  |  |  | Septin10 | 0.77 |
|  |  |  |  |  |  | Elovl5 | 0.77 |
|  |  |  |  |  |  | Engase | 0.77 |
|  |  |  |  |  |  | Dip2a | 0.77 |
|  |  |  |  |  |  | Pde4a | 0.77 |
|  |  |  |  |  |  | Tra2a | 0.77 |
|  |  |  |  |  |  | Cyth4 | 0.78 |
|  |  |  |  |  |  | Laptm5 | 0.78 |
|  |  |  |  |  |  | Robo4 | 0.78 |
|  |  |  |  |  |  | Rasgrp2 | 0.78 |
|  |  |  |  |  |  | Gpc3 | 0.78 |
|  |  |  |  |  |  | Papss2 | 0.78 |
|  |  |  |  |  |  | Hacd1 | 0.78 |
|  |  |  |  |  |  | C1qtnf9 | 0.78 |
|  |  |  |  |  |  | Map4k4 | 0.78 |
|  |  |  |  |  |  | Il10rb | 0.79 |
|  |  |  |  |  |  | Lgmn | 0.79 |
|  |  |  |  |  |  | Tspan12 | 0.79 |
|  |  |  |  |  |  | Angptl1 | 0.79 |
|  |  |  |  |  |  | Fam129b | 0.79 |
|  |  |  |  |  |  | Rgs5 | 0.79 |
|  |  |  |  |  |  | Mrc2 | 0.79 |
|  |  |  |  |  |  | Blvrb | 0.79 |
|  |  |  |  |  |  | Cnp | 0.80 |
|  |  |  |  |  |  | Col18a1 | 0.80 |
|  |  |  |  |  |  | Plxdc1 | 0.80 |
|  |  |  |  |  |  | Olfml3 | 0.80 |
|  |  |  |  |  |  | Ggta1 | 0.80 |
|  |  |  |  |  |  | Plod1 | 0.80 |
|  |  |  |  |  |  | Ier2 | 0.80 |
|  |  |  |  |  |  | Fam171a1 | 0.80 |
|  |  |  |  |  |  | Sox17 | 0.80 |
|  |  |  |  |  |  | Cotl1 | 0.80 |
|  |  |  |  |  |  | Fst | 0.80 |
|  |  |  |  |  |  | Fkbp10 | 0.80 |
|  |  |  |  |  |  | Trim2 | 0.80 |
|  |  |  |  |  |  | Wipf1 | 0.81 |
|  |  |  |  |  |  | Khdrbs3 | 0.81 |
|  |  |  |  |  |  | Ptpn3 | 0.81 |
|  |  |  |  |  |  | Irf9 | 0.81 |
|  |  |  |  |  |  | Gbp7 | 0.81 |
|  |  |  |  |  |  | Armcx4 | 0.81 |
|  |  |  |  |  |  | Rflnb | 0.81 |
|  |  |  |  |  |  | Fkbp7 | 0.81 |
|  |  |  |  |  |  | Parp12 | 0.81 |
|  |  |  |  |  |  | Pgghg | 0.82 |
|  |  |  |  |  |  | Klf4 | 0.82 |
|  |  |  |  |  |  | Lgals9 | 0.82 |
|  |  |  |  |  |  | Cdc42ep1 | 0.82 |
|  |  |  |  |  |  | Adgre5 | 0.82 |
|  |  |  |  |  |  | Ctsk | 0.82 |
|  |  |  |  |  |  | Smc4 | 0.82 |
|  |  |  |  |  |  | Ppp1r18 | 0.82 |
|  |  |  |  |  |  | Palm | 0.82 |
|  |  |  |  |  |  | Zfp36l1 | 0.82 |
|  |  |  |  |  |  | C1qc | 0.82 |
|  |  |  |  |  |  | Sntb2 | 0.82 |
|  |  |  |  |  |  | Sh3bgrl | 0.82 |
|  |  |  |  |  |  | Smtn | 0.82 |
|  |  |  |  |  |  | Brd9 | 0.82 |
|  |  |  |  |  |  | Myo1b | 0.83 |
|  |  |  |  |  |  | Tcf4 | 0.83 |
|  |  |  |  |  |  | Fn1 | 0.83 |
|  |  |  |  |  |  | Itgb1bp2 | 0.83 |
|  |  |  |  |  |  | P2ry1 | 0.83 |
|  |  |  |  |  |  | Tjp1 | 0.83 |
|  |  |  |  |  |  | Fam49a | 0.83 |
|  |  |  |  |  |  | Ttyh3 | 0.83 |
|  |  |  |  |  |  | Col6a2 | 0.83 |
|  |  |  |  |  |  | Fcer1g | 0.83 |
|  |  |  |  |  |  | Sytl2 | 0.83 |
|  |  |  |  |  |  | Eva1b | 0.84 |
|  |  |  |  |  |  | Vim | 0.84 |
|  |  |  |  |  |  | Afdn | 0.84 |
|  |  |  |  |  |  | Tent5a | 0.84 |
|  |  |  |  |  |  | Cdc42ep2 | 0.84 |
|  |  |  |  |  |  | Cntnap2 | 0.84 |
|  |  |  |  |  |  | Tcaf1 | 0.84 |
|  |  |  |  |  |  | Tmsb4x | 0.84 |
|  |  |  |  |  |  | Fap | 0.84 |
|  |  |  |  |  |  | Tanc1 | 0.84 |
|  |  |  |  |  |  | Tnfaip8 | 0.84 |
|  |  |  |  |  |  | Pdlim1 | 0.85 |
|  |  |  |  |  |  | Aqp7 | 0.85 |
|  |  |  |  |  |  | Islr | 0.85 |
|  |  |  |  |  |  | Hes6 | 0.85 |
|  |  |  |  |  |  | Cd200 | 0.85 |
|  |  |  |  |  |  | Pcdh18 | 0.85 |
|  |  |  |  |  |  | Esam | 0.85 |
|  |  |  |  |  |  | Mgp | 0.85 |
|  |  |  |  |  |  | C1qa | 0.86 |
|  |  |  |  |  |  | Rgs4 | 0.86 |
|  |  |  |  |  |  | Bst2 | 0.86 |
|  |  |  |  |  |  | Kitl | 0.86 |
|  |  |  |  |  |  | Yes1 | 0.86 |
|  |  |  |  |  |  | Gucy1a1 | 0.86 |
|  |  |  |  |  |  | Tgfb1i1 | 0.86 |
|  |  |  |  |  |  | Serpinh1 | 0.86 |
|  |  |  |  |  |  | Kdelr3 | 0.86 |
|  |  |  |  |  |  | Morc4 | 0.86 |
|  |  |  |  |  |  | Ptprd | 0.86 |
|  |  |  |  |  |  | Stab1 | 0.86 |
|  |  |  |  |  |  | Dock7 | 0.86 |
|  |  |  |  |  |  | Arhgef3 | 0.86 |
|  |  |  |  |  |  | Tmem88 | 0.86 |
|  |  |  |  |  |  | Col5a3 | 0.87 |
|  |  |  |  |  |  | Pitpnc1 | 0.87 |
|  |  |  |  |  |  | Cd44 | 0.87 |
|  |  |  |  |  |  | Gimap6 | 0.87 |
|  |  |  |  |  |  | Sulf1 | 0.87 |
|  |  |  |  |  |  | Irf5 | 0.87 |
|  |  |  |  |  |  | Cavin2 | 0.87 |
|  |  |  |  |  |  | Map3k1 | 0.87 |
|  |  |  |  |  |  | Gpx7 | 0.87 |
|  |  |  |  |  |  | Zfp950 | 0.87 |
|  |  |  |  |  |  | Calcrl | 0.88 |
|  |  |  |  |  |  | Phldb2 | 0.88 |
|  |  |  |  |  |  | Jam3 | 0.88 |
|  |  |  |  |  |  | Il13ra1 | 0.88 |
|  |  |  |  |  |  | Adamts9 | 0.88 |
|  |  |  |  |  |  | Lcp1 | 0.88 |
|  |  |  |  |  |  | Frmd4a | 0.89 |
|  |  |  |  |  |  | Parp14 | 0.89 |
|  |  |  |  |  |  | Gnb4 | 0.89 |
|  |  |  |  |  |  | Spata6 | 0.89 |
|  |  |  |  |  |  | Nid1 | 0.89 |
|  |  |  |  |  |  | Nudt14 | 0.89 |
|  |  |  |  |  |  | Myl3 | 0.89 |
|  |  |  |  |  |  | Adamts10 | 0.89 |
|  |  |  |  |  |  | Gulp1 | 0.89 |
|  |  |  |  |  |  | Pald1 | 0.90 |
|  |  |  |  |  |  | Tceal5 | 0.90 |
|  |  |  |  |  |  | Antxr1 | 0.90 |
|  |  |  |  |  |  | Lyn | 0.90 |
|  |  |  |  |  |  | Agrn | 0.90 |
|  |  |  |  |  |  | Stard9 | 0.90 |
|  |  |  |  |  |  | Sparcl1 | 0.90 |
|  |  |  |  |  |  | Ptx3 | 0.90 |
|  |  |  |  |  |  | Chd3 | 0.91 |
|  |  |  |  |  |  | Kctd17 | 0.91 |
|  |  |  |  |  |  | Kmt5c | 0.91 |
|  |  |  |  |  |  | Fabp3 | 0.91 |
|  |  |  |  |  |  | Trim30a | 0.91 |
|  |  |  |  |  |  | Id2 | 0.91 |
|  |  |  |  |  |  | F13a1 | 0.91 |
|  |  |  |  |  |  | Lyl1 | 0.91 |
|  |  |  |  |  |  | Itpripl2 | 0.91 |
|  |  |  |  |  |  | Grap | 0.92 |
|  |  |  |  |  |  | Mertk | 0.92 |
|  |  |  |  |  |  | Pmp22 | 0.92 |
|  |  |  |  |  |  | Prkg1 | 0.92 |
|  |  |  |  |  |  | Ptbp3 | 0.92 |
|  |  |  |  |  |  | Emid1 | 0.93 |
|  |  |  |  |  |  | Rap2b | 0.93 |
|  |  |  |  |  |  | Stbd1 | 0.93 |
|  |  |  |  |  |  | Eda | 0.93 |
|  |  |  |  |  |  | Cc2d2a | 0.93 |
|  |  |  |  |  |  | Lipa | 0.93 |
|  |  |  |  |  |  | Thy1 | 0.93 |
|  |  |  |  |  |  | Gimap4 | 0.93 |
|  |  |  |  |  |  | Ano1 | 0.94 |
|  |  |  |  |  |  | Cmtm3 | 0.94 |
|  |  |  |  |  |  | Nckap1l | 0.94 |
|  |  |  |  |  |  | Arl15 | 0.94 |
|  |  |  |  |  |  | Fcgr3 | 0.94 |
|  |  |  |  |  |  | Kcp | 0.94 |
|  |  |  |  |  |  | Sh3kbp1 | 0.94 |
|  |  |  |  |  |  | Igf1 | 0.94 |
|  |  |  |  |  |  | Col6a1 | 0.94 |
|  |  |  |  |  |  | Hcls1 | 0.94 |
|  |  |  |  |  |  | Tnk2 | 0.94 |
|  |  |  |  |  |  | Fam43a | 0.95 |
|  |  |  |  |  |  | Pi16 | 0.95 |
|  |  |  |  |  |  | Cd38 | 0.95 |
|  |  |  |  |  |  | Ets1 | 0.95 |
|  |  |  |  |  |  | Dll4 | 0.95 |
|  |  |  |  |  |  | Map4k2 | 0.95 |
|  |  |  |  |  |  | Chrna1 | 0.96 |
|  |  |  |  |  |  | Igfbp3 | 0.96 |
|  |  |  |  |  |  | Tspan6 | 0.96 |
|  |  |  |  |  |  | Iffo1 | 0.96 |
|  |  |  |  |  |  | Egfl7 | 0.96 |
|  |  |  |  |  |  | Mapk7 | 0.96 |
|  |  |  |  |  |  | Plagl2 | 0.96 |
|  |  |  |  |  |  | Adh1 | 0.97 |
|  |  |  |  |  |  | Lrtm1 | 0.97 |
|  |  |  |  |  |  | Sulf2 | 0.97 |
|  |  |  |  |  |  | Capn6 | 0.97 |
|  |  |  |  |  |  | Ppp1r16b | 0.97 |
|  |  |  |  |  |  | Slfn5 | 0.97 |
|  |  |  |  |  |  | Zdhhc20 | 0.97 |
|  |  |  |  |  |  | Armcx1 | 0.97 |
|  |  |  |  |  |  | Pamr1 | 0.97 |
|  |  |  |  |  |  | Cdr2l | 0.97 |
|  |  |  |  |  |  | Ptprs | 0.98 |
|  |  |  |  |  |  | Smtnl1 | 0.98 |
|  |  |  |  |  |  | Plp1 | 0.98 |
|  |  |  |  |  |  | Obsl1 | 0.98 |
|  |  |  |  |  |  | Cilk1 | 0.98 |
|  |  |  |  |  |  | Ccnd2 | 0.98 |
|  |  |  |  |  |  | Dach1 | 0.98 |
|  |  |  |  |  |  | Parp16 | 0.98 |
|  |  |  |  |  |  | Id3 | 0.99 |
|  |  |  |  |  |  | Ccdc80 | 0.99 |
|  |  |  |  |  |  | Rcn1 | 0.99 |
|  |  |  |  |  |  | Tmc6 | 0.99 |
|  |  |  |  |  |  | Sacs | 0.99 |
|  |  |  |  |  |  | Stxbp4 | 1.00 |
|  |  |  |  |  |  | Tmem131l | 1.00 |
|  |  |  |  |  |  | Aoc3 | 1.00 |
|  |  |  |  |  |  | Tril | 1.00 |
|  |  |  |  |  |  | Sgsm2 | 1.01 |
|  |  |  |  |  |  | Bmf | 1.01 |
|  |  |  |  |  |  | Angptl2 | 1.02 |
|  |  |  |  |  |  | Ushbp1 | 1.02 |
|  |  |  |  |  |  | Opcml | 1.02 |
|  |  |  |  |  |  | Kctd12 | 1.02 |
|  |  |  |  |  |  | Cnrip1 | 1.02 |
|  |  |  |  |  |  | Trpc3 | 1.02 |
|  |  |  |  |  |  | Armcx2 | 1.02 |
|  |  |  |  |  |  | Evl | 1.02 |
|  |  |  |  |  |  | Adgrl4 | 1.02 |
|  |  |  |  |  |  | Sesn3 | 1.02 |
|  |  |  |  |  |  | Unc13b | 1.02 |
|  |  |  |  |  |  | Myl6b | 1.03 |
|  |  |  |  |  |  | Samd9l | 1.03 |
|  |  |  |  |  |  | Dbn1 | 1.03 |
|  |  |  |  |  |  | Cobll1 | 1.03 |
|  |  |  |  |  |  | Plekha4 | 1.03 |
|  |  |  |  |  |  | Gask1b | 1.03 |
|  |  |  |  |  |  | Cldn1 | 1.03 |
|  |  |  |  |  |  | Ccdc88a | 1.04 |
|  |  |  |  |  |  | Hopx | 1.04 |
|  |  |  |  |  |  | Impa2 | 1.04 |
|  |  |  |  |  |  | Efs | 1.05 |
|  |  |  |  |  |  | Dipk2a | 1.05 |
|  |  |  |  |  |  | Trim7 | 1.05 |
|  |  |  |  |  |  | Klhl4 | 1.05 |
|  |  |  |  |  |  | Atp1b4 | 1.05 |
|  |  |  |  |  |  | Adgre1 | 1.06 |
|  |  |  |  |  |  | Cmah | 1.06 |
|  |  |  |  |  |  | Sh2d3c | 1.06 |
|  |  |  |  |  |  | Klhl13 | 1.07 |
|  |  |  |  |  |  | Rbm12b2 | 1.07 |
|  |  |  |  |  |  | Apobec3 | 1.07 |
|  |  |  |  |  |  | Crhr2 | 1.07 |
|  |  |  |  |  |  | Rufy2 | 1.07 |
|  |  |  |  |  |  | Neu2 | 1.07 |
|  |  |  |  |  |  | Clip3 | 1.07 |
|  |  |  |  |  |  | Tmem229b | 1.07 |
|  |  |  |  |  |  | Col8a1 | 1.07 |
|  |  |  |  |  |  | Pcsk5 | 1.07 |
|  |  |  |  |  |  | Mrtfa | 1.08 |
|  |  |  |  |  |  | Mybpc1 | 1.08 |
|  |  |  |  |  |  | Ncam1 | 1.08 |
|  |  |  |  |  |  | Olfml2b | 1.08 |
|  |  |  |  |  |  | Thbs3 | 1.08 |
|  |  |  |  |  |  | Zfp760 | 1.08 |
|  |  |  |  |  |  | Emcn | 1.09 |
|  |  |  |  |  |  | Col4a2 | 1.09 |
|  |  |  |  |  |  | Tmsb10 | 1.09 |
|  |  |  |  |  |  | Fgd2 | 1.09 |
|  |  |  |  |  |  | Lrrc17 | 1.09 |
|  |  |  |  |  |  | Mcm6 | 1.09 |
|  |  |  |  |  |  | Myom3 | 1.10 |
|  |  |  |  |  |  | Myl2 | 1.10 |
|  |  |  |  |  |  | 4930402H24Rik | 1.10 |
|  |  |  |  |  |  | Arsi | 1.10 |
|  |  |  |  |  |  | Plk2 | 1.10 |
|  |  |  |  |  |  | Csrp2 | 1.10 |
|  |  |  |  |  |  | Pld2 | 1.10 |
|  |  |  |  |  |  | N4bp3 | 1.11 |
|  |  |  |  |  |  | Fam217b | 1.11 |
|  |  |  |  |  |  | Tcim | 1.11 |
|  |  |  |  |  |  | Cercam | 1.11 |
|  |  |  |  |  |  | Gimap8 | 1.11 |
|  |  |  |  |  |  | Gxylt2 | 1.12 |
|  |  |  |  |  |  | Hpgd | 1.12 |
|  |  |  |  |  |  | Pgm2 | 1.12 |
|  |  |  |  |  |  | Cd93 | 1.12 |
|  |  |  |  |  |  | Myct1 | 1.13 |
|  |  |  |  |  |  | Dusp6 | 1.13 |
|  |  |  |  |  |  | Scarf1 | 1.14 |
|  |  |  |  |  |  | Trappc6a | 1.14 |
|  |  |  |  |  |  | Samd5 | 1.14 |
|  |  |  |  |  |  | Oasl2 | 1.14 |
|  |  |  |  |  |  | Gem | 1.14 |
|  |  |  |  |  |  | Prox1 | 1.15 |
|  |  |  |  |  |  | Olfr558 | 1.15 |
|  |  |  |  |  |  | Myh1 | 1.15 |
|  |  |  |  |  |  | Xaf1 | 1.15 |
|  |  |  |  |  |  | Pdgfrl | 1.16 |
|  |  |  |  |  |  | Fscn1 | 1.16 |
|  |  |  |  |  |  | Col15a1 | 1.16 |
|  |  |  |  |  |  | Gpsm1 | 1.16 |
|  |  |  |  |  |  | Fndc5 | 1.16 |
|  |  |  |  |  |  | Ncoa7 | 1.16 |
|  |  |  |  |  |  | Loxl2 | 1.16 |
|  |  |  |  |  |  | Lum | 1.17 |
|  |  |  |  |  |  | Nav2 | 1.17 |
|  |  |  |  |  |  | Sntb1 | 1.17 |
|  |  |  |  |  |  | Adamts2 | 1.18 |
|  |  |  |  |  |  | Ccdc85a | 1.18 |
|  |  |  |  |  |  | C1qtnf2 | 1.18 |
|  |  |  |  |  |  | Casp7 | 1.18 |
|  |  |  |  |  |  | Kctd12b | 1.19 |
|  |  |  |  |  |  | Foxo6 | 1.19 |
|  |  |  |  |  |  | Bgn | 1.19 |
|  |  |  |  |  |  | Rnf144a | 1.19 |
|  |  |  |  |  |  | Carns1 | 1.19 |
|  |  |  |  |  |  | Cul7 | 1.19 |
|  |  |  |  |  |  | Mapk8ip1 | 1.20 |
|  |  |  |  |  |  | Sh3rf1 | 1.20 |
|  |  |  |  |  |  | Col6a3 | 1.20 |
|  |  |  |  |  |  | Fcna | 1.20 |
|  |  |  |  |  |  | Mndal | 1.20 |
|  |  |  |  |  |  | Dok4 | 1.20 |
|  |  |  |  |  |  | Adamts20 | 1.20 |
|  |  |  |  |  |  | Pde5a | 1.21 |
|  |  |  |  |  |  | Myoz2 | 1.21 |
|  |  |  |  |  |  | Grb10 | 1.21 |
|  |  |  |  |  |  | Zfp979 | 1.21 |
|  |  |  |  |  |  | Ppp1r9a | 1.22 |
|  |  |  |  |  |  | Rgs10 | 1.22 |
|  |  |  |  |  |  | Snn | 1.22 |
|  |  |  |  |  |  | Col5a1 | 1.23 |
|  |  |  |  |  |  | Gramd1b | 1.23 |
|  |  |  |  |  |  | Spats2l | 1.23 |
|  |  |  |  |  |  | Vstm4 | 1.24 |
|  |  |  |  |  |  | Usp18 | 1.24 |
|  |  |  |  |  |  | Maged2 | 1.25 |
|  |  |  |  |  |  | Nectin2 | 1.25 |
|  |  |  |  |  |  | Rnf122 | 1.26 |
|  |  |  |  |  |  | B3galt2 | 1.26 |
|  |  |  |  |  |  | Prcp | 1.26 |
|  |  |  |  |  |  | Lurap1l | 1.26 |
|  |  |  |  |  |  | Septin6 | 1.26 |
|  |  |  |  |  |  | Gdf10 | 1.27 |
|  |  |  |  |  |  | Mfap5 | 1.27 |
|  |  |  |  |  |  | S100b | 1.27 |
|  |  |  |  |  |  | Plod2 | 1.27 |
|  |  |  |  |  |  | Edn3 | 1.28 |
|  |  |  |  |  |  | Ddx58 | 1.28 |
|  |  |  |  |  |  | Ifit2 | 1.28 |
|  |  |  |  |  |  | Homer2 | 1.29 |
|  |  |  |  |  |  | Mal | 1.29 |
|  |  |  |  |  |  | Smagp | 1.29 |
|  |  |  |  |  |  | Pou2f2 | 1.30 |
|  |  |  |  |  |  | Casp3 | 1.30 |
|  |  |  |  |  |  | Adamtsl2 | 1.30 |
|  |  |  |  |  |  | Pth1r | 1.30 |
|  |  |  |  |  |  | Aif1l | 1.30 |
|  |  |  |  |  |  | Sfrp4 | 1.31 |
|  |  |  |  |  |  | Marcks | 1.31 |
|  |  |  |  |  |  | Col4a1 | 1.31 |
|  |  |  |  |  |  | St8sia4 | 1.32 |
|  |  |  |  |  |  | Fabp5 | 1.33 |
|  |  |  |  |  |  | Ppic | 1.33 |
|  |  |  |  |  |  | Klf2 | 1.33 |
|  |  |  |  |  |  | Tspan11 | 1.33 |
|  |  |  |  |  |  | Aspn | 1.33 |
|  |  |  |  |  |  | Prkg2 | 1.34 |
|  |  |  |  |  |  | Cxcl16 | 1.34 |
|  |  |  |  |  |  | Cmpk2 | 1.34 |
|  |  |  |  |  |  | Npr3 | 1.34 |
|  |  |  |  |  |  | Gpr34 | 1.34 |
|  |  |  |  |  |  | Col5a2 | 1.34 |
|  |  |  |  |  |  | Mettl21e | 1.35 |
|  |  |  |  |  |  | Tlr3 | 1.35 |
|  |  |  |  |  |  | Snai2 | 1.36 |
|  |  |  |  |  |  | Phex | 1.37 |
|  |  |  |  |  |  | Mdk | 1.37 |
|  |  |  |  |  |  | Casq2 | 1.38 |
|  |  |  |  |  |  | Fstl1 | 1.38 |
|  |  |  |  |  |  | Tmem119 | 1.39 |
|  |  |  |  |  |  | Slc38a6 | 1.39 |
|  |  |  |  |  |  | Sema6a | 1.39 |
|  |  |  |  |  |  | Tnmd | 1.40 |
|  |  |  |  |  |  | Arhgap18 | 1.42 |
|  |  |  |  |  |  | Itgb6 | 1.42 |
|  |  |  |  |  |  | BC028528 | 1.43 |
|  |  |  |  |  |  | Lrrn1 | 1.43 |
|  |  |  |  |  |  | Col14a1 | 1.43 |
|  |  |  |  |  |  | Prkcb | 1.44 |
|  |  |  |  |  |  | Mpz | 1.44 |
|  |  |  |  |  |  | Matn2 | 1.44 |
|  |  |  |  |  |  | Cdk14 | 1.45 |
|  |  |  |  |  |  | Col16a1 | 1.45 |
|  |  |  |  |  |  | Stmn1 | 1.45 |
|  |  |  |  |  |  | Serinc5 | 1.45 |
|  |  |  |  |  |  | Eln | 1.46 |
|  |  |  |  |  |  | Sec14l5 | 1.46 |
|  |  |  |  |  |  | Trp53i11 | 1.46 |
|  |  |  |  |  |  | Lpar4 | 1.46 |
|  |  |  |  |  |  | Slc26a10 | 1.47 |
|  |  |  |  |  |  | Pcdh12 | 1.48 |
|  |  |  |  |  |  | Marcksl1 | 1.48 |
|  |  |  |  |  |  | Tbx18 | 1.49 |
|  |  |  |  |  |  | Il16 | 1.49 |
|  |  |  |  |  |  | Ifi203 | 1.49 |
|  |  |  |  |  |  | Myh2 | 1.49 |
|  |  |  |  |  |  | Hs6st2 | 1.49 |
|  |  |  |  |  |  | Lgals4 | 1.49 |
|  |  |  |  |  |  | Irf7 | 1.51 |
|  |  |  |  |  |  | Arntl2 | 1.52 |
|  |  |  |  |  |  | Dgkh | 1.52 |
|  |  |  |  |  |  | Rtl3 | 1.52 |
|  |  |  |  |  |  | Peg3 | 1.54 |
|  |  |  |  |  |  | Id1 | 1.55 |
|  |  |  |  |  |  | Bambi | 1.55 |
|  |  |  |  |  |  | Myh8 | 1.55 |
|  |  |  |  |  |  | Nrep | 1.58 |
|  |  |  |  |  |  | Igsf10 | 1.58 |
|  |  |  |  |  |  | Pcdh17 | 1.59 |
|  |  |  |  |  |  | Bhlhb9 | 1.59 |
|  |  |  |  |  |  | Cttnbp2 | 1.59 |
|  |  |  |  |  |  | Gamt | 1.59 |
|  |  |  |  |  |  | Pxdn | 1.60 |
|  |  |  |  |  |  | AW551984 | 1.61 |
|  |  |  |  |  |  | Etv4 | 1.62 |
|  |  |  |  |  |  | Mfap2 | 1.62 |
|  |  |  |  |  |  | Matn4 | 1.63 |
|  |  |  |  |  |  | Inha | 1.64 |
|  |  |  |  |  |  | Rab15 | 1.64 |
|  |  |  |  |  |  | Isg15 | 1.64 |
|  |  |  |  |  |  | Col4a5 | 1.64 |
|  |  |  |  |  |  | Krt222 | 1.65 |
|  |  |  |  |  |  | Cd86 | 1.66 |
|  |  |  |  |  |  | Id4 | 1.66 |
|  |  |  |  |  |  | Col11a2 | 1.67 |
|  |  |  |  |  |  | Ccn4 | 1.67 |
|  |  |  |  |  |  | Masp1 | 1.67 |
|  |  |  |  |  |  | Gsap | 1.68 |
|  |  |  |  |  |  | Calcr | 1.70 |
|  |  |  |  |  |  | Serpinb1a | 1.72 |
|  |  |  |  |  |  | Mamstr | 1.73 |
|  |  |  |  |  |  | Rspo3 | 1.74 |
|  |  |  |  |  |  | Cdkn1c | 1.74 |
|  |  |  |  |  |  | Sox8 | 1.75 |
|  |  |  |  |  |  | Slamf9 | 1.77 |
|  |  |  |  |  |  | Itm2a | 1.78 |
|  |  |  |  |  |  | Hmcn1 | 1.78 |
|  |  |  |  |  |  | Ifit3 | 1.79 |
|  |  |  |  |  |  | Mest | 1.80 |
|  |  |  |  |  |  | Aplnr | 1.80 |
|  |  |  |  |  |  | Ccdc141 | 1.82 |
|  |  |  |  |  |  | Tnnt2 | 1.83 |
|  |  |  |  |  |  | Sparc | 1.83 |
|  |  |  |  |  |  | Msc | 1.83 |
|  |  |  |  |  |  | Plcb1 | 1.83 |
|  |  |  |  |  |  | Hsbp1l1 | 1.83 |
|  |  |  |  |  |  | Efcc1 | 1.85 |
|  |  |  |  |  |  | Ifi44 | 1.85 |
|  |  |  |  |  |  | Ptn | 1.86 |
|  |  |  |  |  |  | Prkcg | 1.88 |
|  |  |  |  |  |  | Col1a2 | 1.88 |
|  |  |  |  |  |  | Myh3 | 1.90 |
|  |  |  |  |  |  | Mex3b | 1.91 |
|  |  |  |  |  |  | Pnmal2 | 1.91 |
|  |  |  |  |  |  | Col28a1 | 1.91 |
|  |  |  |  |  |  | Cryzl2 | 1.93 |
|  |  |  |  |  |  | Nrk | 1.93 |
|  |  |  |  |  |  | Lgi1 | 1.94 |
|  |  |  |  |  |  | Rsad2 | 1.95 |
|  |  |  |  |  |  | Aqp4 | 1.95 |
|  |  |  |  |  |  | Traf3ip3 | 1.96 |
|  |  |  |  |  |  | Adamts8 | 1.97 |
|  |  |  |  |  |  | Tceal7 | 1.97 |
|  |  |  |  |  |  | Sox4 | 1.97 |
|  |  |  |  |  |  | E2f1 | 2.00 |
|  |  |  |  |  |  | Bcl6b | 2.01 |
|  |  |  |  |  |  | Cx3cr1 | 2.02 |
|  |  |  |  |  |  | Ifit1 | 2.02 |
|  |  |  |  |  |  | Adamts12 | 2.02 |
|  |  |  |  |  |  | Htr7 | 2.03 |
|  |  |  |  |  |  | Frzb | 2.04 |
|  |  |  |  |  |  | Zim1 | 2.06 |
|  |  |  |  |  |  | Gm4841 | 2.06 |
|  |  |  |  |  |  | Pi15 | 2.07 |
|  |  |  |  |  |  | Xpnpep2 | 2.09 |
|  |  |  |  |  |  | Rtp4 | 2.10 |
|  |  |  |  |  |  | Mboat2 | 2.13 |
|  |  |  |  |  |  | Nt5dc2 | 2.18 |
|  |  |  |  |  |  | Vsig2 | 2.19 |
|  |  |  |  |  |  | Dlk1 | 2.20 |
|  |  |  |  |  |  | Col1a1 | 2.20 |
|  |  |  |  |  |  | Vash2 | 2.22 |
|  |  |  |  |  |  | Adamts7 | 2.23 |
|  |  |  |  |  |  | Apln | 2.28 |
|  |  |  |  |  |  | C1qtnf6 | 2.30 |
|  |  |  |  |  |  | Cd83 | 2.31 |
|  |  |  |  |  |  | Tet1 | 2.32 |
|  |  |  |  |  |  | Zdbf2 | 2.33 |
|  |  |  |  |  |  | Arpp21 | 2.34 |
|  |  |  |  |  |  | Fbn2 | 2.37 |
|  |  |  |  |  |  | Col3a1 | 2.40 |
|  |  |  |  |  |  | C1qtnf3 | 2.41 |
|  |  |  |  |  |  | Postn | 2.41 |
|  |  |  |  |  |  | Rasgef1b | 2.44 |
|  |  |  |  |  |  | Gm4951 | 2.45 |
|  |  |  |  |  |  | Col11a1 | 2.46 |
|  |  |  |  |  |  | Mfap4 | 2.59 |
|  |  |  |  |  |  | Robo2 | 2.59 |
|  |  |  |  |  |  | Sez6l2 | 2.59 |
|  |  |  |  |  |  | Plagl1 | 2.70 |
|  |  |  |  |  |  | Aldh1a7 | 2.75 |
|  |  |  |  |  |  | Megf10 | 2.77 |
|  |  |  |  |  |  | Myog | 2.86 |
|  |  |  |  |  |  | Col26a1 | 2.91 |
|  |  |  |  |  |  | Rgs16 | 2.95 |
|  |  |  |  |  |  | Scube2 | 3.07 |
|  |  |  |  |  |  | Actc1 | 3.44 |
|  |  |  |  |  |  | Kcne1l | 3.69 |
|  |  |  |  |  |  | Fndc3c1 | 4.38 |
|  |  |  |  |  |  | Mymk | 5.45 |

| **B** |  |  |  |  |  |  |  |
| --- | --- | --- | --- | --- | --- | --- | --- |
| **Altered miRNAs** | |  | **P adj ≤ 0.05** | |  |  |  |
| **Age effect (YS vs. AS)** | | **Burn in Adults (AB vs. AS)** | | **Burn in Youngs (YB vs. YS)** | | **Burn with age (YB vs. AB)** | |
| **Gene** | **log2FoldChange** | **Gene** | **log2FoldChange** | **Gene** | **log2FoldChange** | **Gene** | **log2FoldChange** |
| mmu-mir-1930 | -3.22 | mmu-mir-10a | -0.39 | mmu-mir-126a | 0.30 | mmu-mir-135a | -2.42 |
| mmu-mir-29a | -2.19 |  |  |  |  | mmu-mir-29a | -2.05 |
| mmu-mir-29b | -1.78 |  |  |  |  | mmu-mir-29b | -1.97 |
| mmu-mir-29c | -1.71 |  |  |  |  | mmu-mir-3061 | -1.68 |
| mmu-mir-1249 | -1.70 |  |  |  |  | mmu-mir-1968 | -1.58 |
| mmu-mir-3061 | -1.58 |  |  |  |  | mmu-mir-1947 | -1.54 |
| mmu-mir-145b | -1.54 |  |  |  |  | mmu-mir-29c | -1.52 |
| mmu-mir-1943 | -1.53 |  |  |  |  | mmu-mir-193a | -1.48 |
| mmu-mir-499 | -1.36 |  |  |  |  | mmu-mir-3962 | -1.44 |
| mmu-mir-22 | -1.29 |  |  |  |  | mmu-mir-6516 | -1.43 |
| mmu-mir-7068 | -1.24 |  |  |  |  | mmu-mir-1943 | -1.43 |
| mmu-mir-155 | -1.14 |  |  |  |  | mmu-mir-6964 | -1.39 |
| mmu-mir-1843a | -1.11 |  |  |  |  | mmu-mir-615 | -1.24 |
| mmu-mir-185 | -1.10 |  |  |  |  | mmu-mir-365 | -1.23 |
| mmu-mir-664 | -1.05 |  |  |  |  | mmu-mir-1249 | -1.17 |
| mmu-mir-149 | -1.04 |  |  |  |  | mmu-mir-664 | -1.14 |
| mmu-mir-378b | -1.02 |  |  |  |  | mmu-mir-22 | -1.09 |
| mmu-mir-1843b | -1.00 |  |  |  |  | mmu-mir-7068 | -1.08 |
| mmu-mir-30a | -1.00 |  |  |  |  | mmu-mir-1843a | -1.00 |
| mmu-mir-1198 | -0.94 |  |  |  |  | mmu-mir-1843b | -0.97 |
| mmu-mir-34a | -0.93 |  |  |  |  | mmu-mir-101a | -0.97 |
| mmu-mir-27a | -0.92 |  |  |  |  | mmu-mir-149 | -0.96 |
| mmu-mir-30e | -0.91 |  |  |  |  | mmu-mir-196a | -0.94 |
| mmu-mir-101a | -0.87 |  |  |  |  | mmu-mir-30c | -0.88 |
| mmu-mir-186 | -0.83 |  |  |  |  | mmu-mir-26a | -0.86 |
| mmu-mir-504 | -0.82 |  |  |  |  | mmu-mir-504 | -0.82 |
| mmu-mir-145a | -0.82 |  |  |  |  | mmu-mir-100 | -0.71 |
| mmu-mir-133b | -0.82 |  |  |  |  | mmu-mir-185 | -0.70 |
| mmu-mir-26a | -0.82 |  |  |  |  | mmu-mir-331 | -0.70 |
| mmu-mir-196a | -0.81 |  |  |  |  | mmu-mir-30a | -0.70 |
| mmu-mir-1a | -0.81 |  |  |  |  | mmu-mir-99a | -0.69 |
| mmu-mir-21c | -0.80 |  |  |  |  | mmu-mir-21c | -0.69 |
| mmu-mir-615 | -0.79 |  |  |  |  | mmu-mir-1981 | -0.67 |
| mmu-mir-10a | -0.76 |  |  |  |  | mmu-mir-150 | -0.67 |
| mmu-mir-151 | -0.73 |  |  |  |  | mmu-let-7d | -0.63 |
| mmu-mir-30c | -0.72 |  |  |  |  | mmu-mir-146b | -0.60 |
| mmu-mir-340 | -0.70 |  |  |  |  | mmu-mir-21a | -0.60 |
| mmu-mir-378c | -0.70 |  |  |  |  | mmu-mir-3068 | -0.58 |
| mmu-mir-378d | -0.67 |  |  |  |  | mmu-mir-133b | -0.57 |
| mmu-mir-328 | -0.67 |  |  |  |  | mmu-mir-98 | -0.57 |
| mmu-mir-365 | -0.67 |  |  |  |  | mmu-mir-345 | -0.55 |
| mmu-mir-361 | -0.66 |  |  |  |  | mmu-mir-361 | -0.54 |
| mmu-mir-21a | -0.66 |  |  |  |  | mmu-mir-27a | -0.53 |
| mmu-mir-100 | -0.62 |  |  |  |  | mmu-mir-186 | -0.52 |
| mmu-mir-30d | -0.62 |  |  |  |  | mmu-mir-328 | -0.52 |
| mmu-mir-204 | -0.59 |  |  |  |  | mmu-mir-26b | -0.50 |
| mmu-mir-423 | -0.58 |  |  |  |  | mmu-mir-1839 | -0.49 |
| mmu-mir-181a | -0.57 |  |  |  |  | mmu-mir-30e | -0.47 |
| mmu-mir-16 | -0.57 |  |  |  |  | mmu-mir-5099 | -0.46 |
| mmu-mir-133a | -0.56 |  |  |  |  | mmu-mir-1a | -0.43 |
| mmu-mir-150 | -0.56 |  |  |  |  | mmu-mir-181b | -0.43 |
| mmu-mir-345 | -0.54 |  |  |  |  | mmu-mir-101b | -0.42 |
| mmu-mir-872 | -0.53 |  |  |  |  | mmu-mir-340 | -0.42 |
| mmu-mir-101b | -0.52 |  |  |  |  | mmu-mir-125a | -0.42 |
| mmu-mir-339 | -0.49 |  |  |  |  | mmu-mir-133a | -0.41 |
| mmu-mir-146b | -0.49 |  |  |  |  | mmu-mir-378a | -0.40 |
| mmu-mir-92a | -0.48 |  |  |  |  | mmu-mir-872 | -0.38 |
| mmu-let-7d | -0.48 |  |  |  |  | mmu-mir-10b | -0.38 |
| mmu-mir-181b | -0.48 |  |  |  |  | mmu-mir-196b | -0.38 |
| mmu-mir-143 | -0.45 |  |  |  |  | mmu-mir-30d | -0.35 |
| mmu-mir-378a | -0.43 |  |  |  |  | mmu-mir-125b | -0.34 |
| mmu-mir-191 | -0.41 |  |  |  |  | mmu-mir-221 | -0.34 |
| mmu-mir-1839 | -0.39 |  |  |  |  | mmu-mir-16 | -0.34 |
| mmu-mir-221 | -0.38 |  |  |  |  | mmu-mir-191 | -0.34 |
| mmu-mir-27b | -0.33 |  |  |  |  | mmu-mir-148b | -0.32 |
| mmu-mir-26b | -0.32 |  |  |  |  | mmu-mir-181a | -0.32 |
| mmu-let-7g | -0.32 |  |  |  |  | mmu-let-7i | -0.31 |
| mmu-mir-192 | -0.31 |  |  |  |  | mmu-let-7g | -0.29 |
| mmu-mir-103 | -0.30 |  |  |  |  | mmu-let-7b | 0.24 |
| mmu-mir-10b | -0.28 |  |  |  |  | mmu-let-7c | 0.29 |
| mmu-mir-450a | 0.37 |  |  |  |  | mmu-let-7e | 0.32 |
| mmu-let-7e | 0.39 |  |  |  |  | mmu-mir-20a | 0.41 |
| mmu-mir-181d | 0.44 |  |  |  |  | mmu-mir-152 | 0.42 |
| mmu-mir-152 | 0.48 |  |  |  |  | mmu-mir-199b | 0.47 |
| mmu-mir-214 | 0.49 |  |  |  |  | mmu-mir-455 | 0.53 |
| mmu-mir-199b | 0.56 |  |  |  |  | mmu-mir-199a | 0.56 |
| mmu-mir-322 | 0.57 |  |  |  |  | mmu-mir-130a | 0.57 |
| mmu-mir-503 | 0.63 |  |  |  |  | mmu-mir-146a | 0.58 |
| mmu-mir-15b | 0.64 |  |  |  |  | mmu-mir-503 | 0.65 |
| mmu-mir-199a | 0.65 |  |  |  |  | mmu-mir-652 | 0.65 |
| mmu-mir-574 | 0.65 |  |  |  |  | mmu-mir-214 | 0.68 |
| mmu-mir-708 | 0.67 |  |  |  |  | mmu-mir-15b | 0.70 |
| mmu-mir-130a | 0.69 |  |  |  |  | mmu-mir-708 | 0.75 |
| mmu-mir-455 | 0.74 |  |  |  |  | mmu-mir-181d | 0.80 |
| mmu-mir-206 | 0.84 |  |  |  |  | mmu-mir-450a | 0.88 |
| mmu-mir-34c | 0.88 |  |  |  |  | mmu-mir-342 | 0.90 |
| mmu-mir-148a | 1.01 |  |  |  |  | mmu-mir-500 | 0.95 |
| mmu-mir-3535 | 1.05 |  |  |  |  | mmu-mir-34c | 0.96 |
| mmu-mir-532 | 1.07 |  |  |  |  | mmu-mir-501 | 0.99 |
| mmu-mir-542 | 1.18 |  |  |  |  | mmu-mir-653 | 1.03 |
| mmu-mir-412 | 1.20 |  |  |  |  | mmu-mir-322 | 1.03 |
| mmu-mir-653 | 1.21 |  |  |  |  | mmu-mir-206 | 1.07 |
| mmu-mir-6236 | 1.25 |  |  |  |  | mmu-mir-362 | 1.15 |
| mmu-mir-500 | 1.26 |  |  |  |  | mmu-mir-412 | 1.16 |
| mmu-mir-362 | 1.27 |  |  |  |  | mmu-mir-532 | 1.22 |
| mmu-mir-501 | 1.31 |  |  |  |  | mmu-mir-351 | 1.41 |
| mmu-mir-335 | 1.44 |  |  |  |  | mmu-mir-376b | 1.45 |
| mmu-mir-323 | 1.45 |  |  |  |  | mmu-mir-298 | 1.61 |
| mmu-mir-376c | 1.46 |  |  |  |  | mmu-mir-675 | 1.63 |
| mmu-mir-376b | 1.47 |  |  |  |  | mmu-mir-539 | 1.65 |
| mmu-mir-666 | 1.48 |  |  |  |  | mmu-mir-136 | 1.76 |
| mmu-mir-329 | 1.51 |  |  |  |  | mmu-mir-542 | 1.79 |
| mmu-mir-351 | 1.53 |  |  |  |  | mmu-mir-369 | 1.80 |
| mmu-mir-6240 | 1.54 |  |  |  |  | mmu-mir-335 | 1.83 |
| mmu-mir-540 | 1.54 |  |  |  |  | mmu-mir-382 | 1.83 |
| mmu-mir-369 | 1.55 |  |  |  |  | mmu-mir-495 | 1.84 |
| mmu-mir-154 | 1.70 |  |  |  |  | mmu-mir-323 | 1.86 |
| mmu-mir-411 | 1.77 |  |  |  |  | mmu-mir-411 | 1.88 |
| mmu-mir-134 | 1.79 |  |  |  |  | mmu-mir-376c | 1.89 |
| mmu-mir-673 | 1.79 |  |  |  |  | mmu-mir-154 | 1.92 |
| mmu-mir-296 | 1.84 |  |  |  |  | mmu-mir-134 | 1.98 |
| mmu-mir-136 | 1.85 |  |  |  |  | mmu-mir-434 | 1.99 |
| mmu-mir-539 | 1.85 |  |  |  |  | mmu-mir-540 | 1.99 |
| mmu-mir-298 | 1.88 |  |  |  |  | mmu-mir-485 | 2.02 |
| mmu-mir-7225 | 1.88 |  |  |  |  | mmu-mir-409 | 2.02 |
| mmu-mir-543 | 1.90 |  |  |  |  | mmu-mir-337 | 2.05 |
| mmu-mir-496a | 1.92 |  |  |  |  | mmu-mir-667 | 2.06 |
| mmu-mir-1193 | 1.94 |  |  |  |  | mmu-mir-1193 | 2.08 |
| mmu-mir-409 | 1.97 |  |  |  |  | mmu-mir-543 | 2.09 |
| mmu-mir-485 | 2.06 |  |  |  |  | mmu-mir-379 | 2.16 |
| mmu-mir-494 | 2.09 |  |  |  |  | mmu-mir-487b | 2.18 |
| mmu-mir-382 | 2.09 |  |  |  |  | mmu-mir-377 | 2.23 |
| mmu-mir-299b | 2.09 |  |  |  |  | mmu-mir-329 | 2.27 |
| mmu-mir-370 | 2.10 |  |  |  |  | mmu-mir-668 | 2.29 |
| mmu-mir-299a | 2.13 |  |  |  |  | mmu-mir-666 | 2.30 |
| mmu-mir-300 | 2.14 |  |  |  |  | mmu-mir-433 | 2.31 |
| mmu-mir-679 | 2.15 |  |  |  |  | mmu-mir-299a | 2.31 |
| mmu-mir-434 | 2.17 |  |  |  |  | mmu-mir-679 | 2.37 |
| mmu-mir-377 | 2.18 |  |  |  |  | mmu-mir-673 | 2.37 |
| mmu-mir-433 | 2.22 |  |  |  |  | mmu-mir-376a | 2.39 |
| mmu-mir-675 | 2.22 |  |  |  |  | mmu-mir-770 | 2.41 |
| mmu-mir-127 | 2.23 |  |  |  |  | mmu-mir-410 | 2.41 |
| mmu-mir-337 | 2.28 |  |  |  |  | mmu-mir-541 | 2.43 |
| mmu-mir-380 | 2.28 |  |  |  |  | mmu-mir-493 | 2.46 |
| mmu-mir-495 | 2.29 |  |  |  |  | mmu-mir-494 | 2.48 |
| mmu-mir-376a | 2.30 |  |  |  |  | mmu-mir-380 | 2.51 |
| mmu-mir-341 | 2.33 |  |  |  |  | mmu-mir-341 | 2.58 |
| mmu-mir-770 | 2.34 |  |  |  |  | mmu-mir-127 | 2.61 |
| mmu-mir-541 | 2.34 |  |  |  |  | mmu-mir-496a | 2.68 |
| mmu-mir-379 | 2.48 |  |  |  |  | mmu-mir-300 | 2.82 |
| mmu-mir-410 | 2.51 |  |  |  |  | mmu-mir-483 | 2.83 |
| mmu-mir-483 | 2.53 |  |  |  |  | mmu-mir-1197 | 3.19 |
| mmu-mir-3072 | 2.56 |  |  |  |  | mmu-mir-431 | 3.29 |
| mmu-mir-493 | 2.73 |  |  |  |  | mmu-mir-370 | 3.42 |
| mmu-mir-1197 | 2.91 |  |  |  |  | mmu-mir-381 | 4.09 |
| mmu-mir-667 | 3.01 |  |  |  |  | mmu-mir-544 | 4.44 |
| mmu-mir-487b | 3.09 |  |  |  |  | mmu-mir-665 | 5.73 |
| mmu-mir-431 | 3.13 |  |  |  |  |  |  |
| mmu-mir-668 | 3.14 |  |  |  |  |  |  |
| mmu-mir-381 | 3.24 |  |  |  |  |  |  |
| mmu-mir-665 | 3.30 |  |  |  |  |  |  |
| mmu-mir-758 | 4.22 |  |  |  |  |  |  |
| mmu-mir-3099 | 4.38 |  |  |  |  |  |  |
| mmu-mir-546 | 5.07 |  |  |  |  |  |  |
