# Supplementary material for: Skeletal muscle transcriptome is affected by age in severely burned mice
Source: Sci Rep. 2022 Dec 14;12:21584. doi: 10.1038/s41598-022-26040-1 (PMC9748408; doi:10.1038/s41598-022-26040-1)
Supplement: Supplementary file 2 — Supplementary Information 2. [file 41598_2022_26040_MOESM2_ESM.docx]

| **A** |  |  |  |  |
| --- | --- | --- | --- | --- |
| Hallmark gene sets are coherently expressed signatures derived by aggregating many MSigDB gene sets to represent well-defined biological states or processes. | | | | |
| **Collections** | **# Overlaps Shown** | **# Gene Sets in Collections** | **# Genes in Comparison (n)** | **# Genes in Universe (N)** |
| **YS v.AS** | 38 | 50 | 1,106 | 40,071 |
| **AB v. AS** | 21 | 50 | 204 | 40,071 |
| **YB v. YS** | 20 | 50 | 179 | 40,071 |
| **YB v. AB** | 46 | 50 | 1,816 | 40,071 |
|  |  |  |  |  |
|  |  |  |  |  |
| **B** |  |  |  |  |
| Canonical Pathways gene sets derived from the WikiPathways database. | | | | |
| **Collections** | **# Overlaps Shown** | **# Gene Sets in Collections** | **# Genes in Comparison (n)** | **# Genes in Universe (N)** |
| **YS v.AS** | 100 | 587 | 1,106 | 40,071 |
| **AB v. AS** | 24 | 587 | 204 | 40,071 |
| **YB v. YS** | 15 | 587 | 179 | 40,071 |
| **YB v. AB** | 100 | 587 | 1,816 | 40,071 |
|  |  |  |  |  |
|  |  |  |  |  |
| **C** |  |  |  |  |
| Altered genes predicted to be targets of listed microRNA hsa-miR-6867-5p in miRDB v6.0 with MirTarget v4 prediction scores > 80 (high confidence targets). | | | | |
| **miRNAs** | **# Overlaps Shown** | **# Gene Sets in Collections** | **# Genes in Comparison (n)** | **# Genes in Universe (N)** |
| **YS v.AS** | 100 | 2,598 | 1,106 | 40,071 |
| **AB v. AS** | 30 | 2,598 | 204 | 40,071 |
| **YB v. YS** | 14 | 2,598 | 179 | 40,071 |
| **YB v. AB** | 100 | 2,598 | 1,816 | 40,071 |
