# Supplementary material for: Skeletal muscle transcriptome is affected by age in severely burned mice
Source: Sci Rep. 2022 Dec 14;12:21584. doi: 10.1038/s41598-022-26040-1 (PMC9748408; doi:10.1038/s41598-022-26040-1)
Supplement: Supplementary file 3 — Supplementary Information 3. [file 41598_2022_26040_MOESM3_ESM.docx]

| **A** | **Biological processing (BP) with Up- or Down-regulated genes**  **Supplement Table 3** 50 top up-regulated or down-regulated DEGs overlapping with **(A)** **Biological processing (BP)** and (**B**) **Wiki-pathways** gene sets. Top 10 gene sets listed following the order of significance of p-value and FDR q-value. The comparison groups include young sham (YS), young burn (YB), adult sham (AS), and adult burn (AB) groups. | | |  |  |  |
| --- | --- | --- | --- | --- | --- | --- |
|  |  | **Age effect (YS vs. AS)** | **Burn in Adults (AB vs. AS)** | **Burn in Youngs (YB vs. YS)** | **Burn with age (YB vs. AB)** |  |
|  | **with Up-regulated genes** | |  |  |  |  |
|  | #01 | .SKELETAL_SYSTEM_DEVELOPMENT [570] | .APOPTOTIC_PROCESS [1974] | .DEFENSE_RESPONSE [1816] | .REGULATION_OF_CELL_DIFFERENTIATION [1732] | |
|  | #02 | .EXTERNAL_ENCAPSULATING_STRUCTURE_ORGANIZATION [325] | .REGULATION_OF_CELL_DEATH [1796] | .INFLAMMATORY_RESPONSE [770] | .SKELETAL_SYSTEM_DEVELOPMENT [570] | |
|  | #03 | .REGULATION_OF_CELL_DIFFERENTIATION [1732] | .BLOOD_VESSEL_MORPHOGENESIS [662] | .BIOLOGICAL_PROCESS_INVOLVED_IN_INTERSPECIES_INTERACTION_BETWEEN_ORGANISMS [1835] | .POSITIVE_REGULATION_OF_MULTICELLULAR_ORGANISMAL_PROCESS [1722] | |
|  | #04 | .COLLAGEN_FIBRIL_ORGANIZATION [60] | .TUBE_MORPHOGENESIS [960] | .IMMUNE_RESPONSE [1920] | .CARTILAGE_DEVELOPMENT [216] | |
|  | #05 | .CELL_ADHESION [1480] | .REGULATION_OF_IMMUNE_SYSTEM_PROCESS [1642] | .DEFENSE_RESPONSE_TO_OTHER_ORGANISM [1236] | .CONNECTIVE_TISSUE_DEVELOPMENT [302] | |
|  | #06 | .ANATOMICAL_STRUCTURE_FORMATION_INVOLVED_IN_MORPHOGENESIS [1238] | .REGULATION_OF_PLATELET_DERIVED_GROWTH_FACTOR_RECEPTOR_SIGNALING_PATHWAY [25] | .GRANULOCYTE_CHEMOTAXIS [129] | .EXTERNAL_ENCAPSULATING_STRUCTURE_ORGANIZATION [325] | |
|  | #07 | .CARTILAGE_DEVELOPMENT [216] | .ANGIOGENESIS [549] | .LEUKOCYTE_MIGRATION [384] | .CHONDROCYTE_DIFFERENTIATION [121] | |
|  | #08 | .CHONDROCYTE_DIFFERENTIATION [121] | .VASCULATURE_DEVELOPMENT [797] | .REGULATION_OF_IMMUNE_SYSTEM_PROCESS [1642] | .COLLAGEN_FIBRIL_ORGANIZATION [60] | |
|  | #09 | .EMBRYO_DEVELOPMENT [1343] | .RESPONSE_TO_DEXAMETHASONE [29] | .GRANULOCYTE_MIGRATION [162] | .ANATOMICAL_STRUCTURE_FORMATION_INVOLVED_IN_MORPHOGENESIS [1238] | |
|  | #10 | .DEVELOPMENTAL_GROWTH [818] | .TUBE_DEVELOPMENT [1204] | .POSITIVE_REGULATION_OF_IMMUNE_SYSTEM_PROCESS [1086] | .POSITIVE_REGULATION_OF_DEVELOPMENTAL_PROCESS [1531] | |
|  | **with Down-regulated genes** | |  |  |  |  |
|  | #01 | .POSITIVE_REGULATION_OF_CELL_DEATH [690] | .MUSCLE_SYSTEM_PROCESS [432] | .REFLEX [27] | .REGULATION_OF_CELL_DEATH [1796] | |
|  | #02 | .REGULATION_OF_CELL_DEATH [1796] | .CIRCULATORY_SYSTEM_PROCESS [570] | .APOPTOTIC_PROCESS [1974] | .POLYAMINE_BIOSYNTHETIC_PROCESS [15] | |
|  | #03 | .BONE_DEVELOPMENT [259] | .MUSCLE_CONTRACTION [317] | .PROTEIN_ACYLATION [277] | .POSITIVE_REGULATION_OF_CELL_DEATH [690] | |
|  | #04 |  | .REGULATION_OF_BLOOD_CIRCULATION [270] | | .APOPTOTIC_PROCESS [1974] |  |
|  | #05 |  | .CHEMICAL_HOMEOSTASIS [1121] | | .POLYAMINE_METABOLIC_PROCESS [23] | |
|  | #06 |  | .REGULATION_OF_SYSTEM_PROCESS [661] | | .POSITIVE_REGULATION_OF_SIGNAL_TRANSDUCTION [1582] | |
|  | #07 |  | .RESPONSE_TO_ACTIVITY [38] | | .SPERMIDINE_BIOSYNTHETIC_PROCESS [5] | |
|  | #08 |  | .REGULATION_OF_HEART_CONTRACTION [201] | | .AMINE_BIOSYNTHETIC_PROCESS [43] | |
|  | #09 |  | .MUSCLE_STRUCTURE_DEVELOPMENT [740] | | .RESPONSE_TO_INACTIVITY [6] | |
|  | #10 |  | .REGULATION_OF_TRANSPORT [1946] | | .SPERMINE_METABOLIC_PROCESS [7] | |
|  |  |  |  |  |  |  |
|  |  | | |  |  |  |
| **B** | **WiKi-pathways with Up- or Down regulated genes** | | |  |  |  |
|  |  | **Age effect (YS vs. AS)** | **Burn in Adults (AB vs. AS)** | **Burn in Youngs (YB vs. YS)** | **Burn with age (YB vs. AB)** |  |
|  | **with Up-regulated genes** | |  |  |  |  |
|  | #01 | .FOCAL_ADHESION [185] | .PROTEASOME_DEGRADATION [51] | .PROTEASOME_DEGRADATION [51] | .FOCAL_ADHESION [185] |  |
|  | #02 | .FOCAL_ADHESION_PI3KAKTMTOR_SIGNALING_PATHWAY [317] | .OXIDATIVE_STRESS_RESPONSE [28] | .MICROGLIA_PATHOGEN_PHAGOCYTOSIS_PATPATHWAY [41] | .GPCRS_CLASS_B_SECRETINLIKE [22] | |
|  | #03 | .ELECTRON_TRANSPORT_CHAIN [92] | .OXIDATIVE_STRESS_AND_REDOX_PATHWAY [92] | .COMPLEMENT_ACTIVATION_CLASSICAL_PATPATHWAY [17] | .FOCAL_ADHESION_PI3KAKTMTOR_SIGNALING_PATHWAY [317] | |
|  | #04 | .ADIPOGENESIS_GENES [132] | .FOLIC_ACID_NETWORK [22] | .TYROBP_CAUSAL_NETWORK_IN_MICROGLIA [58] | .DYSREGULATED_MIRNA_TARGETING_IN_INSULINPI3KAKT_SIGNALING [27] | |
|  | #05 | .MYOMETRIAL_RELAXATION_AND_CONTRACTION_PATHWAYS [149] | .ONECARBON_METABOLISM_AND_RELATED_PATHWAYS [52] | .OXIDATIVE_DAMAGE_RESPONSE [40] | .INFLAMMATORY_RESPONSE_PATHWAY [30] | |
|  | #06 | .MRNA_PROCESSING [449] |  | .MACROPHAGE_MARKERS [10] | .MYOMETRIAL_RELAXATION_AND_CONTRACTION_PATHWAYS [149] | |
|  | #07 | .DYSREGULATED_MIRNA_TARGETING_IN_INSULINPI3KAKT_SIGNALING [27] | | | .ID_SIGNALING_PATHWAY [50] | |
|  | #08 | .INFLAMMATORY_RESPONSE_PATHWAY [30] | |  | .TYROBP_CAUSAL_NETWORK_IN_MICROGLIA [58] | |
|  | #09 | .REGULATION_OF_ACTIN_CYTOSKELETON [150] | |  | .OXIDATIVE_DAMAGE_RESPONSE [40] | |
|  | #10 | .G13_SIGNALING_PATHWAY [38] |  |  | .KIT_RECEPTOR_SIGNALING_PATHWAY [67] | |
|  | **with Down-regulated genes** | |  |  |  |  |
|  | #01 | .INSULIN_SIGNALING [158] | .MYOMETRIAL_RELAXATION_AND_CONTRACTICTION_PATHWAYS [149] | .FOCAL_ADHESION_PI3KAKTMTOR_SIGNALINLING_PATHWAY [317] | .EXERCISEINDUCED_CIRCADIAN_REGULATIOTION [49] | |
|  | #02 | .HYPOXIADEPENDENT_DIFFERENTIATION_OF_MYOBLASTS [13] | .FATTY_ACID_OXIDATION [10] | | .ADIPOGENESIS_GENES [132] |  |
|  | #03 | .AMINO_ACID_METABOLISM [95] | .FATTY_ACID_BETAOXIDATION [34] | | .IL3_SIGNALING_PATHWAY [99] | |
|  | #04 | .OXIDATIVE_STRESS_RESPONSE [28] | .ID_SIGNALING_PATHWAY [50] | | .ONECARBON_METABOLISM [29] | |
|  | #05 | .CHOLESTEROL_METABOLISM_WITH_BLOCH_AH_AND_KANDUTSCHRUSSELL_PATHWAYS [52] | .CALCIUM_REGULATION_IN_CARDIAC_CELLS [144] | | .CHOLESTEROL_METABOLISM_WITH_BLOCH_AH_AND_KANDUTSCHRUSSELL_PATHWAYS [52] | |
|  | #06 | .FOCAL_ADHESION_PI3KAKTMTOR_SIGNALINLING_PATHWAY [317] | .EGFR1_SIGNALING_PATHWAY [175] | | .MYOMETRIAL_RELAXATION_AND_CONTRACTICTION_PATHWAYS [149] | |
|  | #07 | .ADIPOGENESIS_GENES [132] | .MITOCHONDRIAL_LONG_CHAIN_FATTY_ACID_BETAOXIDATION [16] | | .G1_TO_S_CELL_CYCLE_CONTROL [60] | |
|  | #08 | .GLYCOLYSIS_AND_GLUCONEOGENESIS [49] | .PPAR_SIGNALING_PATHWAY [81] | | .NUCLEAR_RECEPTORS [38] |  |
|  | #09 | .CALCIUM_REGULATION_IN_CARDIAC_CELLS [144] | .G_PROTEIN_SIGNALING_PATHWAYS [89] | | .FOCAL_ADHESION_PI3KAKTMTOR_SIGNALINLING_PATHWAY [317] | |
|  | #10 | .OMEGA3_OMEGA6_FATTY_ACID_SYNTHESIS [15] | .FATTY_ACID_BIOSYNTHESIS [22] | | .OXIDATIVE_STRESS_RESPONSE [28] | |
