# Supplementary material for: Skeletal muscle transcriptome is affected by age in severely burned mice
Source: Sci Rep. 2022 Dec 14;12:21584. doi: 10.1038/s41598-022-26040-1 (PMC9748408; doi:10.1038/s41598-022-26040-1)
Supplement: Supplementary file 4 — Supplementary Information 4. [file 41598_2022_26040_MOESM4_ESM.pdf]

Supplemental file 4: RNA sample quality report. 12 Total RNA samples were assessed by automated gel electrophoresis using Agilent 2100 bioanalyzer (Agilent Tech. Inc.). Each RNA sample was labeled with acronym: SV3-experiment acronym; young sham (YS), young burn (YB), adult sham (AS), and adult burn (AB) groups.

Assay Class: Eukaryote Total RNA Nano
Data Path: C:\...Eukaryote Total RNA Nano_DE13804763_2020-08-12_11-44-08.xad

Created: 8/12/2020 11:44:08 AM
Modified: 8/12/2020 12:24:19 PM

## Electrophoresis File Run Summary

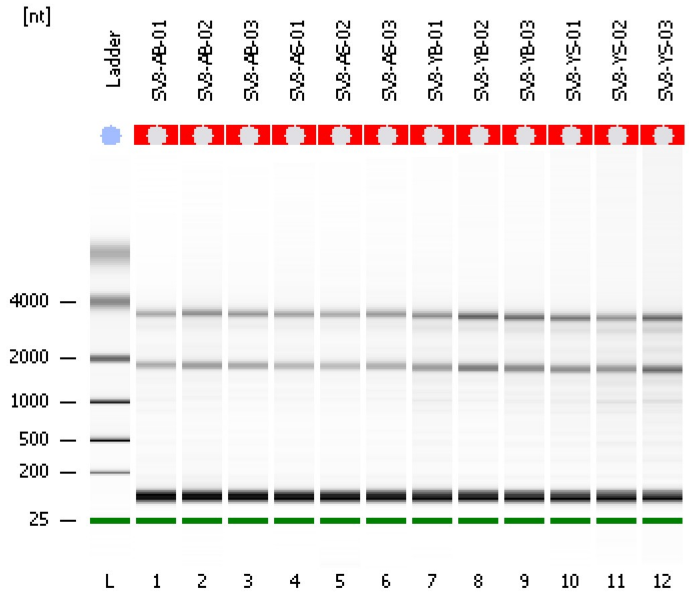


Instrument Information:

Instrument Name: DE13804763
Firmware: C.01.069
Serial#: DE13804763
Type: G2939A

Assay Information:

Assay Origin Path: C:\Program Files\Agilent\2100 bioanalyzer\2100 expert\assays\RNA\Eukaryote Total RNA Nano Series II.xsy

Assay Class: Eukaryote Total RNA Nano

Version: 2.6

Assay Comments: Total RNA Analysis ng sensitivity (Eukaryote)

© Copyright 2003 - 2009 Agilent Technologies, Inc.

Chip Information:

Chip Lot #:

Reagent Kit Lot #:

Chip Comments:

**SV3-AB-01**

RIN N/A

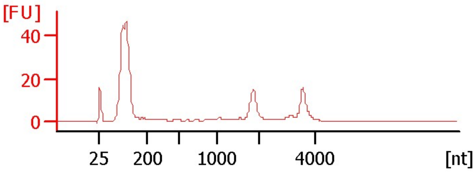


**SV3-AB-02**

RIN N/A

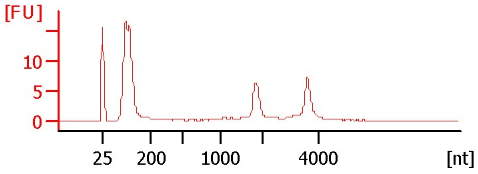


**SV3-AB-03**

RIN N/A

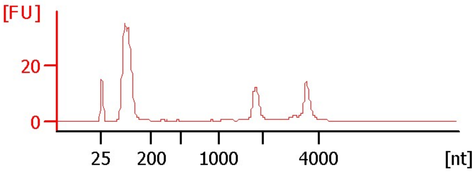


**SV3-AS-01**

RIN N/A

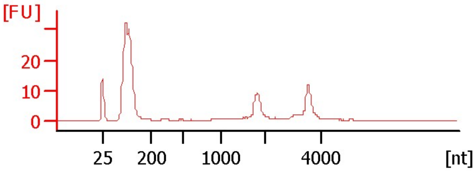


**SV3-AS-02**

RIN N/A

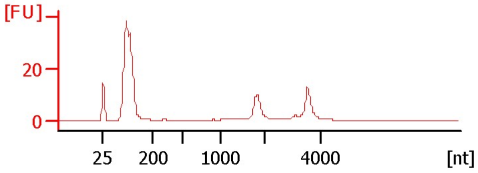


**SV3-AS-03**

RIN N/A

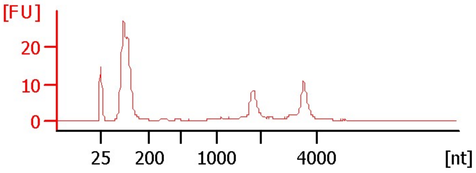


**SV3-YB-01**

RIN N/A

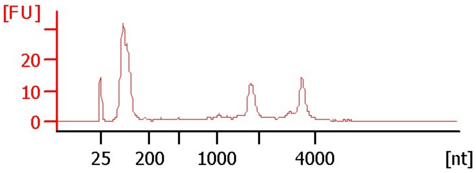


**SV3-YB-02**

RIN N/A

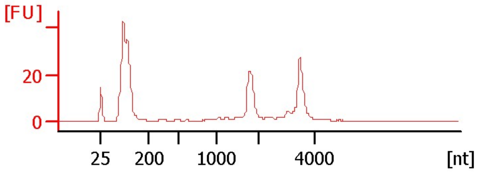


**SV3-YB-03**

RIN N/A

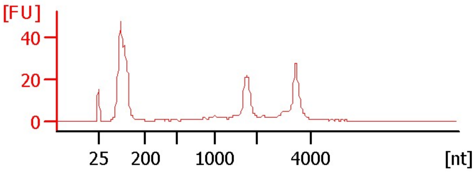


**SV3-YS-01**

RIN N/A

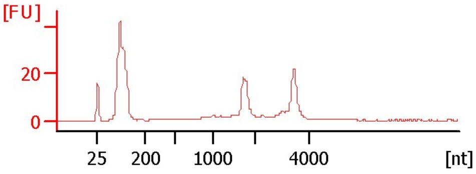


**SV3-YS-02**

RIN N/A

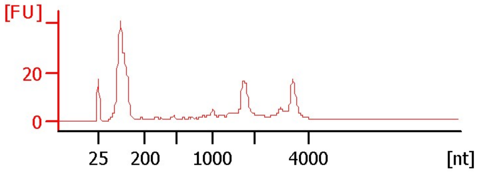


**SV3-YS-03**

RIN N/A

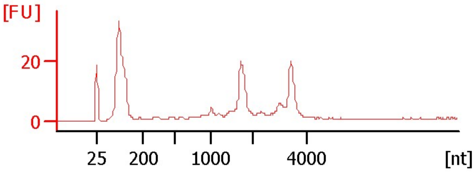



© Copyright 2003 - 2009 Agilent Technologies, Inc.

Assay Class: Eukaryote Total RNA Nano
Data Path: C:\...Eukaryote Total RNA Nano_DE13804763_2020-08-12_11-44-08.xad
Created: 8/12/2020 11:44:08 AM
Modified: 8/12/2020 12:24:19 PM

## Electrophoresis File Run Summary (Chip Summary)

| Sample Name | Sample Comment | Status | Result Label | Result Color |
|---|---|---|---|---|
| SV3-AB-01 | | ✓ | RIN N/A | |
| SV3-AB-02 | | ✓ | RIN N/A | |
| SV3-AB-03 | | ✓ | RIN N/A | |
| SV3-AS-01 | | ✓ | RIN N/A | |
| SV3-AS-02 | | ✓ | RIN N/A | |
| SV3-AS-03 | | ✓ | RIN N/A | |
| SV3-YB-01 | | ✓ | RIN N/A | |
| SV3-YB-02 | | ✓ | RIN N/A | |
| SV3-YB-03 | | ✓ | RIN N/A | |
| SV3-YS-01 | | ✓ | RIN N/A | |
| SV3-YS-02 | | ✓ | RIN N/A | |
| SV3-YS-03 | | ✓ | RIN N/A | |
| Ladder | | ✓ | All Other Samples | |

**Chip Lot #** **Reagent Kit Lot #**

**Chip Comments :**


© Copyright 2003 - 2009 Agilent Technologies, Inc.

Assay Class: Eukaryote Total RNA Nano
Data Path: C:\...Eukaryote Total RNA Nano_DE13804763_2020-08-12_11-44-08.xad
Created: 8/12/2020 11:44:08 AM
Modified: 8/12/2020 12:24:19 PM

## Electrophoresis Assay Details

### General Analysis Settings

Number of Available Sample and Ladder Wells (Max.) : 13
Minimum Visible Range [s] : 17
Maximum Visible Range [s] : 70
Start Analysis Time Range [s] : 19
End Analysis Time Range [s] : 69
Ladder Concentration [ng/µl] : 150
Lower Marker Concentration [ng/µl] : 0
Upper Marker Concentration [ng/µl] : 0
Used Lower Marker for Quantitation
Standard Curve Fit is Logarithmic
Show Data Aligned to Lower Marker

### Integrator Settings

Integration Start Time [s] : 19
Integration End Time [s] : 69
Slope Threshold : 0.6
Height Threshold [FU] : 0.5
Area Threshold : 0.2
Width Threshold [s] : 0.5
Baseline Plateau [s] : 6

### Filter Settings

Filter Width [s] : 0.5
Polynomial Order : 4

### Ladder

| Ladder Peak | Size |
|---|---|
| 1 | 25 |
| 2 | 200 |
| 3 | 500 |
| 4 | 1000 |
| 5 | 2000 |
| 6 | 4000 |

 © Copyright 2003 - 2009 Agilent Technologies, Inc. 

Assay Class: Eukaryote Total RNA Nano
Data Path: C:\...Eukaryote Total RNA Nano_DE13804763_2020-08-12_11-44-08.xad

Created: 8/12/2020 11:44:08 AM
Modified: 8/12/2020 12:24:19 PM

**Electropherogram Summary**

SV3-AB-01

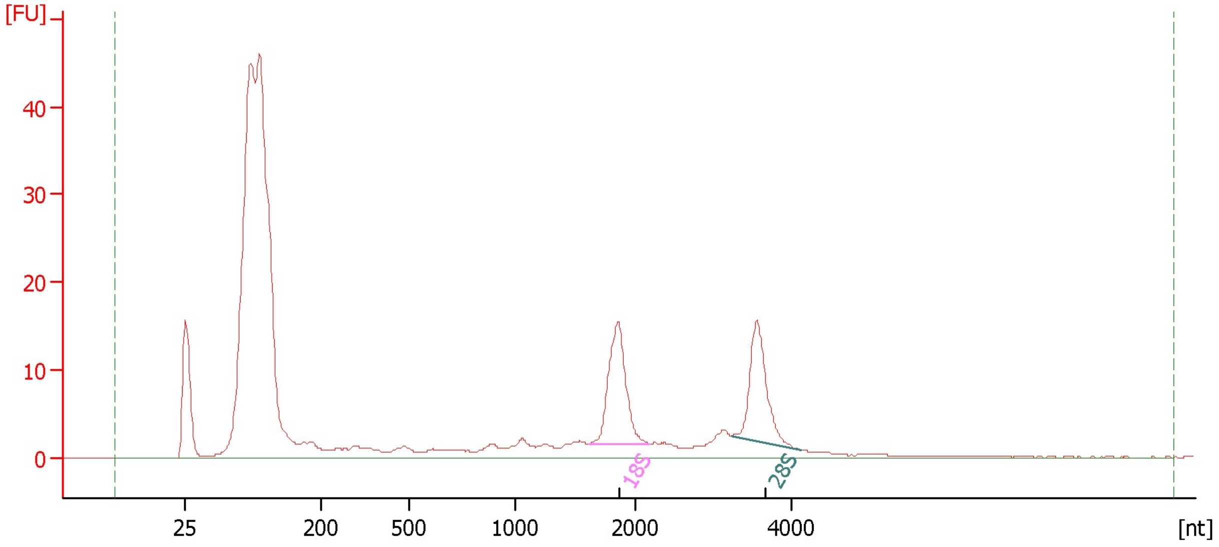


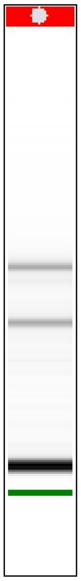

**Overall Results for sample 1 : SV3-AB-01**

| | | | |
|---|---|---|---|
| RNA Area: | 386.0 | RNA Integrity Number (RIN): | N/A (B.02.08) |
| RNA Concentration: | 339 ng/µl | Result Flagging Color: | |
| rRNA Ratio [28s / 18s]: | 0.8 | Result Flagging Label: | RIN N/A |

**Fragment table for sample 1 : SV3-AB-01**

| Name | Start Size [nt] | End Size [nt] | Area | % of total Area |
|---|---|---|---|---|
| 18S | 1,617 | 2,192 | 29.8 | 7.7 |
| 28S | 3,231 | 4,133 | 24.9 | 6.5 |


© Copyright 2003 - 2009 Agilent Technologies, Inc.

Assay Class: Eukaryote Total RNA Nano
Data Path: C:\...Eukaryote Total RNA Nano_DE13804763_2020-08-12_11-44-08.xad

Created: 8/12/2020 11:44:08 AM
Modified: 8/12/2020 12:24:19 PM

**Electropherogram Summary Continued ...**

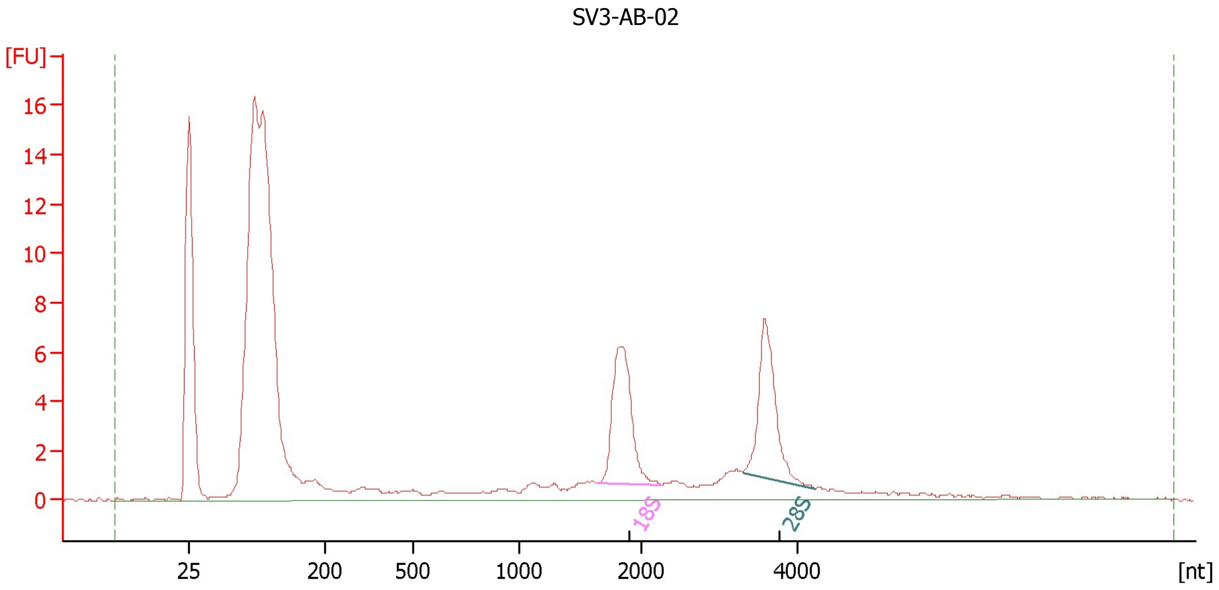


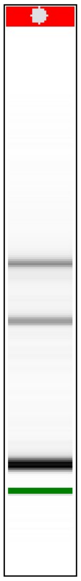

**Overall Results for sample 2 : SV3-AB-02**

| | | | |
|---|---|---|---|
| RNA Area: | 150.3 | RNA Integrity Number (RIN): | N/A (B.02.08) |
| RNA Concentration: | 132 ng/µl | Result Flagging Color: | |
| rRNA Ratio [28s / 18s]: | 0.9 | Result Flagging Label: | RIN N/A |

**Fragment table for sample 2 : SV3-AB-02**

| Name | Start Size [nt] | End Size [nt] | Area | % of total Area |
|---|---|---|---|---|
| 18S | 1,639 | 2,248 | 13.4 | 8.9 |
| 28S | 3,294 | 4,217 | 11.5 | 7.7 |


© Copyright 2003 - 2009 Agilent Technologies, Inc.

Assay Class: Eukaryote Total RNA Nano
Data Path: C:\...Eukaryote Total RNA Nano_DE13804763_2020-08-12_11-44-08.xad

Created: 8/12/2020 11:44:08 AM
Modified: 8/12/2020 12:24:19 PM

**Electropherogram Summary Continued ...**

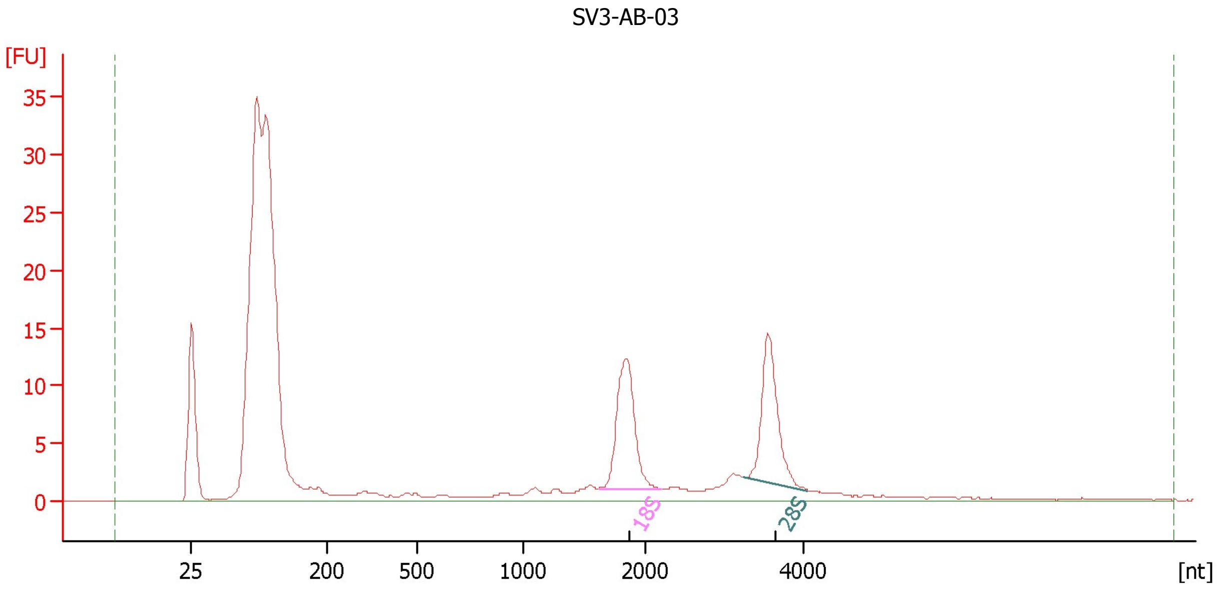


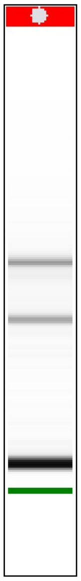

**Overall Results for sample 3 : SV3-AB-03**

| | | | |
|---|---|---|---|
| RNA Area: | 281.4 | RNA Integrity Number (RIN): | N/A (B.02.08) |
| RNA Concentration: | 247 ng/µl | Result Flagging Color: | |
| rRNA Ratio [28s / 18s]: | 0.9 | Result Flagging Label: | RIN N/A |

**Fragment table for sample 3 : SV3-AB-03**

| Name | Start Size [nt] | End Size [nt] | Area | % of total Area |
|---|---|---|---|---|
| 18S | 1,616 | 2,209 | 25.0 | 8.9 |
| 28S | 3,250 | 4,061 | 21.9 | 7.8 |

 © Copyright 2003 - 2009 Agilent Technologies, Inc. 

Assay Class: Eukaryote Total RNA Nano
Data Path: C:\...Eukaryote Total RNA Nano_DE13804763_2020-08-12_11-44-08.xad

Created: 8/12/2020 11:44:08 AM
Modified: 8/12/2020 12:24:19 PM

**Electropherogram Summary Continued ...**

SV3-AS-01

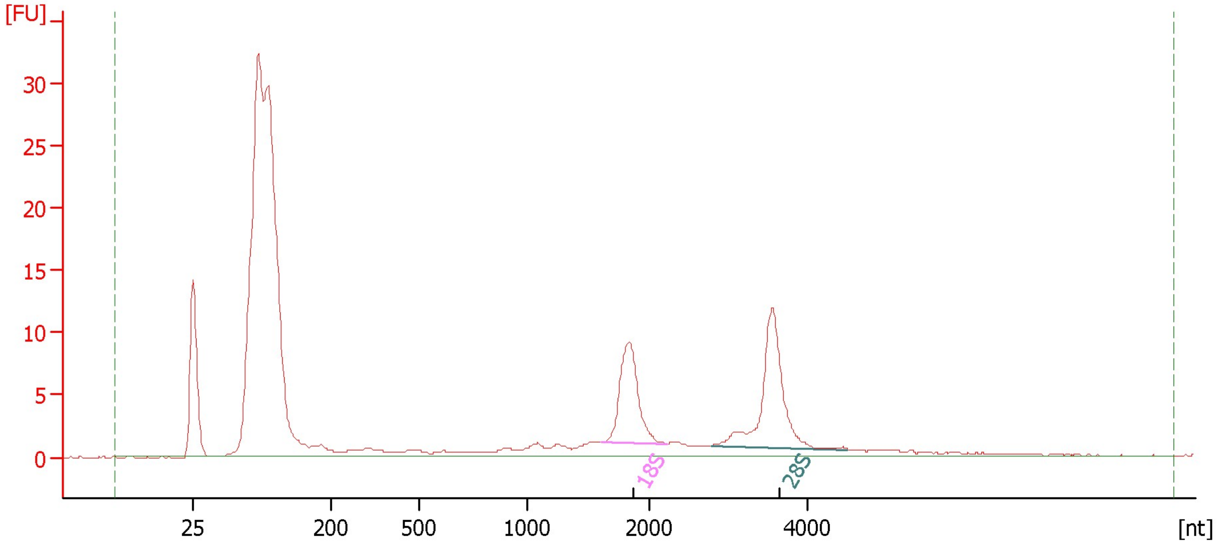


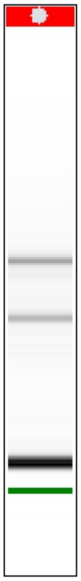

**Overall Results for sample 4 : SV3-AS-01**

| | | | |
|---|---|---|---|
| RNA Area: | 254.1 | RNA Integrity Number (RIN): | N/A (B.02.08) |
| RNA Concentration: | 223 ng/µl | Result Flagging Color: | |
| rRNA Ratio [28s / 18s]: | 1.3 | Result Flagging Label: | RIN N/A |

**Fragment table for sample 4 : SV3-AS-01**

| Name | Start Size [nt] | End Size [nt] | Area | % of total Area |
|---|---|---|---|---|
| 18S | 1,610 | 2,223 | 18.6 | 7.3 |
| 28S | 2,789 | 4,512 | 24.5 | 9.6 |

 © Copyright 2003 - 2009 Agilent Technologies, Inc. 

Assay Class: Eukaryote Total RNA Nano
Data Path: C:\...Eukaryote Total RNA Nano_DE13804763_2020-08-12_11-44-08.xad

Created: 8/12/2020 11:44:08 AM
Modified: 8/12/2020 12:24:19 PM

**Electropherogram Summary Continued ...**

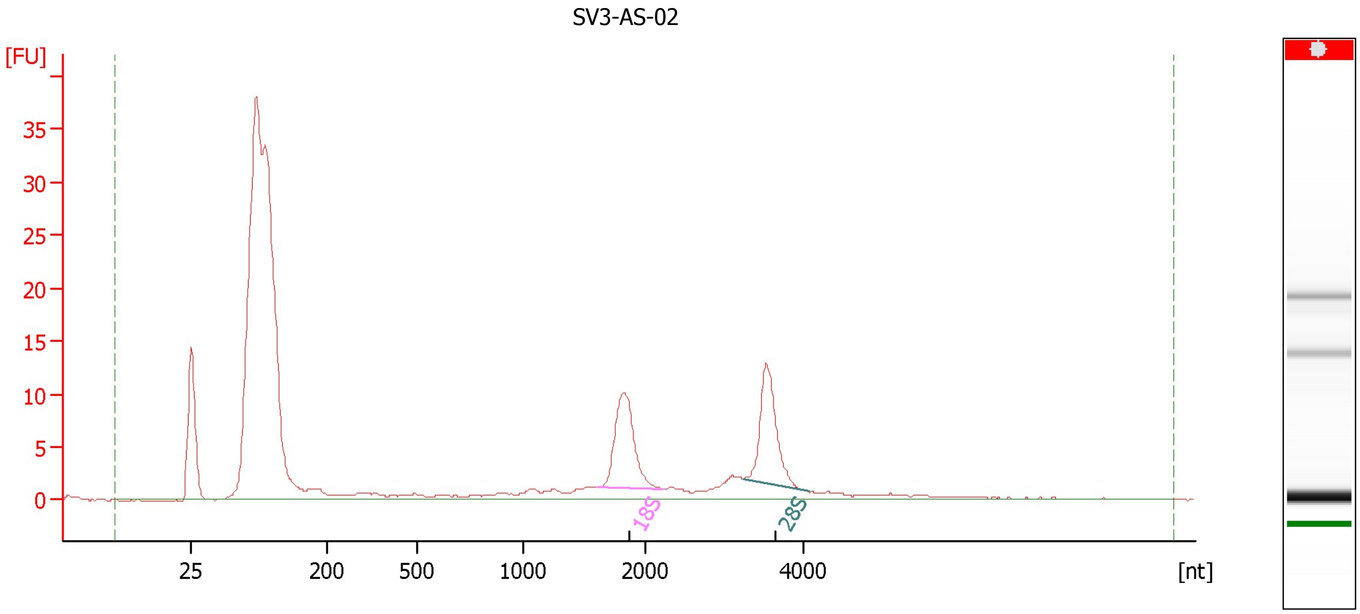


**Overall Results for sample 5 :** **SV3-AS-02**

| | | | |
|---|---|---|---|
| RNA Area: | 281.0 | RNA Integrity Number (RIN): | N/A (B.02.08) |
| RNA Concentration: | 247 ng/µl | Result Flagging Color: | |
| rRNA Ratio [28s / 18s]: | 0.9 | Result Flagging Label: | RIN N/A |

**Fragment table for sample 5 :** **SV3-AS-02**

| Name | Start Size [nt] | End Size [nt] | Area | % of total Area |
|---|---|---|---|---|
| 18S | 1,607 | 2,222 | 21.6 | 7.7 |
| 28S | 3,250 | 4,075 | 19.5 | 7.0 |

 © Copyright 2003 - 2009 Agilent Technologies, Inc. 

Assay Class: Eukaryote Total RNA Nano
Data Path: C:\...Eukaryote Total RNA Nano_DE13804763_2020-08-12_11-44-08.xad

Created: 8/12/2020 11:44:08 AM
Modified: 8/12/2020 12:24:19 PM

**Electropherogram Summary Continued ...**

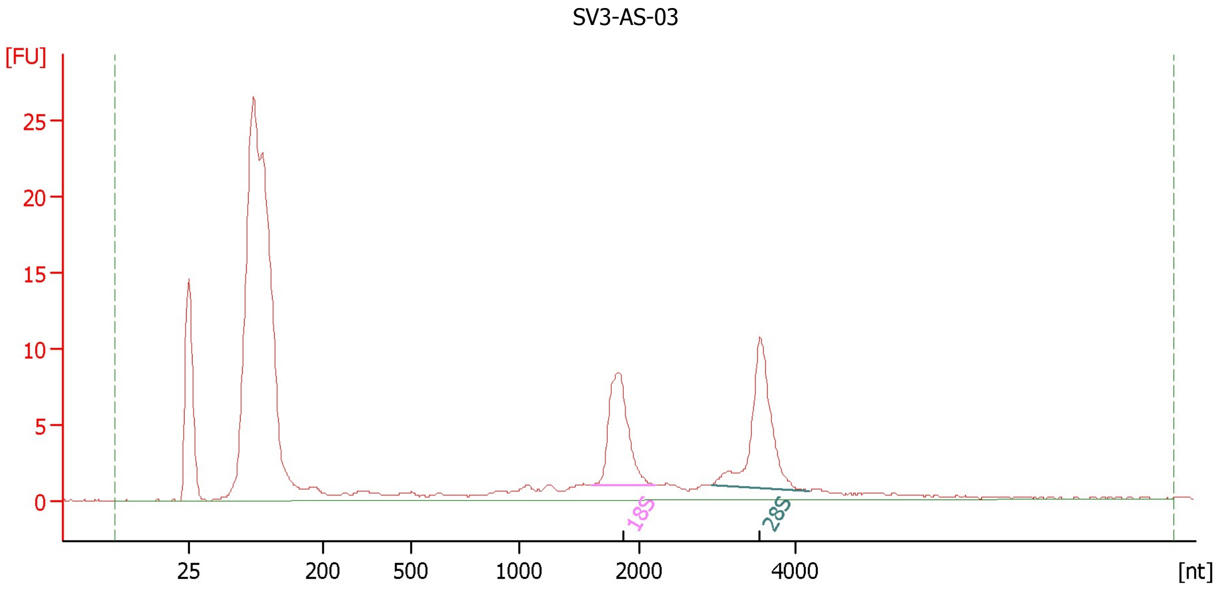


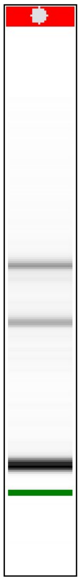

**Overall Results for sample 6 : SV3-AS-03**

| | | | |
|---|---|---|---|
| RNA Area: | 213.4 | RNA Integrity Number (RIN): | N/A (B.02.08) |
| RNA Concentration: | 187 ng/µl | Result Flagging Color: | |
| rRNA Ratio [28s / 18s]: | 1.2 | Result Flagging Label: | RIN N/A |

**Fragment table for sample 6 : SV3-AS-03**

| Name | Start Size [nt] | End Size [nt] | Area | % of total Area |
|---|---|---|---|---|
| 18S | 1,611 | 2,207 | 18.0 | 8.4 |
| 28S | 2,915 | 4,167 | 21.4 | 10.0 |


© Copyright 2003 - 2009 Agilent Technologies, Inc.

Assay Class: Eukaryote Total RNA Nano
Data Path: C:\...Eukaryote Total RNA Nano_DE13804763_2020-08-12_11-44-08.xad

Created: 8/12/2020 11:44:08 AM
Modified: 8/12/2020 12:24:19 PM

**Electropherogram Summary Continued ...**

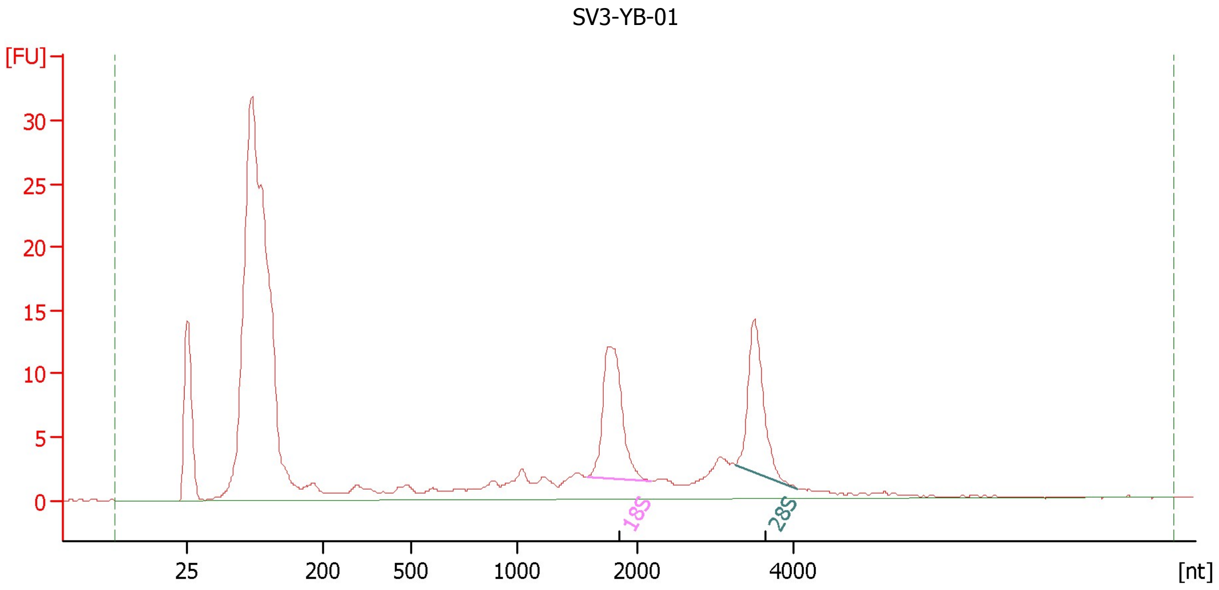


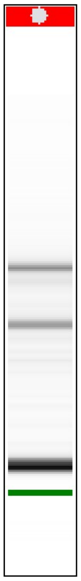

**Overall Results for sample 7 : SV3-YB-01**

| | | | |
|---|---|---|---|
| RNA Area: | 285.0 | RNA Integrity Number (RIN): | N/A (B.02.08) |
| RNA Concentration: | 250 ng/µl | Result Flagging Color: | |
| rRNA Ratio [28s / 18s]: | 0.8 | Result Flagging Label: | RIN N/A |

**Fragment table for sample 7 : SV3-YB-01**

| Name | Start Size [nt] | End Size [nt] | Area | % of total Area |
|---|---|---|---|---|
| 18S | 1,583 | 2,193 | 26.0 | 9.1 |
| 28S | 3,256 | 4,061 | 20.0 | 7.0 |

 © Copyright 2003 - 2009 Agilent Technologies, Inc. 

Assay Class: Eukaryote Total RNA Nano
Data Path: C:\...Eukaryote Total RNA Nano_DE13804763_2020-08-12_11-44-08.xad

Created: 8/12/2020 11:44:08 AM
Modified: 8/12/2020 12:24:19 PM

**Electropherogram Summary Continued ...**

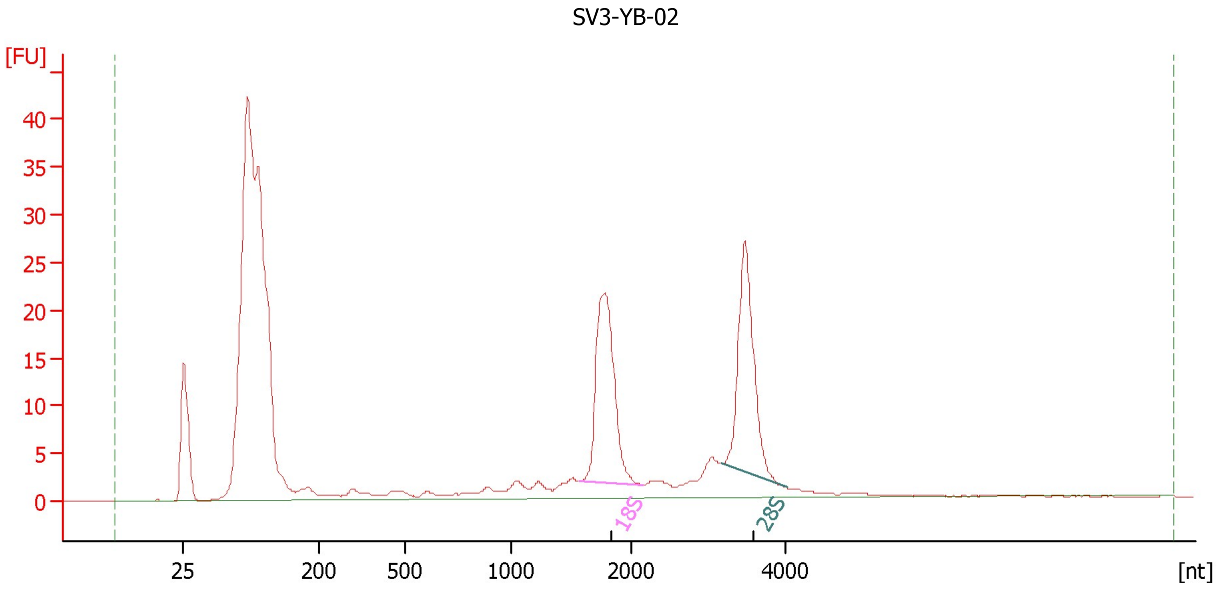


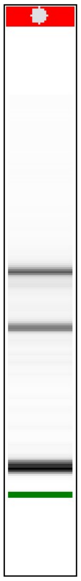

**Overall Results for sample 8 : SV3-YB-02**

| | | | |
|---|---|---|---|
| RNA Area: | 376.1 | RNA Integrity Number (RIN): | N/A (B.02.08) |
| RNA Concentration: | 330 ng/µl | Result Flagging Color: | |
| rRNA Ratio [28s / 18s]: | 0.9 | Result Flagging Label: | RIN N/A |

**Fragment table for sample 8 : SV3-YB-02**

| Name | Start Size [nt] | End Size [nt] | Area | % of total Area |
|---|---|---|---|---|
| 18S | 1,569 | 2,163 | 46.7 | 12.4 |
| 28S | 3,165 | 4,003 | 40.8 | 10.9 |

 © Copyright 2003 - 2009 Agilent Technologies, Inc. 

Assay Class: Eukaryote Total RNA Nano
Data Path: C:\...Eukaryote Total RNA Nano_DE13804763_2020-08-12_11-44-08.xad

Created: 8/12/2020 11:44:08 AM
Modified: 8/12/2020 12:24:19 PM

**Electropherogram Summary Continued ...**

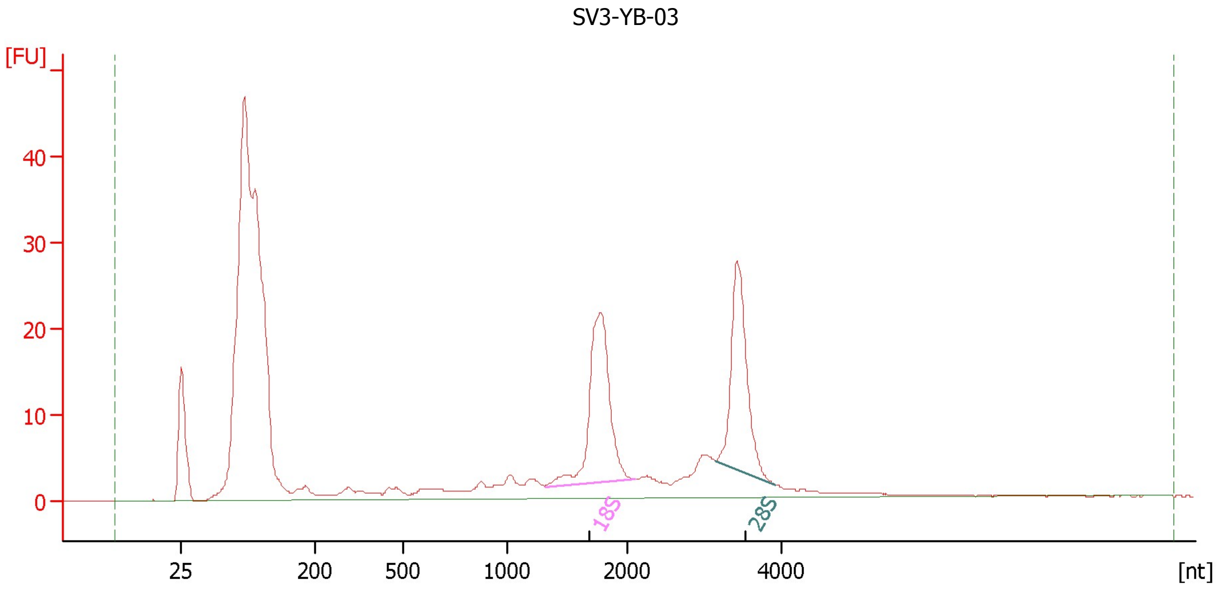


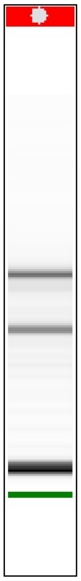

**Overall Results for sample 9 : SV3-YB-03**

| | | | |
|---|---|---|---|
| RNA Area: | 425.7 | RNA Integrity Number (RIN): | N/A (B.02.08) |
| RNA Concentration: | 374 ng/µl | Result Flagging Color: | |
| rRNA Ratio [28s / 18s]: | 0.8 | Result Flagging Label: | RIN N/A |

**Fragment table for sample 9 : SV3-YB-03**

| Name | Start Size [nt] | End Size [nt] | Area | % of total Area |
|---|---|---|---|---|
| 18S | 1,314 | 2,107 | 51.6 | 12.1 |
| 28S | 3,155 | 3,927 | 41.6 | 9.8 |


© Copyright 2003 - 2009 Agilent Technologies, Inc.

| | | | |
|---|---|---|---|
| Assay Class: | Eukaryote Total RNA Nano | Created: | 8/12/2020 11:44:08 AM |
| Data Path: | C:\...Eukaryote Total RNA Nano_DE13804763_2020-08-12_11-44-08.xad | Modified: | 8/12/2020 12:24:19 PM |

**Electropherogram Summary Continued ...**

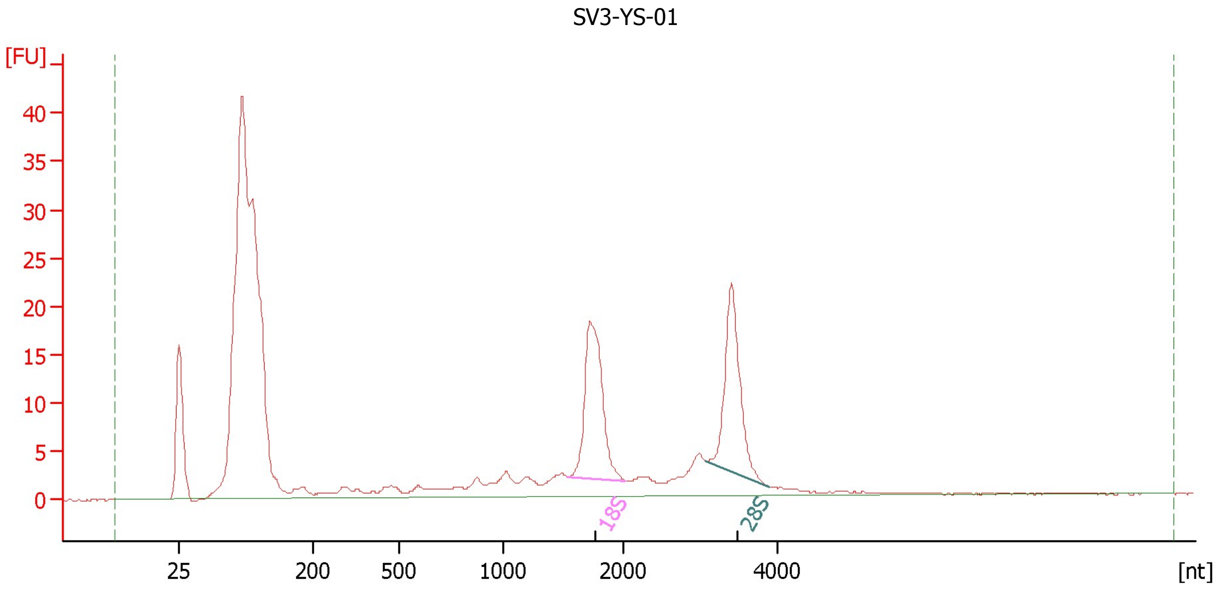


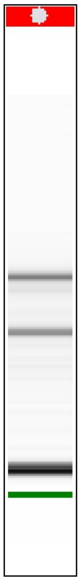

**Overall Results for sample 10 : SV3-YS-01**

| | | | |
|---|---|---|---|
| RNA Area: | 356.8 | RNA Integrity Number (RIN): | N/A (B.02.08) |
| RNA Concentration: | 313 ng/µl | Result Flagging Color: | |
| rRNA Ratio [28s / 18s]: | 0.9 | Result Flagging Label: | RIN N/A |

**Fragment table for sample 10 : SV3-YS-01**

| Name | Start Size [nt] | End Size [nt] | Area | % of total Area |
|---|---|---|---|---|
| 18S | 1,536 | 2,036 | 37.1 | 10.4 |
| 28S | 3,088 | 3,905 | 32.7 | 9.2 |

 © Copyright 2003 - 2009 Agilent Technologies, Inc. 

Assay Class: Eukaryote Total RNA Nano
Data Path: C:\...Eukaryote Total RNA Nano_DE13804763_2020-08-12_11-44-08.xad

Created: 8/12/2020 11:44:08 AM
Modified: 8/12/2020 12:24:19 PM

**Electropherogram Summary Continued ...**

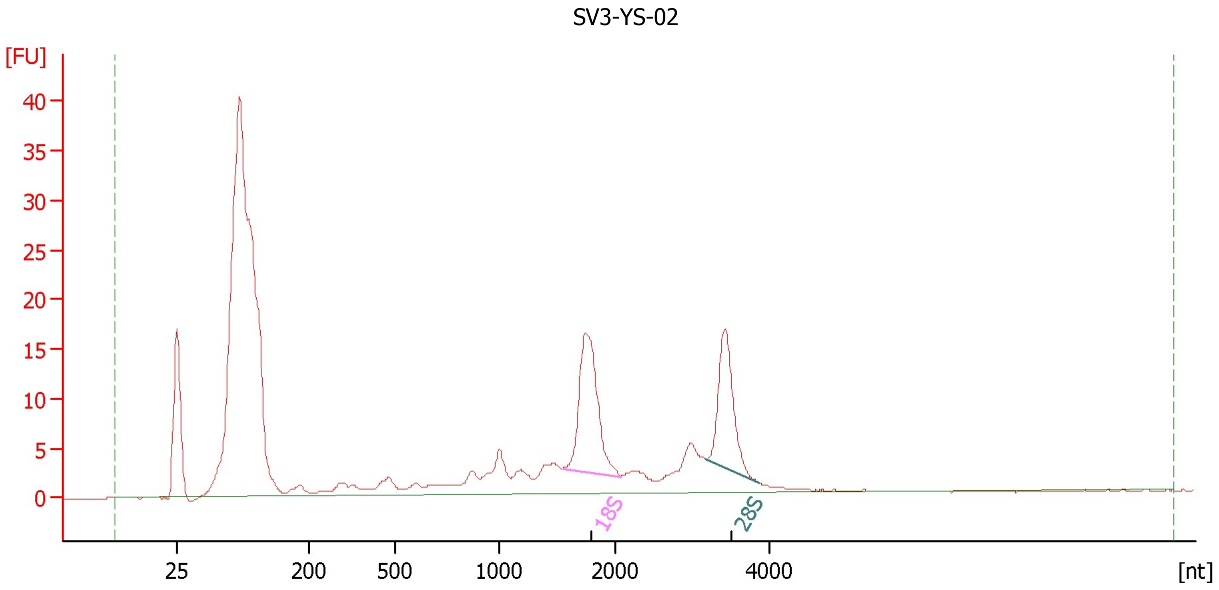


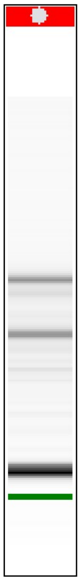

**Overall Results for sample 11 : SV3-YS-02**

| | | | |
|---|---|---|---|
| RNA Area: | 351.6 | RNA Integrity Number (RIN): | N/A (B.02.08) |
| RNA Concentration: | 309 ng/µl | Result Flagging Color: | |
| rRNA Ratio [28s / 18s]: | 0.7 | Result Flagging Label: | RIN N/A |

**Fragment table for sample 11 : SV3-YS-02**

| Name | Start Size [nt] | End Size [nt] | Area | % of total Area |
|---|---|---|---|---|
| 18S | 1,548 | 2,060 | 33.0 | 9.4 |
| 28S | 3,176 | 3,859 | 22.9 | 6.5 |

 © Copyright 2003 - 2009 Agilent Technologies, Inc. 

Assay Class: Eukaryote Total RNA Nano
Data Path: C:\...Eukaryote Total RNA Nano_DE13804763_2020-08-12_11-44-08.xad

Created: 8/12/2020 11:44:08 AM
Modified: 8/12/2020 12:24:19 PM

**Electropherogram Summary Continued ...**

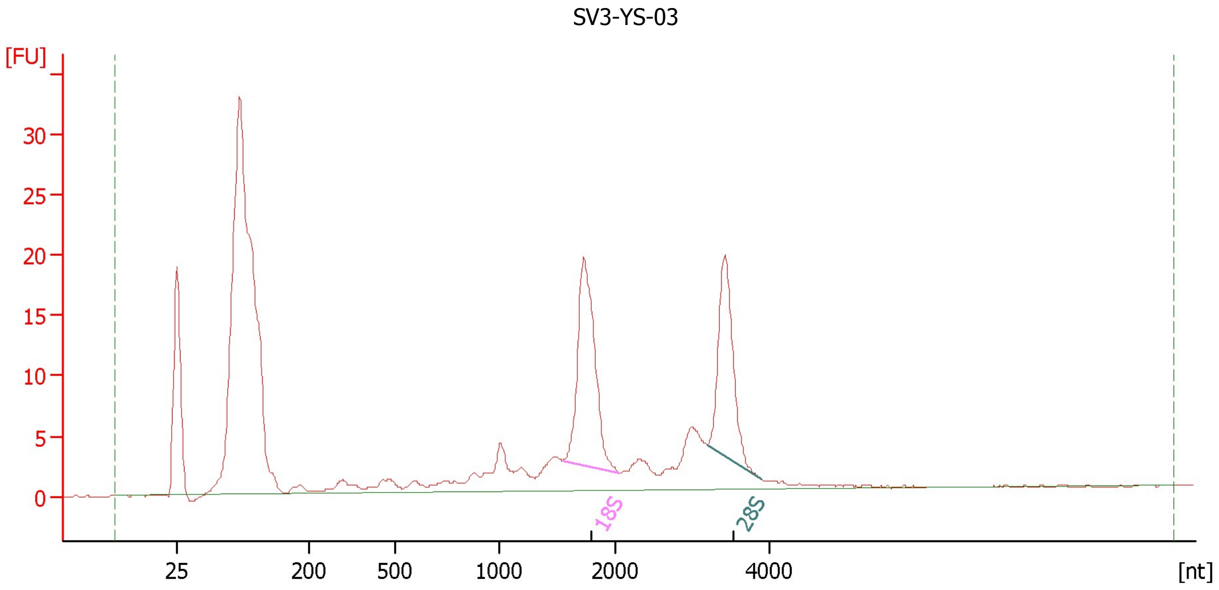


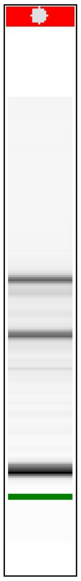

**Overall Results for sample 12 : SV3-YS-03**

| | | | |
|---|---|---|---|
| RNA Area: | 306.8 | RNA Integrity Number (RIN): | N/A (B.02.08) |
| RNA Concentration: | 269 ng/µl | Result Flagging Color: | |
| rRNA Ratio [28s / 18s]: | 0.7 | Result Flagging Label: | RIN N/A |

**Fragment table for sample 12 : SV3-YS-03**

| Name | Start Size [nt] | End Size [nt] | Area | % of total Area |
|---|---|---|---|---|
| 18S | 1,548 | 2,046 | 38.0 | 12.4 |
| 28S | 3,190 | 3,901 | 27.0 | 8.8 |

 © Copyright 2003 - 2009 Agilent Technologies, Inc. 

Assay Class: Eukaryote Total RNA Nano
Data Path: C:\...Eukaryote Total RNA Nano_DE13804763_2020-08-12_11-44-08.xad
Created: 8/12/2020 11:44:08 AM
Modified: 8/12/2020 12:24:19 PM

**Gel Image**

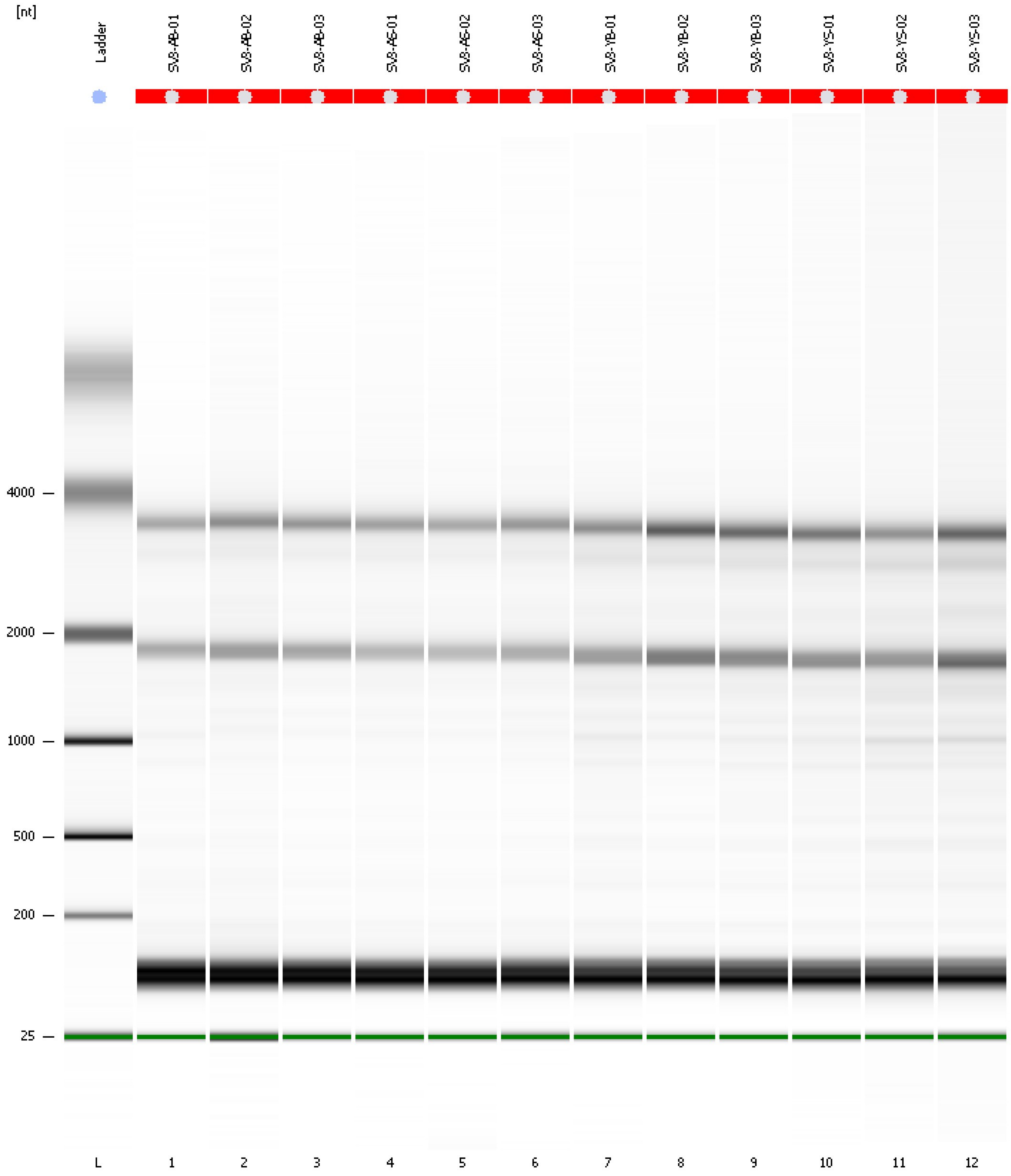


 © Copyright 2003 - 2009 Agilent Technologies, Inc.
