# Supplementary figures and images for: Skeletal muscle transcriptome is affected by age in severely burned mice
Source: Sci Rep. 2022 Dec 14;12:21584. doi: 10.1038/s41598-022-26040-1 (PMC9748408; doi:10.1038/s41598-022-26040-1)

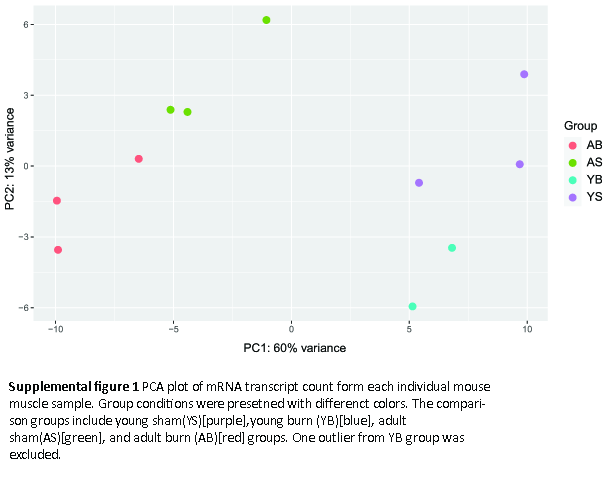

Supplement: Supplementary file 5 — Supplementary Information 5. [file 41598_2022_26040_MOESM5_ESM.tif]
